# Topical Application of Metal Allergens Induces Changes to Lipid Composition of Human Skin

**DOI:** 10.3389/ftox.2022.867163

**Published:** 2022-08-08

**Authors:** Sophie Knox, Lina Hagvall, Per Malmberg, Niamh M. O'Boyle

**Affiliations:** ^1^ School of Pharmacy and Pharmaceutical Sciences, Panoz Institute, Trinity College Dublin, Dublin, Ireland; ^2^ Department of Dermatology and Venereology, Institute of Clinical Sciences, Sahlgrenska Academy, University of Gothenburg, Gothenburg, Sweden; ^3^ Chemistry and Chemical Engineering, Chalmers University of Technology, Gothenburg, Sweden

**Keywords:** metal allergens, contact allergy, skin allergy, ToF-SIMS, skin lipids, cholesterol, triacylglycerols, diacylglycerols

## Abstract

Lipids are an important constituent of skin and are known to be modified in many skin diseases including psoriasis and atopic dermatitis. The direct effects of common metallic contact allergens on the lipid composition of skin has never been investigated, to the best of our knowledge. We describe skin lipid profiles in the stratum corneum and viable epidermis of *ex vivo* human skin from a female donor upon exposure to three metal allergens (nickel, cobalt and chromium) visualised using time-of-flight secondary ion mass spectrometry (ToF-SIMS), which allows for simultaneous visualisation of both the allergen and skin components such as lipids. Multivariate analysis using partial least squares discriminant analysis (PLS-DA) indicated that the lipid profile of metal-treated skin was different to non-treated skin. Analysis of individual ions led to the discovery that cobalt and chromium induced increases in the content of diacylglycerols (DAG) in stratum corneum. Cobalt also induced increases in cholesterol in both the stratum corneum and viable epidermis, as well as monoacylglycerols (MAG) in the viable epidermis. Chromium caused an increase in DAG in viable epidermis in addition to the stratum corneum. In contrast, nickel decreased MAG and DAG levels in viable epidermis. Our results indicate that skin lipid content is likely to be altered upon topical exposure to metals. This discovery has potential implications for the molecular mechanisms by which contact allergens cause skin sensitization.

## 1 Introduction

**FIGURE 1:**
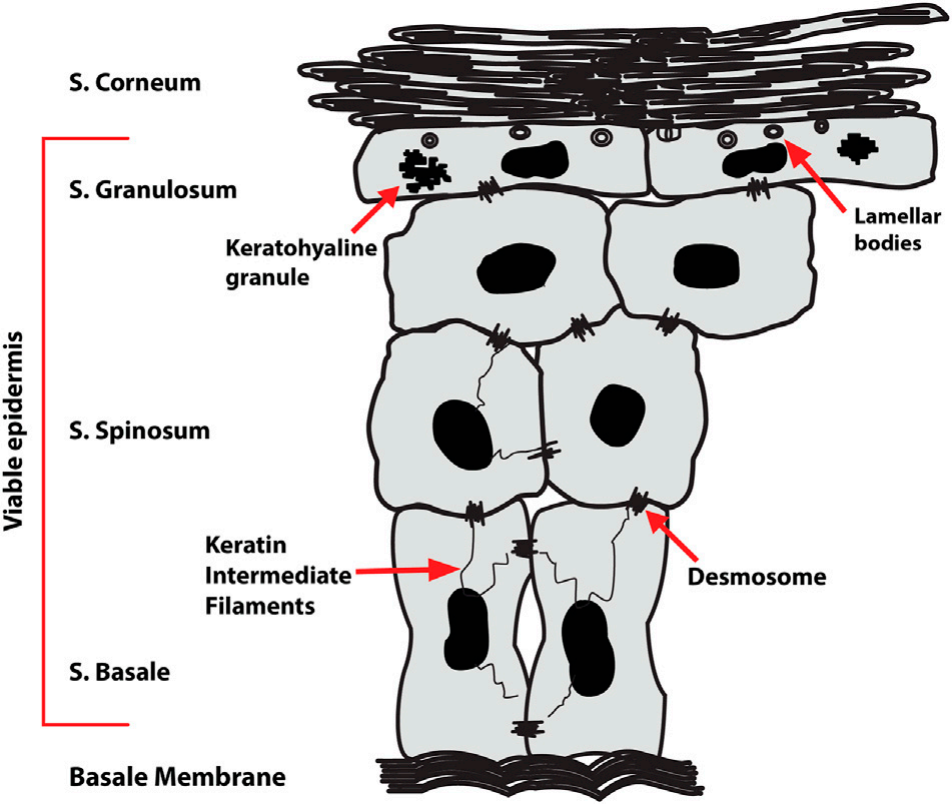
Epidermal structure showing the four sublayers—stratum corneum (SC), stratum granulosum (SG), stratum spinosum (SS) and stratum basale (SB). The viable epidermis consists of the SG, SS, and SB.

Skin contact allergy is the most common form of human immunotoxicity, prevalent in up to a quarter of the European population (Thyssen et al., 2007; Diepgen et al., 2016). It is a type IV delayed hypersensitivity reaction to chemicals known as contact allergens. Allergic contact dermatitis (ACD) is caused by repeated exposure to a contact allergen. It is associated with significant healthcare costs, productivity loss as well as patient suffering and it is one of the leading causes of occupational skin disease. ACD occurs as a cascade of events involving innate and adaptive immune responses, broadly divided into the sensitization and elicitation phases. In the sensitization phase, a contact allergen penetrates skin, stimulates the adaptive immune system and induces memory T cell formation. Contact allergens are thought to act as haptens, too small to stimulate an immune response alone but can do so if they bind to a larger biomolecule. Subsequent exposure to the same or a structurally similar contact allergen triggers the elicitation phase: a delayed hypersensitivity reaction leading to inflammatory symptoms (Kaplan et al., 2012). Protein modification by haptens has been a major focus of research in ACD yet the effects of allergens on skin lipids is unknown. Lipids are a major component of skin, present in the intercellular space and in the corneocyte lipid envelope (CLE). Lipid composition varies in the different skin layers (Figure 1) (Knox and O'Boyle, 2021). For example, stratum corneum (SC) lipids are dominated by ceramides (50% of total composition) but devoid of glycerophospholipids. The vast diversity of lipids plays a key role in the skin’s barrier function, inhibiting excessive transcutaneous evaporative water loss and preventing entry from microbes, allergens and other xenobiotics (Feingold and Elias, 2014). Irregularities in the lipid composition have been studied in multiple skin conditions including psoriasis and atopic dermatitis (Harding, 2004; van Smeden et al., 2014; Sahle et al., 2015; Gruber et al., 2019). The variation in the lipid content in these skin conditions is believed to relate to the reduction in the skin’s integrity and barrier function. However, there is limited research in the importance of lipids in ACD. Considering the critical role of lipids in skin barrier function and structure, it is important to investigate the effects of allergens on skin lipid composition.

Existing research has mainly investigated the human CD1 protein family (CD1a-CD1d), which are lipid-sensing molecules. These proteins are distinct from major histocompatibility (MHC) class I and II proteins, which recognise peptide and protein antigens and are generally believed to contribute to the immune response to contact allergens. Little is known about non-MHC restricted T cell responses in contact allergy. CD1 proteins are expressed on the surface of many immune cells including those resident in the skin, e.g. Langerhans cells and myeloid dendritic cells. The CD1 family can bind and present a wide array of structurally diverse lipids ranging from self-lipids, such as sphingomyelin, to foreign lipid antigens, e.g. mycolic acid from microbes and pentadecylcatechol from poison ivy (Beckman et al., 1994; Moody et al., 2005; Kim et al., 2016). There is some evidence of a role for CD1 in contact allergy, as CD1d knockout mice show an impaired response to the contact sensitizers picryl chloride and dinitrofluorobenzene (Campos et al., 2003; Shimizuhira et al., 2014). CD1d glycolipid antagonists can diminish the immune response to oxazolone as measured by an increase in ear thickness in the murine contact hypersensitivity model (Nieuwenhuis et al., 2005). Recent work shows that small molecule contact sensitizers can lead to CD1-mediated T cell responses which was somehow dependent on endogenous lipids (Betts et al., 2017). Benzyl cinnamate, benzyl benzoate and farnesol were amongst several compounds found to be stimulants of CD1a-restricted T cells (Nicolai et al., 2020).

Metals are amongst the most common contact allergens (Thyssen and Menné, 2010; Yoshihisa and Shimizu, 2012; Diepgen et al., 2016; Thyssen et al., 2021). Contact allergy to nickel is especially prevalent, despite a European nickel directive that limits the rate of nickel release from products that are in close and prolonged contact with skin (Ahlström et al., 2019; Rosholm Comstedt et al., 2020). Cobalt and chromium are also frequently implicated as contact allergens both amongst the general population and in occupational contexts (Uter et al., 2021). Chromium exposure has been frequent in the building industry in cement (as hexavalent chromium), and also in leather (Geier et al., 2011; Aalto-Korte et al., 2020; Hedberg, 2020). Cobalt exposure is also common but the causes have not been fully ascertained, which hampers efforts to prevent ACD to this allergen (Alinaghi et al., 2019). Mechanistically, metal ions including divalent nickel (Ni^2+^) and divalent cobalt (Co^2+^) activate the innate immune system and T cells, with human toll like receptor 4 (TLR4) as a receptor (Schmidt et al., 2010; Raghavan et al., 2012). The nature and extent of the immune response is complex and differs upon exposure to different metals (Dhingra et al., 2014; Schmidt and Goebeler, 2015).

Previous methods have not been able to simultaneously detect both contact allergens and their effects on the skin tissue. We have presented a mass spectrometry imaging-based method able to detect both exogenous and endogenous compounds in skin tissue at the same time (Malmberg et al., 2018; Hagvall et al., 2021). The method is an animal-free approach, as experiments can be performed *ex vivo* using human skin tissue which otherwise would be discarded. Further, it can directly map the distribution of metal and lipid species at nanometer resolution in tissue exposed in a conventional diffusion cell experiment. It is also possible to analyse the distribution in sub-layers of the skin, e.g. the stratum corneum (SC) and viable epidermis (VE). We now describe the effect of three metal allergens—nickel (Ni^2+^), cobalt (Co^2+^) and chromium (Cr^3+^)—on lipids in *ex vivo* human skin using time-of-flight secondary ion mass spectrometry (ToF-SIMS).

## 2 Materials and Methods

### 2.1 Chemicals

Ammonium formate, nickel (II) sulfate hexahydrate (NiSO_4_.6H_2_O) (CAS: 10101-97-0), cobalt (II) chloride hexahydrate (CoCl_2_.6H_2_O) (CAS: 7791-13-1), and chromium (III) chloride hexahydrate (CrCl_3_.6H_2_O) (CAS: 10060-12-5) were purchased from Sigma Aldrich (St Louis, MO, United States).

### 2.2 Skin

Full-thickness human skin was obtained as left-overs from breast reduction surgery (female donor) at the Dept. of Plastic Surgery, Sahlgrenska University Hospital, Gothenburg, Sweden. The tissue was made anonymous upon collection in agreement with routines approved by the local ethics committee. The skin tissue was trimmed from subcutaneous fat, cut into 2 × 2 cm pieces, mounted on cork sheets, wrapped in aluminium foil and kept in −20°C until used within 3 months of surgery.

### 2.3 Skin Exposure

Skin samples from one donor were thawed at room temperature for 30 min prior to experiments. The full thickness skin was mounted in vertical (Franz-type) skin diffusion cells (Laboratory Glass Apparatus, Berkley, CA) having an exposed surface area of 1 cm^2^. The receptor compartments were filled with ammonium formate buffer (0.15 M, pH 7.4, Milli-Q water) compatible with mass spectrometry. Each skin tissue sample was separately exposed to one of the following metal compounds: nickel (II) sulfate hexahydrate (0.15 M), cobalt (II) chloride hexahydrate (0.15M), or chromium (III) chloride hexahydrate (0.15 M). Samples exposed to ammonium formate buffer solution served as controls. The experiment was performed at room temperature (25°C) with an exposure time of 24 h as per OECD standards (OECD-iLibrary, 2022). Experiments were performed in triplicate. At removal, skin samples were gently rinsed with distilled water. Excess water was then removed and the sample was wrapped in aluminium foil and frozen in liquid nitrogen, before sectioning at 10 µm using a Leica CM1520. Sections were placed on conductive glass slides and kept dry until ToF-SIMS analysis.

### 2.4 Instrumentation

ToF-SIMS imaging was performed with a TOF-SIMS V (IONTOF GmbH, Münster, Germany), with a bismuth liquid metal ion gun as a primary ion source and a C_60_ 10-keV ion source as a sputter source. Mass spectra in positive ion mode were recorded by using Bi_3_^+^ primary ions at 25 keV with a pulsed primary ion current of 0.25 pA. Delayed extraction mode was employed to obtain images with high spatial (at best 400 nm) and high respective mass resolution (approx. 5000 at *m/z* 300). Multiple images from each section were recorded in areas ranging from approx. 120 µm × 120 µm–450 μm × 450 µm on the skin sections using a raster of 256 × 256-pixels.

### 2.5 Multivariate Analysis

The automated peak selection function in SurfaceLab was used to select over two hundred *m/z* values of control and metal salt treated skin samples in SC and VE (from *m/z* 14 to *m/z* 605). The data was exported to SIMCA (version 16.0, Umetrics, MKS Instruments Ltd.). Data for each sample was imported to SIMCA as ion intensities normalised to the total ion count for that sample. Pareto scaling was performed, and analysis was carried out by partial least squares discriminant analysis (PLS-DA).

### 2.6 Analysis of Lipid Content

Two regions of interest, the SC and VE, were selected using SurfaceLab software version 7 (IONTOF GmbH, Münster, Germany). The SC is generally between 10–20 μM in thickness, with the VE approximately 100–150 μM thick. The spectra of the three control and three metal-treated skin samples were analysed. The spectra were internally calibrated to [C]+ [CH_2_]+ [CH_3_]+, and [C_5_H_12_N]+. Spectra were scaled by total ion counts. Lipid fragments were selected in SurfaceLab using the *m/z* values of each fragment. Due to the low number of samples (*n* = 3) statistical analysis was not performed and data is described qualitatively. LipidMaps and previous research was used for structure identification (Sjövall et al., 2018; LIPID MAPS, 2022). The following ions were analysed: PC headgroup fragments: *m/z* 184 (C_5_H_15_PNO_4_^+^), 224 (C_8_H_19_PNO_4_^+^); cholesterol fragments: *m/z* 369 (C_27_H_45_^+^), 384 (C_27_H_44_O^+^), 385 (C_27_H_45_O^+^); monoacylglycerol (MAG) fragments: *m/z* 313 (C_19_H_37_O_3_^+^; MAG 16:0), 337 (C_21_H_37_O_3_^+^; MAG 18:2); 339 (C_21_H_39_O_3_^+^; MAG 18:1); 341 (C_21_H_41_O_3_^+^; MAG 18:0); diacylglycerol (DAG) fragments: *m/z* 547 (C_35_H_63_O_4_^+^; DAG 32:2), 549 (C_35_H_65_O_4_^+^; DAG 32:1), 551 (C_35_H_67_O_4_^+^; DAG 32:0), 573 (C_37_H_65_O_4_^+^; DAG 34:1), 575 (C_37_H_67_O_4_^+^; DAG 34:2), 577 (C_37_H_69_O_4_^+^; DAG 34:1), 579 (C_37_H_71_O_4_^+^; DAG 34:0), 601 (C_39_H_69_O_4_^+^; DAG 36:3), 603 (C_39_H_71_O_4_^+^; DAG 36:2), 605 (C_39_H_73_O_4_^+^; DAG 36:1).

## 3 Results

The analysis considers two regions of the skin: the stratum corneum (SC) and the ‘viable’ epidermis (VE) encompassing the stratum granulosum (SG), stratum spinosum (SS) and stratum basale (SB) (Figure 1). The content of four lipid classes, phospholipids [phosphatidylcholine (PC) in particular, the most abundant phospholipid in the skin], cholesterol, monoacylglycerols (MAG) and diacylglycerols (DAG) was analysed in both the SC and VE of skin treated with metal salts using positive ion mode. Metal salts used in this study were nickel (II) sulfate hexahydrate in which nickel is present as a divalent ion (Ni^2+^), cobalt (II) chloride hexahydrate in which cobalt is present as a divalent ion (Co^2+^), and chromium (III) chloride hexahydrate in which chromium is present as a trivalent ion (Cr^3+^). The resulting species formed in solution have been reported previously, where Cr^3+^ was shown to be highly solubilized and bioaccessible whereas Ni^2+^ and Co^2+^ were mainly present as solid species (Hagvall et al., 2021).

### 3.1 Multivariate Data Analysis

**FIGURE 2:**
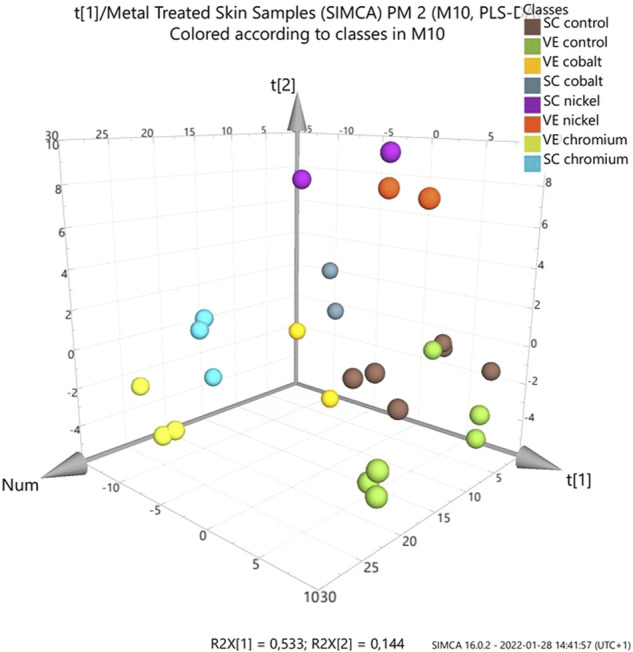
Partial least squares discriminant analysis (PLS-DA) score plot of ToF-SIMS data of metal-treated *ex vivo* human skin. Data was extracted and analysed as normalised intensity (to total ion count) for metal treated skin (nickel, chromium or cobalt) with ammonium formate buffer solution as control. Data was analysed by skin layer (SC and VE). Loadings plot is show in the supplementary information (Supplementary Figure S25).

Multivariate data analysis (MVA) of the control and skin treated with metal compounds was performed to determine if differences existed in their overall lipid profiles in SC and VE (Figure 2). Comparisons were made between all individual samples in positive ion mode, using three skin samples per metal and nine control samples. The results obtained from the PLS-DA show that there is a difference between the lipid content in control skin samples versus those exposed to two of the three metal compounds: Co^2+^ and Cr^3+^. The MVA results show no distinction between the two regions of interest for cobalt and chromium. In contrast, skin treated with nickel was different to cobalt and chromium treated samples.

### 3.2 Exposure of Ex Vivo Skin to Nickel: Lipids in the Stratum Corneum and Viable Epidermis

**FIGURE 3:**
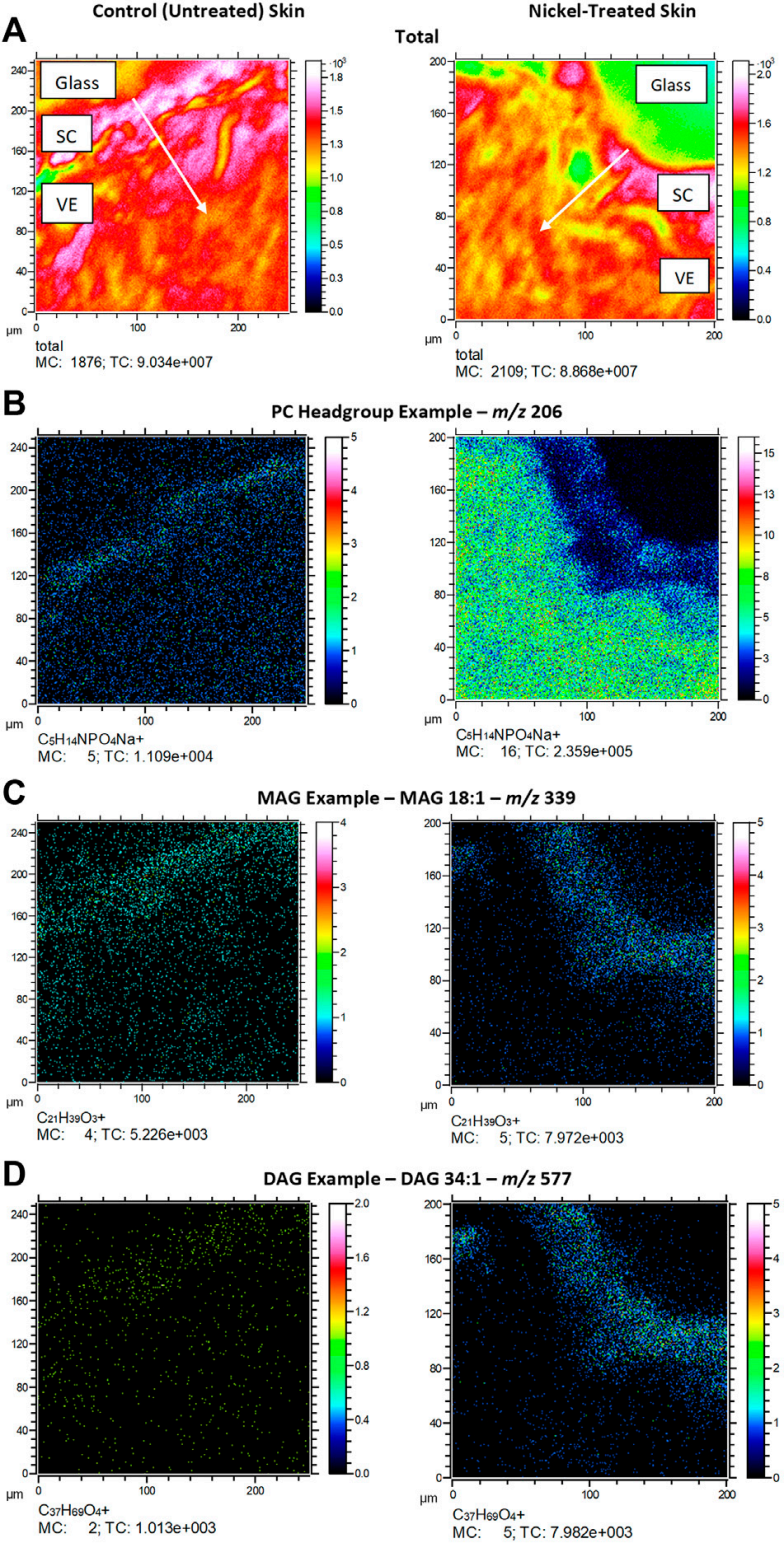
*Ex vivo* skin sections showing **(A)** total ion count; **(B)**. PC headgroup example (*m/z* 206); **(C)**. MAG example (*m/z* 339); **(D)**. DAG example (*m/z* 577). SC = stratum cornuem; VE = viable epidermis. Arrows show direction of chemical penetration from SC to VE. Skin was exposed to nickel (II) sulfate hexahydrate (0.15 M) or ammonium formate buffer solution (control) at room temperature (25°C) for 24 h.

**FIGURE 4:**
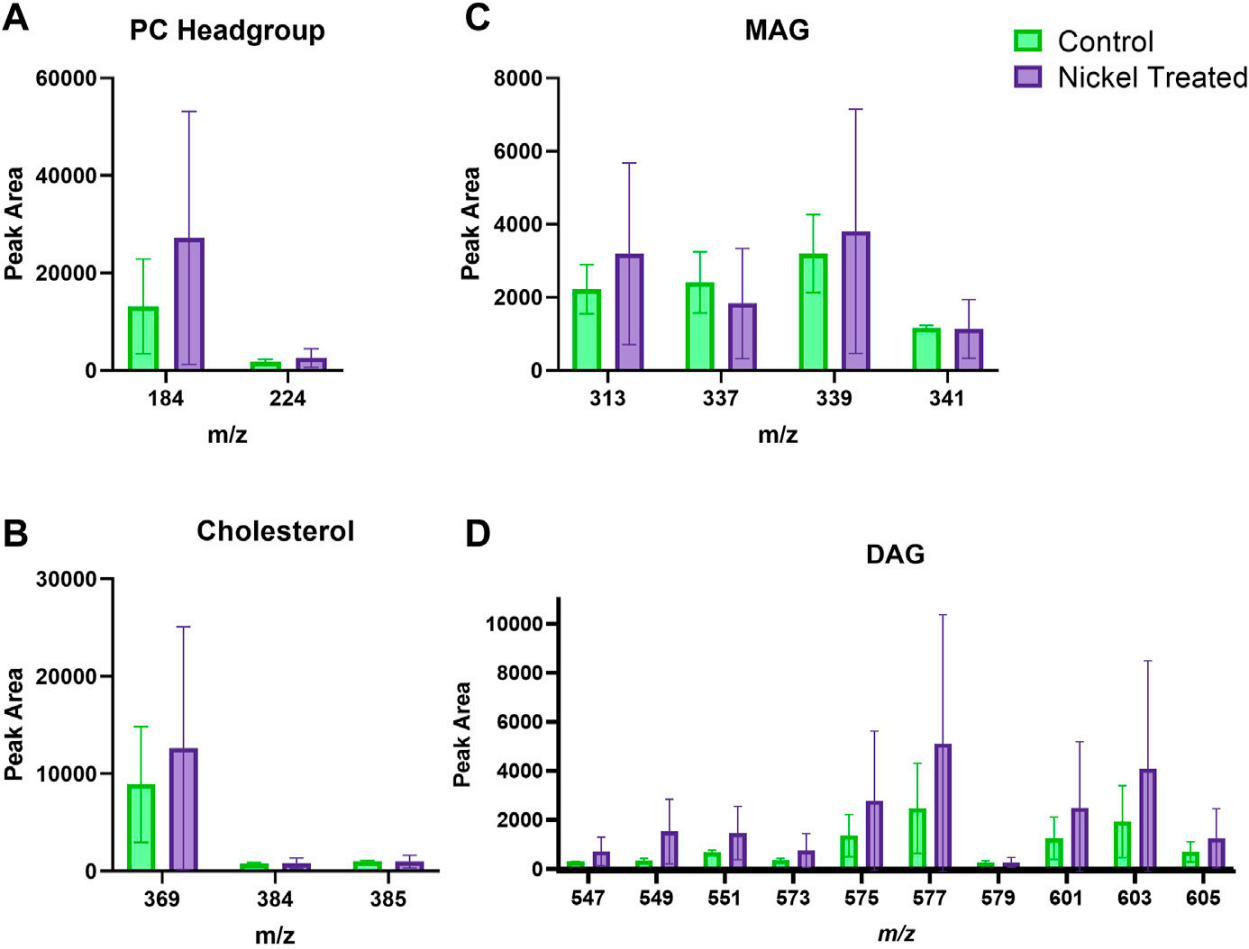
Stratum corneum skin lipid analysis for nickel-treated *ex vivo* human skin. **(A)**. PC headgroup; **(B)**. Cholesterol; **(C)**. Monoacylglycerols; **(D)**. Diacylglycerols. Data was normalised by corrected peak area. Green = control; purple = nickel-treated. Skin was treated with nickel (II) sulfate hexahydrate (0.15 M).

**FIGURE 5:**
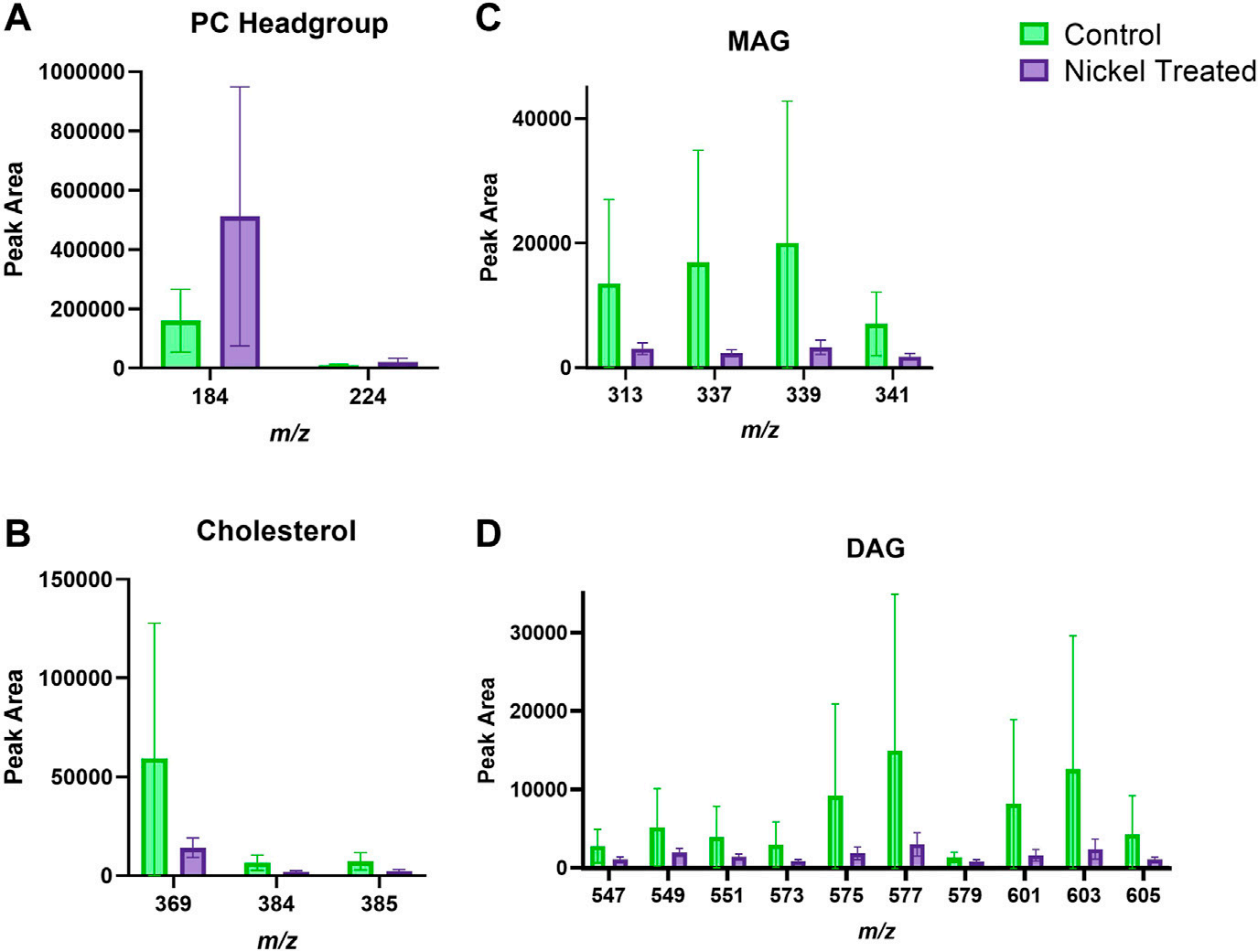
Viable epidermis skin lipid analysis for nickel-treated *ex vivo* human skin. **(A)**. PC headgroup; **(B)**. Cholesterol; **(C)**. Monoacylglycerols; **(D)**. Diacylglycerols. Data was normalised by corrected peak area. Green = control; purple = nickel-treated. Skin was treated with nickel (II) sulfate hexahydrate (0.15 M).

Figure 3 depicts typical *m/z* ion images for skin samples of 200 × 200 μm^2^ and 250 × 250 μm^2^ treated with either nickel or control. The images depict either the total ion count (top), or selected examples from PC headgroup (*m/z* 184), MAG (*m/z* 339) and DAG (*m/z* 577) in control and nickel-treated skin samples. Lipid fragments in nickel-treated skin samples, including PC headgroup, cholesterol, DAG and MAG were similar to the SC compared to control (Figure 4). There was a trend towards decreases in DAG and MAG fragments in VE of nickel-treated skin samples compared to control (Figure 5). Cholesterol (*m/z* 369) appears decreased compared to control in the VE only. The PC headgroup (*m/z* 184) appears increased in the VE. Levels of the cholesterol and PC headgroup fragments are comparable in the VE of both the control and nickel-treated skin samples.

### 3.3 Exposure of Ex Vivo Skin to Chromium: Lipids in the Stratum Corneum and Viable Epidermis

**FIGURE 6:**
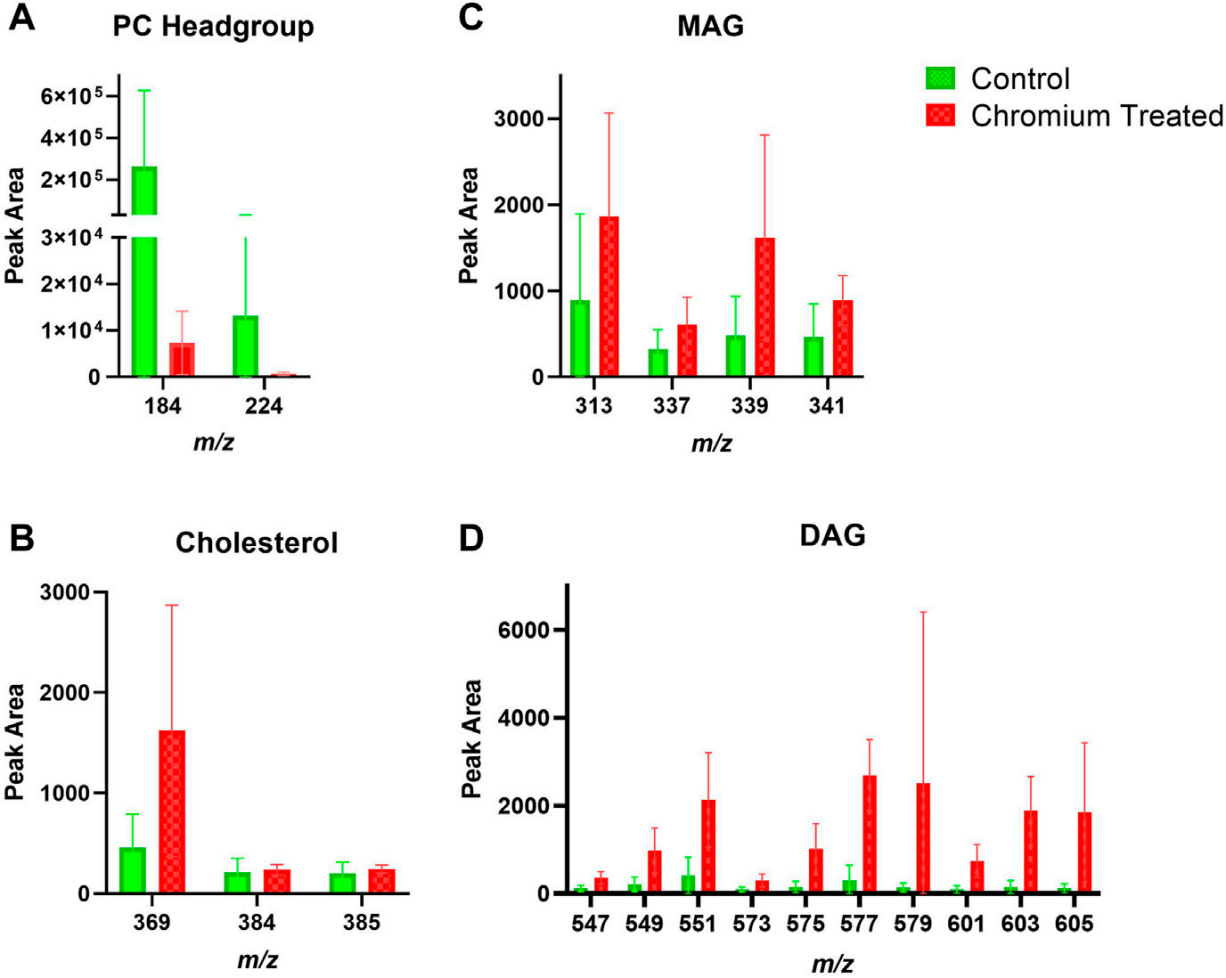
Stratum corneum skin lipid analysis for chromium-treated *ex vivo* human skin. **(A)**. PC headgroup; **(B)**. Cholesterol; **(C)**. Monoacylglycerols; **(D)**. Diacylglycerols. Data was normalised by corrected peak area in positive ion mode. Green = control; red = chromium-treated. Skin was treated with chromium (III) chloride hexahydrate (0.15 M).

**FIGURE 7:**
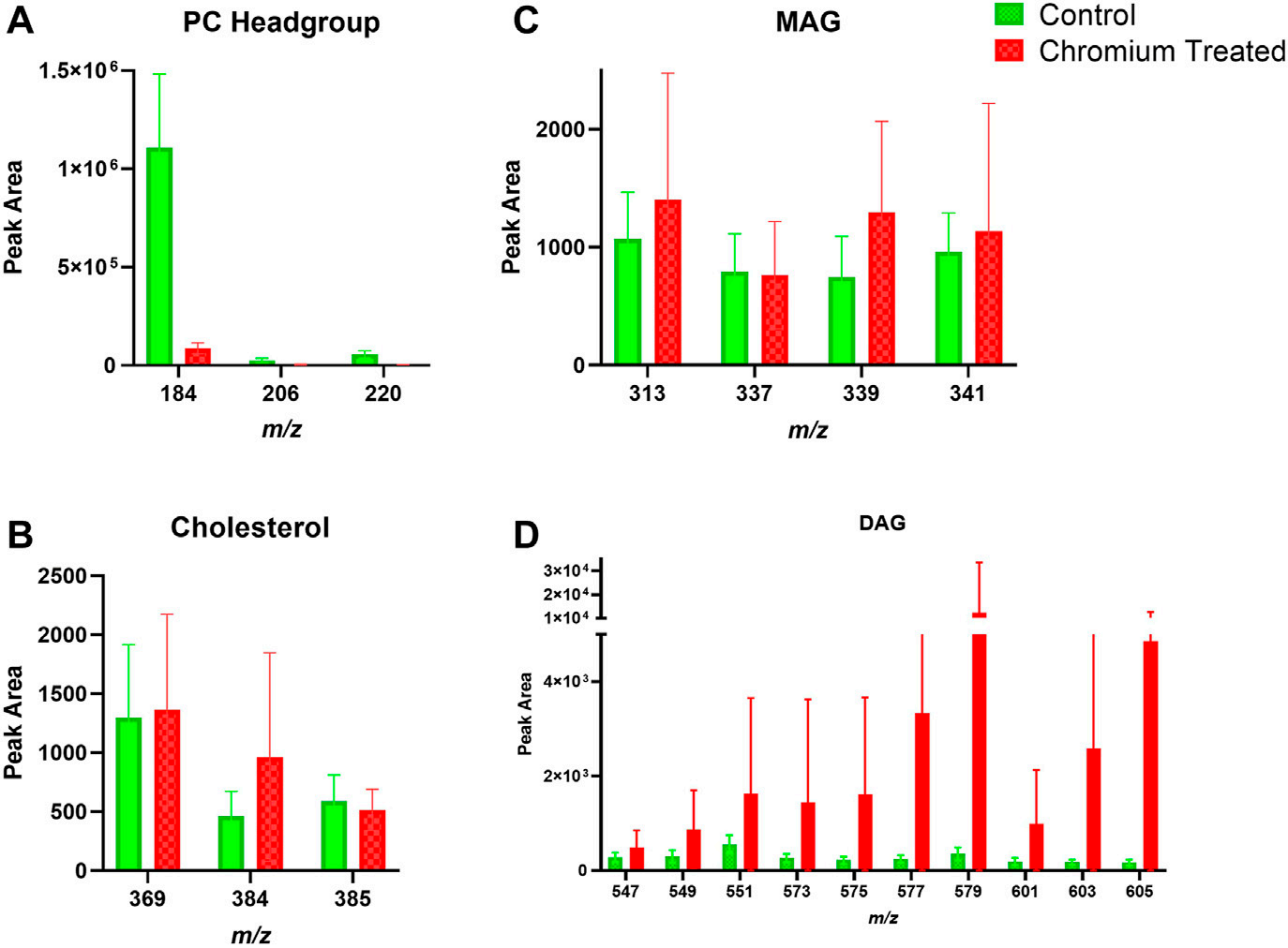
Viable epidermis skin lipid analysis for chromium-treated *ex vivo* human skin. **(A)**. PC headgroup; **(B)**. Cholesterol; **(C)**. Monoacylglycerols; **(D)**. Diacylglycerols. Data was normalised by corrected peak area in positive ion mode. Green = control; red = chromium-treated. Skin was treated with chromium (III) chloride hexahydrate (0.15 M).

Skin samples that were treated with chromium showed a trend of increased amounts in the amount of DAG fragments in the SC (Figure 6). The PC headgroup (*m/z* 184) is decreased compared to control in the SC and VE (Figure 6, Figure 7). Other lipid fragments including MAG and cholesterol did not appear different in the SC. Skin samples that were treated with chromium showed an increased amount of DAG fragments in the VE (Figure 7). Other lipid fragments including PC headgroup, MAG and cholesterol did not appear to be different in the VE of chromium-treated skin compared to control.

### 3.4 Exposure of Ex Vivo Skin to Cobalt: Lipids in the Stratum Corneum and Viable Epidermis

**FIGURE 8:**
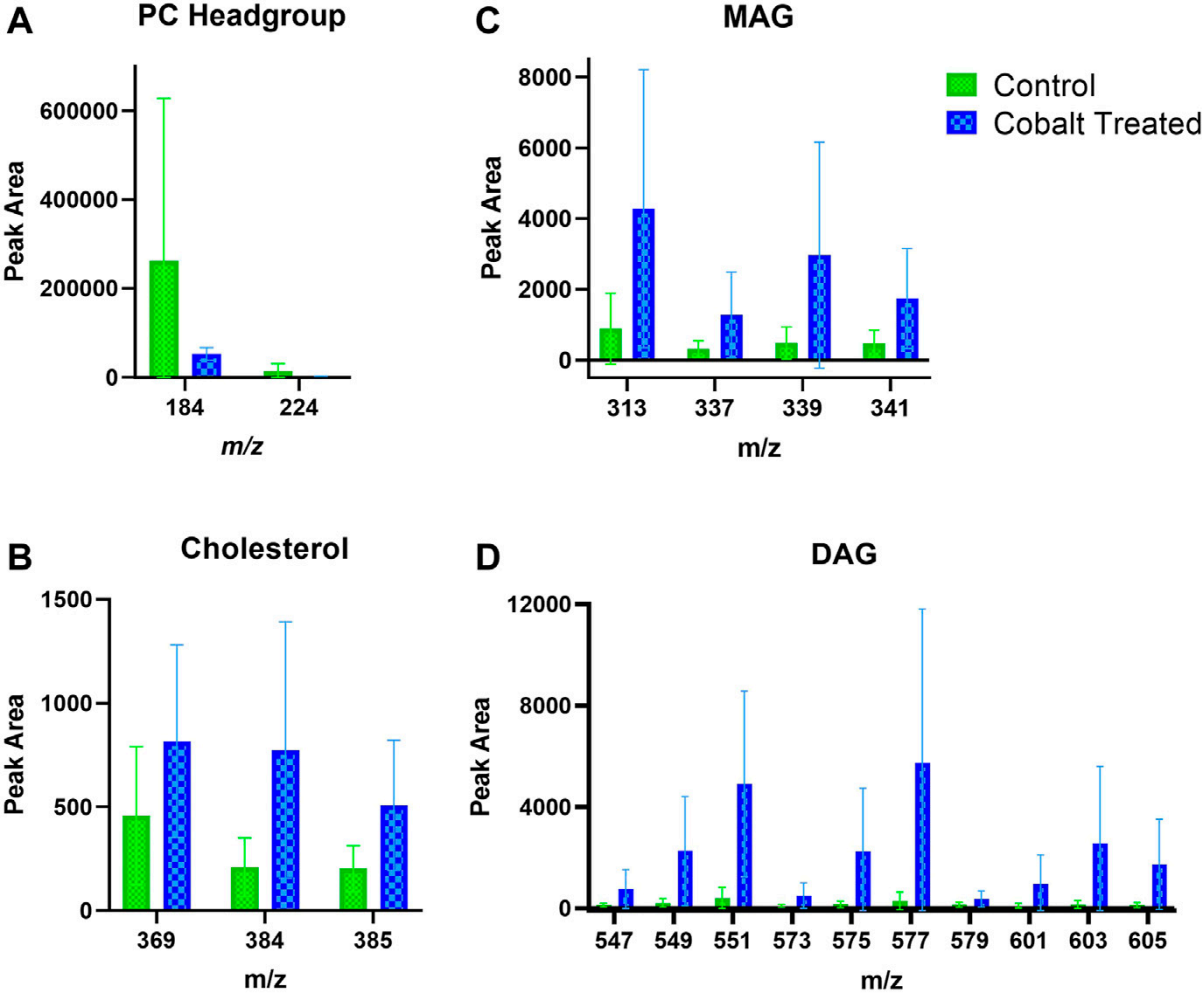
Stratum corneum skin lipid analysis for cobalt-treated *ex vivo* human skin. **(A)**. PC headgroup; **(B)**. Cholesterol; **(C)**. Monoacylglycerols; **(D)**. Diacylglycerols. Data was normalised by corrected peak area in positive ion mode. Green = control; blue = cobalt-treated. Skin was treated with cobalt (II) chloride hexahydrate (0.15 M).

Skin samples that were treated with cobalt showed a trend towards increased amounts of DAG fragment in the SC (Figure 8). There was also an apparent increase in the content of cholesterol in the SC after exposure to cobalt species. This differs compared to the other metal species (chromium and nickel). The levels of PC headgroup and MAG remained similar in both the control and cobalt treated skin samples in the SC.

**FIGURE 9:**
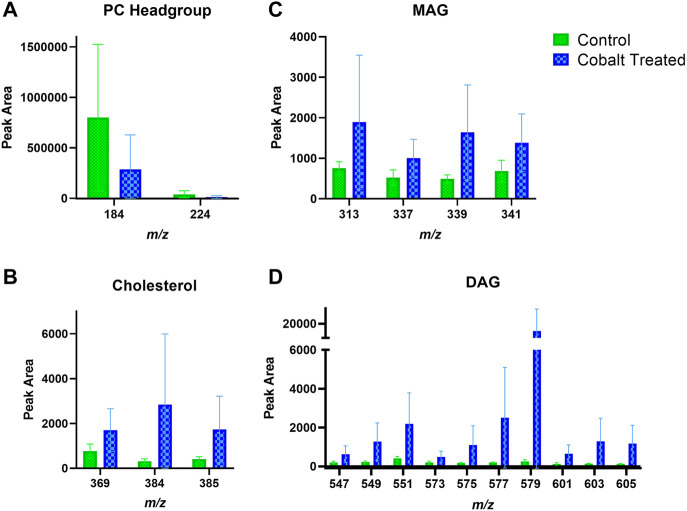
Viable epidermis skin lipid analysis for cobalt-treated *ex vivo* human skin. **(A)**. PC headgroup; **(B)**. Cholesterol; **(C)**. Monoacylglycerols; **(D)**. Diacylglycerols. Data was normalised by corrected peak area in positive ion mode. Green = control; blue = cobalt-treated. Skin was treated with cobalt (II) chloride hexahydrate (0.15 M).

There is also an apparent increase in cholesterol and DAG fragments in the VE upon treatment with cobalt ions (Figure 9). The change in MAG levels was not as pronounced as DAG. Like in the SC, the levels of the PC headgroup fragments remained similar in both the control and cobalt-treated skin samples in the VE.

## 4 Discussion

We analysed the lipid composition of *ex vivo* human skin exposed to salts of three common metallic contact allergens: nickel (Ni^2+^), chromium (Cr^3+^) and cobalt (Co^2+^). ToF-SIMS is particularly suited to detection of metal ions. Results are summarised in Table 1. Skin is classified into stratified layers, with the epidermis being the outermost (Figure 1). The epidermis in turn is subdivided into further layers based on the differentiation of the keratinocytes. In this analysis, we considered two different regions of the skin—the SC and VE. It is noteworthy that data from human biological samples can be extremely variable, and skin lipid content can vary by many factors including age and anatomical location. All samples in our experiments originated from the same donor of skin tissue. This somewhat reduces variability that would usually be expected from differences in anatomical location, age and gender of the sample donor.

**TABLE 1:** Summary of the changes that occur when skin samples are treated with metal ions ***ex vivo*** (positive ion mode; *n* = 3). *MAG: monoacylglycerols; DAG: diacylglycerols; Nd: no notable difference.*

	PC headgroup	Cholesterol	MAG	DAG	PC headgroup	Cholesterol	MAG	DAG
		Stratum corneum				Viable epidermis			
Nickel (Ni^2+^)	Nd	Nd	Nd	Nd	↑	↓	↓	↓
Chromium (Cr^3+^)	↓	Nd	Nd	↑	↓	Nd	Nd	↑
Cobalt (Co^+^)	↓	Nd	↑	↑	↓	↑	↑	↑

It is important to place our results in the context of relative lipid composition of the different skin layers. The outermost skin layer, the SC, consists of anucleate protein-rich corneocytes embedded in a lipid matrix. There are three major lipid subclasses: ceramides (50%), cholesterol (27%), and free fatty acids (10%) (Knox and O'Boyle, 2021). The SC contains negligible amounts of phospholipids and it is perhaps unsurprising that the PC headgroup and phospholipid fragment ions remained consistent in both the control and metal treated skin samples. Cobalt was the only metal compound to induce changes on cholesterol content of the SC. The largest changes were to the profile of DAG, which were elevated in chromium- and cobalt-treated samples. DAG ions may represent triacylglycerols (TAG), as TAG is effectively fragmented in ToF-SIMS yielding mainly DAG ions (Sjövall et al., 2015). It is also likely that MAG ions are formed in a similar process. TAG are a minor component of the lipid matrix in the SC. Nonetheless, the metabolism of TAG is influential in epidermal differentiation and the skin’s barrier function (Radner and Fischer, 2014). Overall, the results indicate changes in the SC of metal-allergen treated skin compared to control, with differences depending on the specific metal allergen tested.

The VE is a composite term describing the th ree remaining epidermal layers: the SG, the SS and the innermost SB (Figure 1) (van Smeden et al., 2014) Each sublayer has distinct characteristics. Although widely referred to as ‘viable’, these epidermal layers are avascular. Hence, despite being excised, the *ex vivo* skin tissue used in our study is a representative model for studying the effects of metal allergens on skin lipids. However, the skin was subjected to a freeze/thaw cycle that would affect enzymatic activity. There were no observed artifacts in the samples, such as cracks caused by ice crystals, caused by the freeze/thaw process. The SB is made up mostly of phospholipids (70%), cholesterol and derivatives (13%), and TAG (11%). Progressive differentiation of keratinocytes alters the lipid composition and, in the SG, intracellular organelles termed lamellar bodies are formed in which glucosylceramides, phospholipids and sphingomyelin are stored (Jiang and Feingold, 2011). Our results show differences for all three metal compounds on different lipid subclasses in both SC and VE.

### 4.1 Nickel

Interestingly, there was a decrease in MAG and DAG fragments in the nickel-treated sample compared to the control skin sample, in the VE (Figure 5). This trend differs to the other metal ions which show an increase in the metal treated skin samples. Differences in the SC between control and nickel-treated samples were not observed (Figure 4). Topically applied water-soluble nickel salts are deposited predominantly in the SC and only a small proportion passes into the VE, independent of the nickel salt used (Hostýnek et al., 2001; Malmberg et al., 2018; Ahlström et al., 2019; Hagvall et al., 2021). The previously published findings from the nickel exposure of the skin tissue in the present study are in line with this (Hagvall et al., 2021). This is in contrast to cobalt and chromium, which penetrate into skin (below). Based on our findings, we speculate that nickel allergy is caused by the small amounts that pass into VE, or alternatively, that nickel act as a catalyst producing products that have immunogenic effects. This would explain the allergenic effects of nickel despite its main localisation in the SC. A combination of these effects is also possible. Ni^2+^ is known to trigger an inflammatory response by directly activating human TLR-4, which is part of the innate immune response (Schmidt et al., 2010). However, if most topically applied nickel remains stuck in the SC, it would appear that only tiny concentrations are required to activate TLR4 in keratinocytes. Interestingly, Ni^3+^ and Ni^4+^ were able to sensitize mice to nickel, in contrast to Ni^2+^ which did not cause sensitization alone. We can speculate that these nickel ions with higher oxidation numbers could be generated from Ni^2+^ in skin by reactive oxygen species (Artik et al., 1999).

### 4.2 Cobalt

Cobalt chloride (Co^2+^) [and potassium dichromate (Cr^6+^)] generally show a different immune activation compared to nickel sulfate (Ni^2+^) (Dhingra et al., 2014). In animal studies of sensitization potency of metal salts, cobalt was found to be a stronger sensitizer than chromium (Basketter et al., 1999). Unlike nickel, cobalt is known to penetrate through skin as it can be detected in urine samples after skin exposure (Scansetti et al., 1994; Kettelarij et al., 2018). Within skin, cobalt distributes throughout the SC and VE after topical application in *ex vivo* human skin (Hagvall et al., 2021). Cobalt skin exposure in occupational environments is unpredictable, e.g. cobalt concentrations on skin of workers at a Swedish hard metals plant after 2 h of work varied enormously between 0.1 and 200 μg/cm^2^/2 h (Wahlqvist et al., 2020). There was an observed increase in the content of cholesterol, DAG and MAG in the SC and VE in cobalt-treated skin samples.

### 4.3 Chromium

Hexavalent chromium (Cr^6+^) is known to penetrate through human skin, whilst trivalent chromium (Cr^3+^) was recently shown to penetrate into skin (Lidén and Lundberg, 1979; Hagvall et al., 2021). The oxidation state of chromium is important, as only trivalent and hexavalent chromium compounds are stable enough to act as haptens (Samitz and Katz, 1964; Gammelgaard et al., 1992). Cr^3+^ compounds are less toxic and allergenic than their Cr^6+^ counterparts, although Cr^6+^ is reduced to Cr^3+^ within skin (Adam et al., 2017). It is unlikely that enzymatic redox activity is still functioning in *ex vivo* skin, hence our choice of Cr^3+^. For example, trivalent chromium did not induce mitochondrial reactive oxygen species production, inflammasome activation, and cytotoxicity in the human monocytic cell line THP-1. In contrast, hexavalent chromium did activate the NLRP3 inflammasome via reactive oxygen species-dependent mechanisms (Adam et al., 2017). Skin samples treated with Cr^3+^ had different content of DAG, both in the SC and the VE, compared to control (Figure 8, Figure 9).

After analysing the effect of metal ions on the SC and VE, there is a distinct difference between the lipid content in the control and metal-treated samples. There is no clear trend for the three allergens and a possible explanation is differences in skin penetration and distribution. Nickel showed distinctly different profiles to cobalt and trivalent chromium, seen clearly in the MVA (Figure 2). Chromium and cobalt caused similar changes to one another. This is interesting considering that cobalt and chromium penetrate much further into human skin than nickel as described below. Overall, the greatest differences were detected in the MAG and DAG fragments.

It is perhaps unsurprising that oxidizing/reducing species such as metals could have an effect on endogenous lipids, which are highly susceptible to peroxidation. The range of lipids analysed was limited to positive ion mode, so not all skin lipids are represented here (e.g. fatty acids). Lipids with higher molecular weights, e.g. ceramides, were also not analysed due to relatively poor ionization at higher *m/z* values. Differing lipid profiles are found *ex vivo* and *in vivo* in other skin diseases such as psoriasis and atopic dermatitis, compared to skin from control subjects (Knox and O'Boyle, 2021). Based on our findings using *ex vivo* human skin, there could also be differences between the lipid profiles of patients with skin allergy to metals compared to control subjects. The study was limited to one skin donor (with three samples from a section of skin, each sample exposed independently) and further studies with different donors and allergens are warranted. Exposure time was 24 h for all allergens; it would be interesting to investigate the effects of longer-term exposure, but this would be difficult with *ex vivo* tissue. Despite the limitations of the model, our results also indicate that skin sensitizers can affect lipids in the VE despite penetration into the VE to a low degree (nickel).

Co-crystal structures of the pentadecylcatechol and farnesol show their mode of binding within CD1a, with displacement of endogenous lipidic ligands (Kim et al., 2016; Nicolai et al., 2020). Both compounds are lipophilic and their interaction mimics endogenous ligands, binding within the A' and F' pockets of the protein. However, the mechanism by which metal allergens and small organic molecules could affect CD1-mediated immune responses remains to be elucidated. Based on the results presented herein, it is possible that allergens induce changes to the balance of endogenous lipids that could trigger these responses but further work is required to establish this. Additionally, lipids including DAGs are involved in cellular signaling and changes in their levels upon allergen exposure could alter these signaling pathways (Mérida et al., 2007).

In conclusion, this study is one of the first where both haptens and their immediate effect on the skin tissue are studied simultaneously. This was possible by use of ToF-SIMS. We have investigated the effects of skin sensitizers nickel (Ni^2+^), cobalt (Co^2+^), and chromium (Cr^3+^) on the lipid profile of *ex vivo* human skin. Changes were observed in both the SC and VE for many lipid classes. MAG and DAG were affected by all metals in at least one of the skin layers analysed, but opposite trends were found for nickel compared to chromium and cobalt. Our results indicate that lipids may not be inert bystanders in the molecular mechanisms leading to skin allergy. Further research is necessary to quantify the effects of these changes in lipid composition in ACD.

## Data Availability

The original contributions presented in the study are included in the article/Supplementary Material, further inquiries can be directed to the corresponding author.
