# Supplementary material for: Topical Application of Metal Allergens Induces Changes to Lipid Composition of Human Skin
Source: Front Toxicol. 2022 Aug 8;4:867163. doi: 10.3389/ftox.2022.867163 (PMC9393847; doi:10.3389/ftox.2022.867163)

**SUPPORTING INFORMATION**

**Metal Allergen Induced Changes to Lipid Composition of Human Skin**

Sophie Knox,^1^ Lina Hagvall,^2^ Per Malmberg,^3^ Niamh M. O’Boyle^1^*

*^1^School of Pharmacy and Pharmaceutical Sciences, Panoz Institute, Trinity College Dublin,* *D02 PN40, Ireland*

*^2^University of Gothenburg, Sweden*

*^3^Chemistry and Chemical Engineering, Chalmers University of Technology, Sweden*

*Corresponding author. Email: [nioboyle@tcd.ie](mailto:nioboyle@tcd.ie); Phone: +353-1896-2524

Contents

[Table S1. *m/z* peak area values for nickel-treated skin in stratum corneum, normalised by corrected peak area (positive ion mode) 3](#_Toc105076032)

[Figure S1: Ion Profile: Nickel Treated, Stratum Corneum: PC Headgroup 5](#_Toc105076033)

[Figure S2: Ion Profile: Nickel Treated, Stratum Corneum: Cholesterol 5](#_Toc105076034)

[Figure S3: Ion Profile: Nickel Treated, Stratum Corneum: MAG 6](#_Toc105076035)

[Figure S4: Ion Profile: Nickel Treated, Stratum Corneum: DAG 6](#_Toc105076036)

[Table S2. *m/z* peak area values for nickel-treated skin in viable epidermis, normalised by corrected peak area (positive ion mode) 7](#_Toc105076037)

[Figure S5: Ion Profile: Nickel Treated, Viable Epidermis: PC Headgroup 9](#_Toc105076038)

[Figure S6: Ion Profile: Nickel Treated, Viable Epidermis: Cholesterol 9](#_Toc105076039)

[Figure S7: Ion Profile: Nickel Treated, Viable Epidermis: MAGs 10](#_Toc105076040)

[Figure S8: Ion Profile: Nickel Treated, Viable Epidermis: DAGs 10](#_Toc105076041)

[Table S4. *m/z* peak area values for chromium-treated skin in stratum corneum, normalised by corrected peak area (positive ion mode) 11](#_Toc105076042)

[Figure S9: Ion Profile: Chromium Treated, Stratum Corneum: PC Headgroup 13](#_Toc105076043)

[Figure S10: Ion Profile: Chromium Treated, Stratum Corneum: Cholesterol 13](#_Toc105076044)

[Figure S11: Ion Profile: Chromium Treated, Stratum Corneum: MAG 14](#_Toc105076045)

[Figure S12: Ion Profile: Chromium Treated, Stratum Corneum: DAG 14](#_Toc105076046)

[Table S5. *m/z* peak area values for chromium-treated skin in viable epidermis, normalised by corrected peak area (positive ion mode) 15](#_Toc105076047)

[Figure S13: Ion Profile: Chromium Treated, Viable Epidermis: PC Headgroup 16](#_Toc105076048)

[Figure S14: Ion Profile: Chromium Treated, Viable Epidermis: Cholesterol 16](#_Toc105076049)

[Figure S15: Ion Profile: Chromium Treated, Viable Epidermis: MAG 17](#_Toc105076050)

[Figure S16: Ion Profile: Chromium Treated, Viable Epidermis: DAG 17](#_Toc105076051)

[Table S6. *m/z* peak area values for cobalt-treated skin in stratum corneum, normalised by corrected peak area (positive ion mode) 18](#_Toc105076052)

[Figure S17: Ion Profile: Cobalt Treated, Stratum Corneum: PC Headgroup 19](#_Toc105076053)

[Figure S18: Ion Profile: Cobalt Treated, Stratum Corneum: Cholesterol 19](#_Toc105076054)

[Figure S19: Ion Profile: Cobalt Treated, Stratum Corneum: MAG 20](#_Toc105076055)

[20](#_Toc105076056)

[Figure S20: Ion Profile: Cobalt Treated, Stratum Corneum: DAG 21](#_Toc105076057)

[Table S7. *m/z* peak area values for cobalt-treated skin in viable epidermis, normalised by corrected peak area (positive ion mode) 22](#_Toc105076058)

[Figure S21: Ion Profile: Cobalt Treated, Viable Epidermis: PC Headgroup 23](#_Toc105076059)

[Figure S22: Ion Profile: Cobalt Treated, Viable Epidermis: Cholesterol 24](#_Toc105076060)

[Figure S23: Ion Profile: Cobalt Treated, Viable Epidermis: MAG 24](#_Toc105076061)

[Figure S24: Ion Profile: Cobalt Treated, Viable Epidermis: DAG 25](#_Toc105076062)

[Figure S25. A. 2D Score plot and B. loadings plot for partial least squares discriminant analysis (PLS-DA) of ToF-SIMS data of metal-treated *ex vivo* human skin. Data was extracted and analysed as normalised intensity (to total ion count). 26](#_Toc105076063)

# Table S1. *m/z* peak area values for nickel-treated skin in stratum corneum, normalised by corrected peak area (positive ion mode)

|  | ***Control Samples*** | | | ***Nickel-Treated Samples*** | | |
| --- | --- | --- | --- | --- | --- | --- |
| ***m/z*** | **Control 1** | **Control 2** | **Control 3** | **Nickel 1** | **Nickel 2** | **Nickel 3** |
| **PC Headgroup** |  |  |  |  |  |  |
| **184** | 22811.26 | 13270.64 | 3343.12 | 19646.85 | 56129.64 | 5846.73 |
| **206** | 4873.14 | 3360.98 | 1572.37 | 3932.07 | 8599.92 | 2438.02 |
| **224** | 2230.23 | 1796.33 | 1162.51 | 1318.4 | 4782.48 | 1556.58 |
|  |  |  |  |  |  |  |
| **Cholesterol** |  |  |  |  |  |  |
| **367** | 1743.11 | 1522.84 | 3773.08 | 644.02 | 3559.63 | 7057.86 |
| **369** | 6148.02 | 4848.17 | 15727.92 | 1656.38 | 10028.99 | 26188.46 |
| **384** | 876.24 | 712.78 | 805.93 | 205.29 | 1213.52 | 1038.69 |
| **385** | 1126.78 | 954.15 | 870.21 | 243.44 | 1471.77 | 1259.19 |
|  |  |  |  |  |  |  |
| **Vitamin E** |  |  |  |  |  |  |
| **430** | 1110.79 | 1122.78 | 595.5 | 257.36 | 1957.06 | 867.15 |
|  |  |  |  |  |  |  |
| **MAG** |  |  |  |  |  |  |
| **313** | 2013.69 | 1682.88 | 2977.56 | 479.41 | 3726.69 | 5365.33 |
| **337** | 2086.54 | 1776.95 | 3364.22 | 284.54 | 1914.14 | 3289.22 |
| **339** | 2795.46 | 2387.38 | 4406.85 | 536.55 | 3669.02 | 7215.35 |
| **341** | 1170.71 | 1069.16 | 1223.16 | 204.41 | 1613.94 | 1582.97 |
|  |  |  |  |  |  |  |
| **DAG** |  |  |  |  |  |  |
| **547** | 307.55 | 295.35 | 287.39 | 108.11 | 791.29 | 1266.82 |
| **549** | 385.25 | 404.7 | 230.38 | 230.4 | 1498.47 | 2860.52 |
| **551** | 651.13 | 624.09 | 771 | 215.35 | 2198.89 | 1972.41 |
| **573** | 383.43 | 276.27 | 410.55 | 80.05 | 716.02 | 1457.15 |
| **575** | 1047.12 | 698.12 | 2326.56 | 252.32 | 2238.02 | 5849.41 |
| **577** | 1705.46 | 1146.82 | 4562.84 | 523.18 | 3969.8 | 10840.12 |
| **579** | 234.73 | 334.82 | 213.61 | 59.1 | 434.69 | 336.28 |
| **601** | 897.66 | 612.93 | 2237.17 | 245.3 | 1750.93 | 5480.18 |
| **603** | 1293.56 | 885.88 | 3611.35 | 385.7 | 2921.3 | 8950.48 |
| **605** | 555.24 | 376.7 | 1162.13 | 152.19 | 1047.63 | 2549.27 |

#
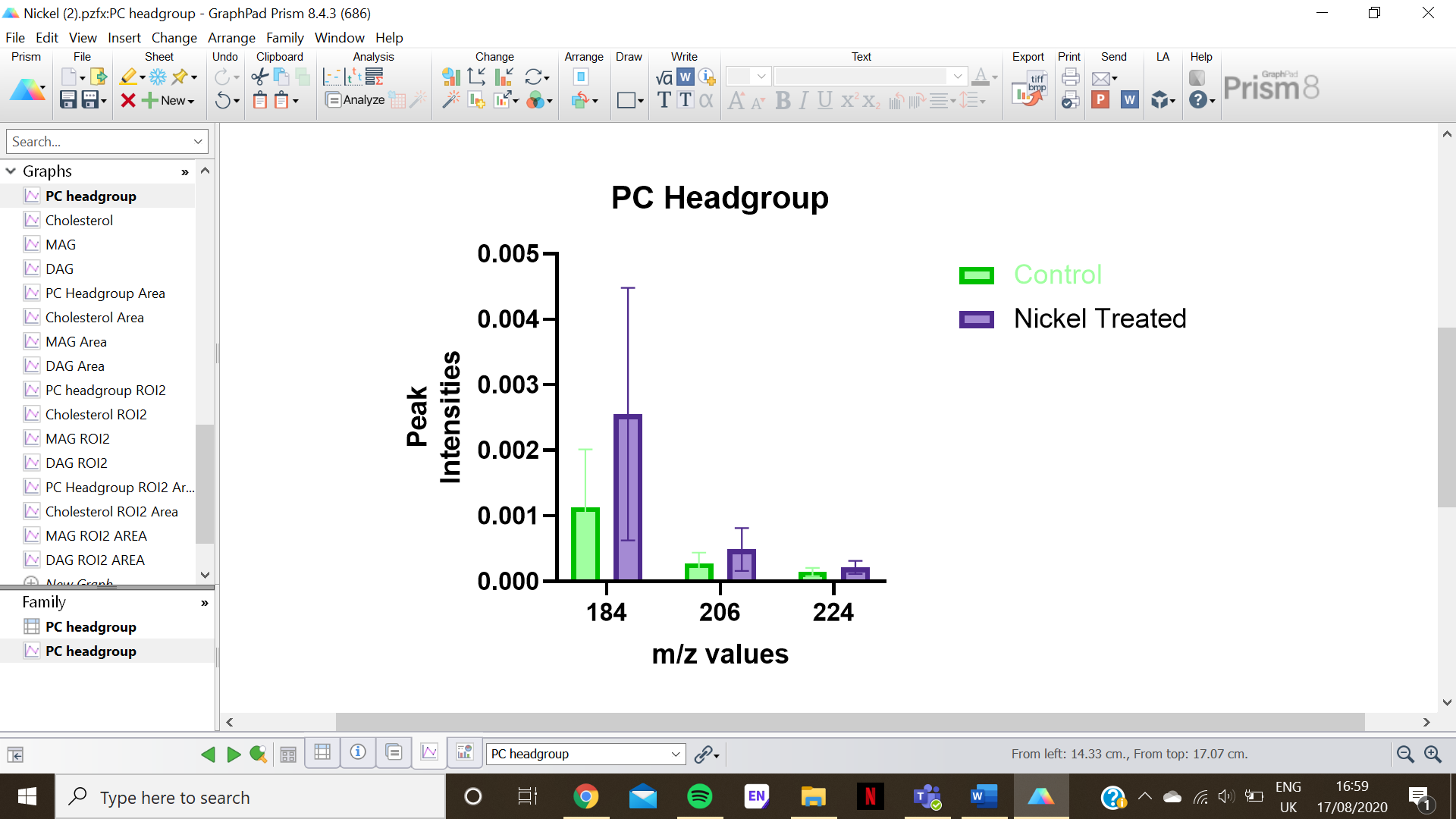

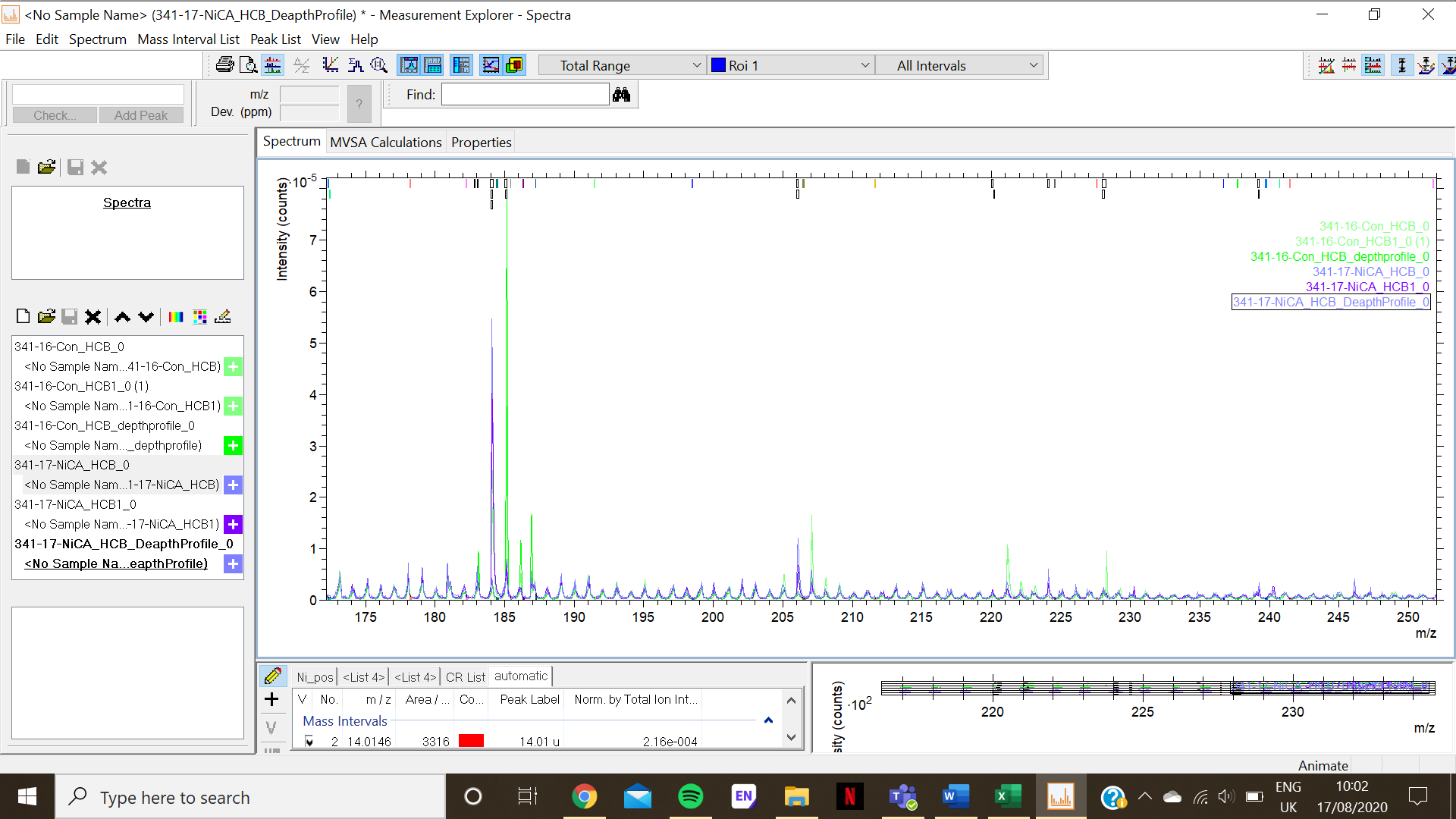
Figure S1: Ion Profile: Nickel Treated, Stratum Corneum: PC Headgroup

224 m/z C_8_H_19_NPO_4_Na

184 m/z C_5_H_15_NPO_4_

206 m/z C_5_H_14_NPO_4_Na

#
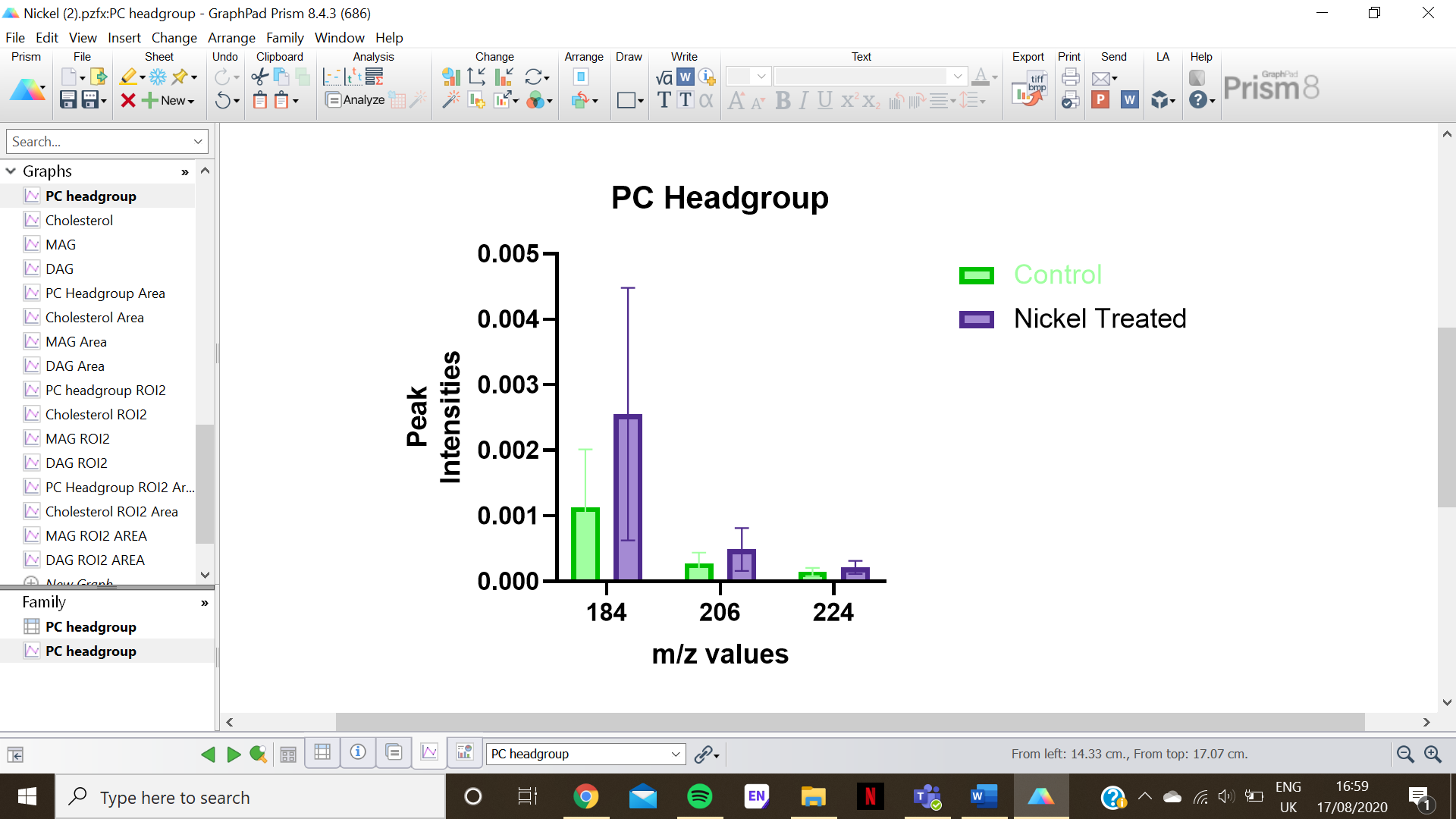

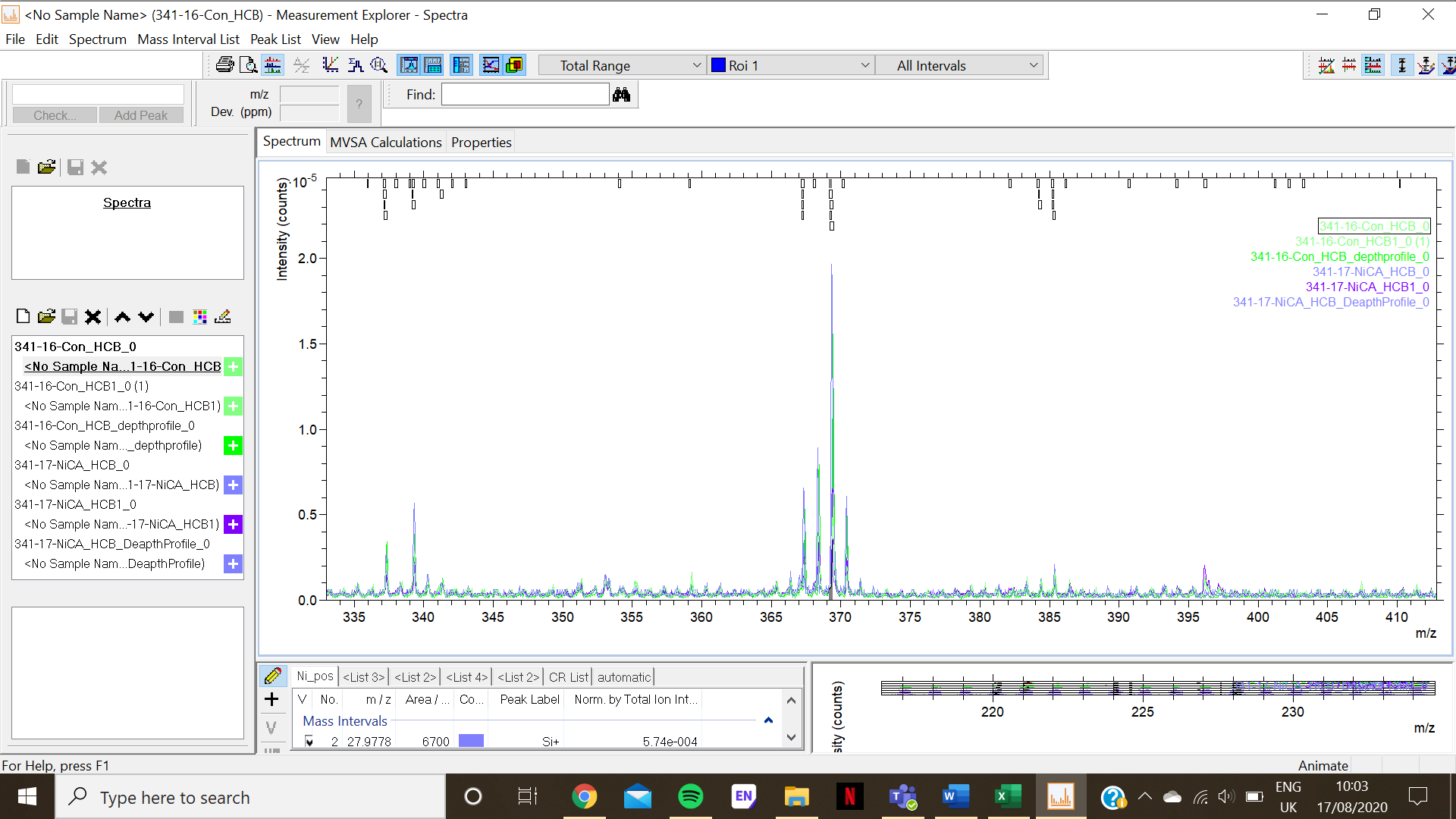
Figure S2: Ion Profile: Nickel Treated, Stratum Corneum: Cholesterol

385 m/z C_27_H_45_O

384 m/z C_27_H_44_O

369 m/z C_27_H_45_

367 m/z C_27_H_43_

#
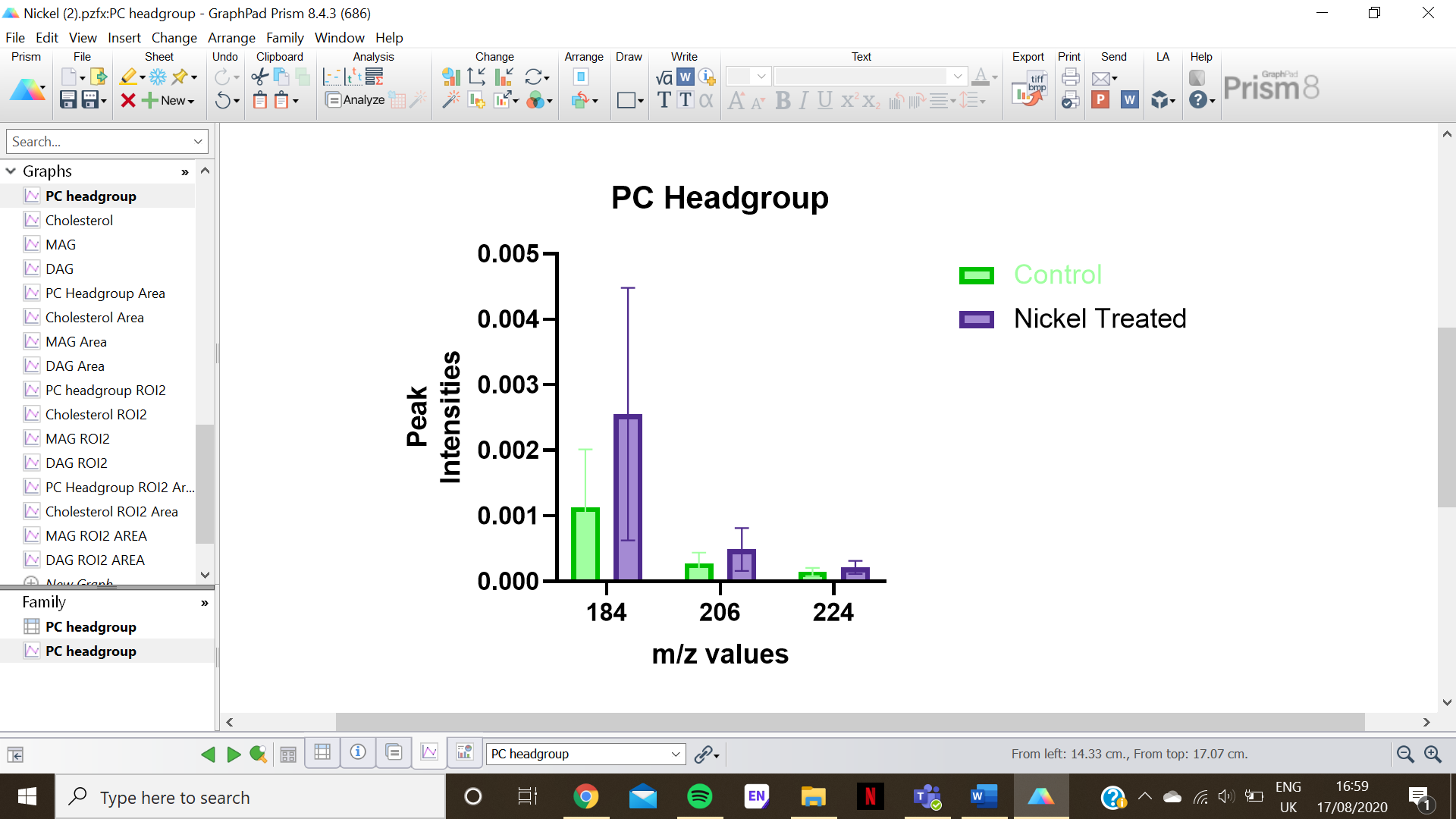

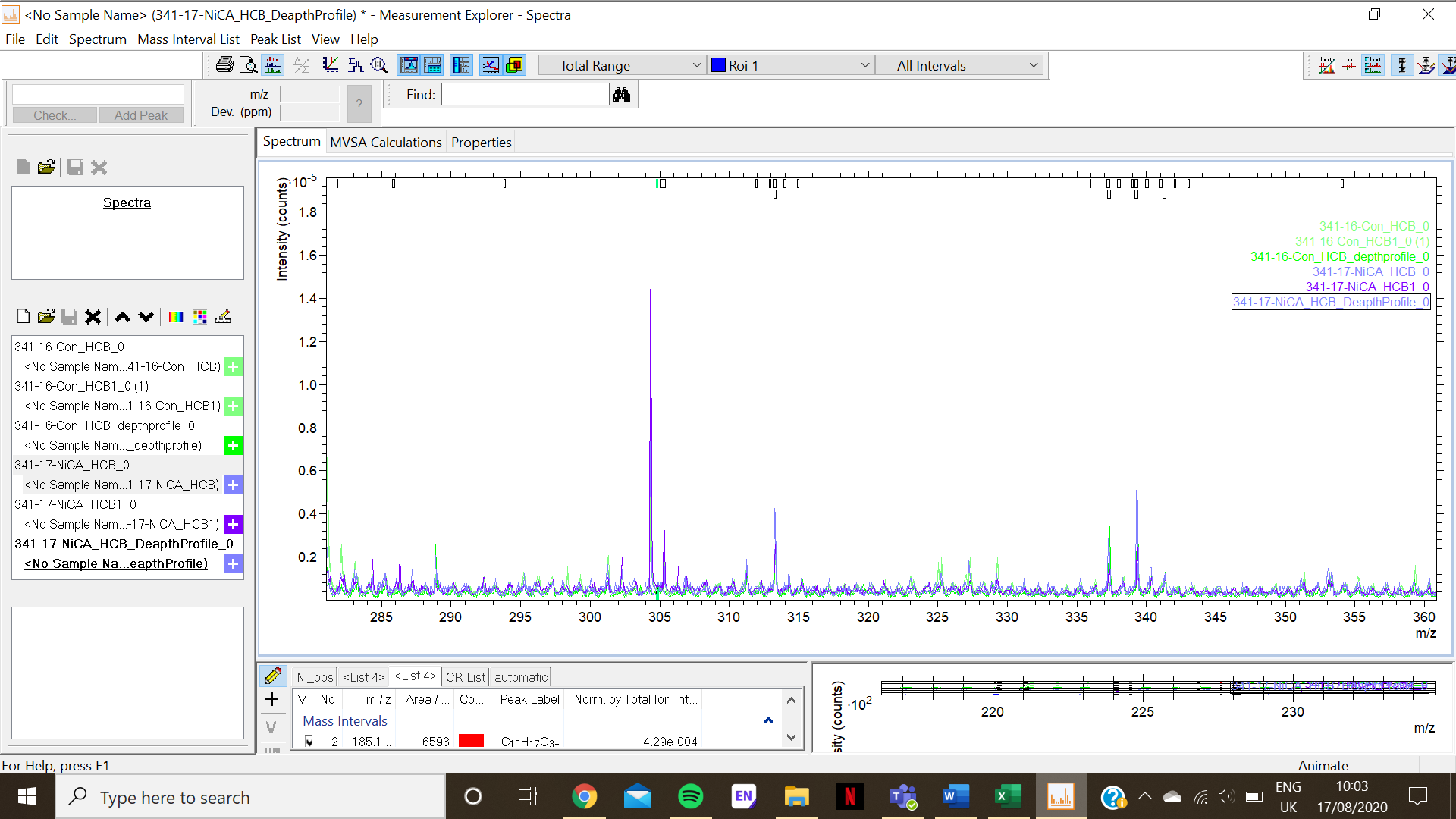
Figure S3: Ion Profile: Nickel Treated, Stratum Corneum: MAG

313 m/z C_19_H_37_O_3_

337 m/z C_21_H_37_O_3_

339 m/z C_21_H_39_O_3_

341 m/z C_21_H_41_O_3_

#
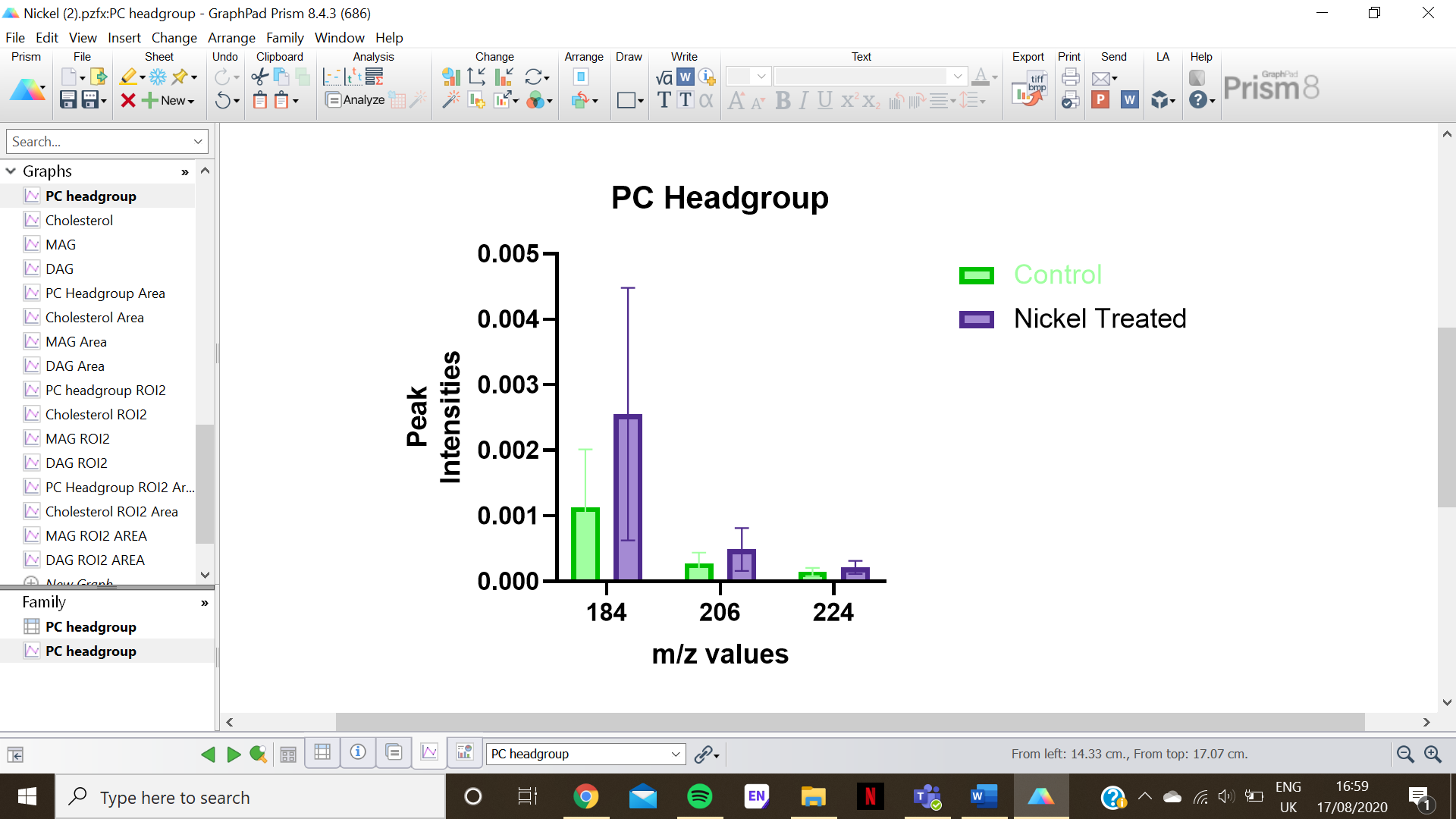

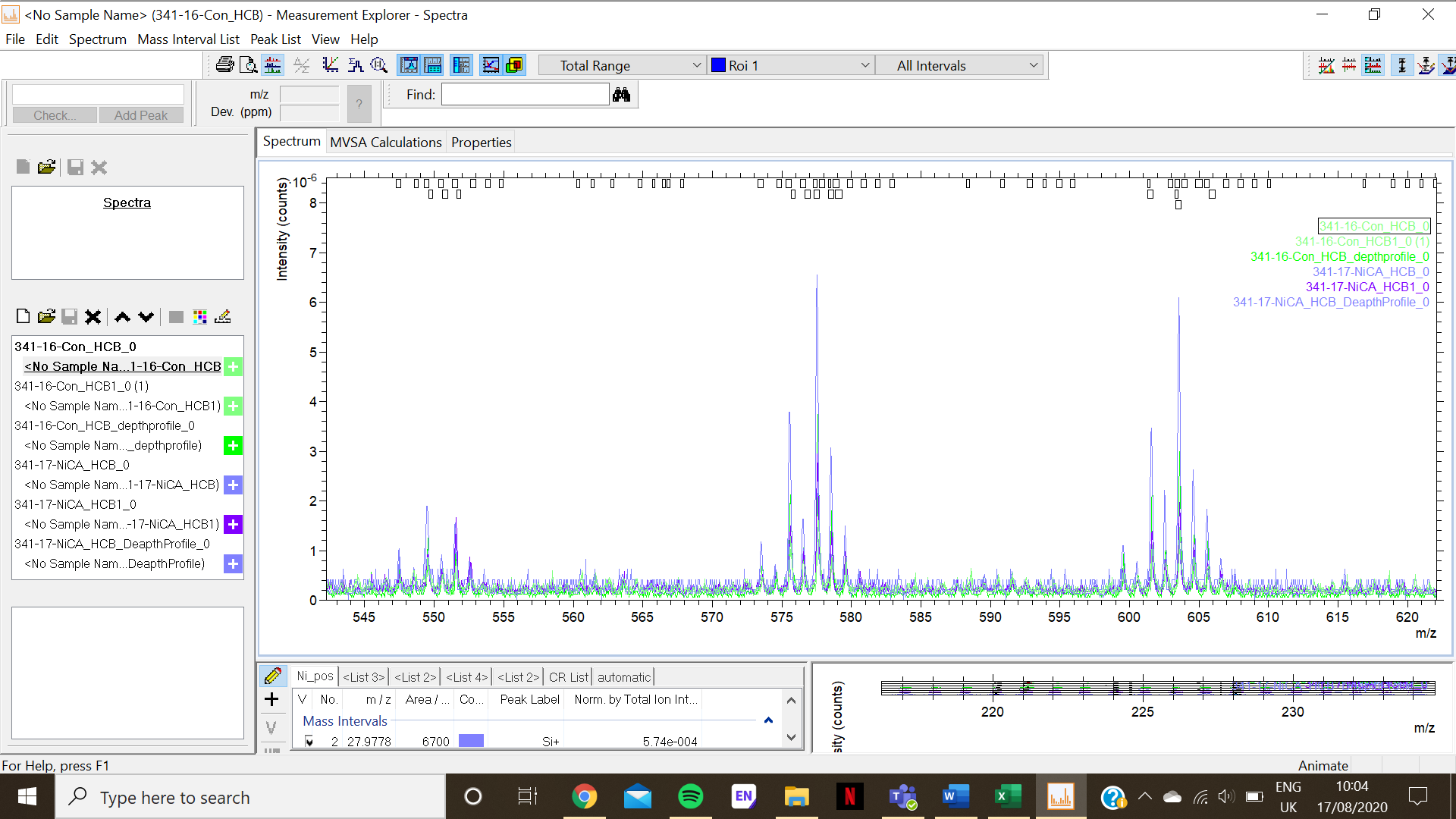
Figure S4: Ion Profile: Nickel Treated, Stratum Corneum: DAG

# Table S2. *m/z* peak area values for nickel-treated skin in viable epidermis, normalised by corrected peak area (positive ion mode)

|  | ***Control Samples*** | | | ***Nickel-Treated Samples*** | | |
| --- | --- | --- | --- | --- | --- | --- |
| ***m/z*** | **Control 1** | **Control 2** | **Control 3** | **Nickel 1** | **Nickel 2** | **Nickel3** |
| **PC Headgroup** |  |  |  |  |  |  |
| **184** | 262875 | 165772.8 | 50873.13 | 877708.2 | 628820.9 | 27559.65 |
| **206** | 85519.98 | 49383.36 | 23868.03 | 172752.2 | 139904.8 | 13763.63 |
| **224** | 12042.22 | 6501.71 | 11313.83 | 29110.19 | 25254.64 | 4932.52 |
|  |  |  |  |  |  |  |
| **Cholesterol** |  |  |  |  |  |  |
| **367** | 10659.94 | 5712.56 | 37192.19 | 3919.21 | 5897.99 | 6263.11 |
| **369** | 25667.96 | 13817.45 | 138097.3 | 9616.59 | 12995.64 | 19362.88 |
| **384** | 5433.42 | 3150.33 | 10750.04 | 1741.76 | 2391.58 | 1423.03 |
| **385** | 6126.35 | 3433.44 | 12027.99 | 2147.66 | 2999.92 | 1566.4 |
|  |  |  |  |  |  |  |
| **Vitamin E** |  |  |  |  |  |  |
| **430** | 4283.06 | 3387.11 | 7012.47 | 1951.83 | 3784.53 | 1597.14 |
|  |  |  |  |  |  |  |
| **MAG** |  |  |  |  |  |  |
| **313** | 7093.92 | 4415.53 | 29000.99 | 1975.1 | 3770.82 | 3350.01 |
| **337** | 7952.12 | 5182 | 37637.25 | 1739.06 | 2827.7 | 2416.67 |
| **339** | 8238.63 | 5466.08 | 46266.88 | 1909.38 | 3850.88 | 4000.45 |
| **341** | 5114.01 | 3162.3 | 12829.1 | 1415.69 | 2366.15 | 1514.51 |
|  |  |  |  |  |  |  |
| **DAG** |  |  |  |  |  |  |
| **547** | 1949.48 | 1135.98 | 5213.64 | 931.71 | 1417.03 | 807.28 |
| **549** | 3045.29 | 1667.9 | 10815.17 | 2079.49 | 2429.96 | 1436.65 |
| **551** | 2056.75 | 1279.24 | 8410.26 | 1351.49 | 1773.69 | 1083.43 |
| **573** | 1640.09 | 983.74 | 6274.58 | 637.32 | 1045.59 | 850.32 |
| **575** | 3092.44 | 1703.48 | 22722.25 | 957.52 | 2016.36 | 2566.5 |
| **577** | 4348.28 | 2501.85 | 37988.93 | 1412.98 | 3129.86 | 4379.8 |
| **579** | 1353.23 | 702.73 | 1984.92 | 818.5 | 1023.84 | 540.38 |
| **601** | 2593.69 | 1443.02 | 20521.8 | 821.36 | 1661.95 | 2295.2 |
| **603** | 3454.75 | 2019.86 | 32234.99 | 1144.63 | 2292.63 | 3698.84 |
| **605** | 1745.23 | 1031.88 | 9958.71 | 723.41 | 1252.8 | 1208.55 |

#
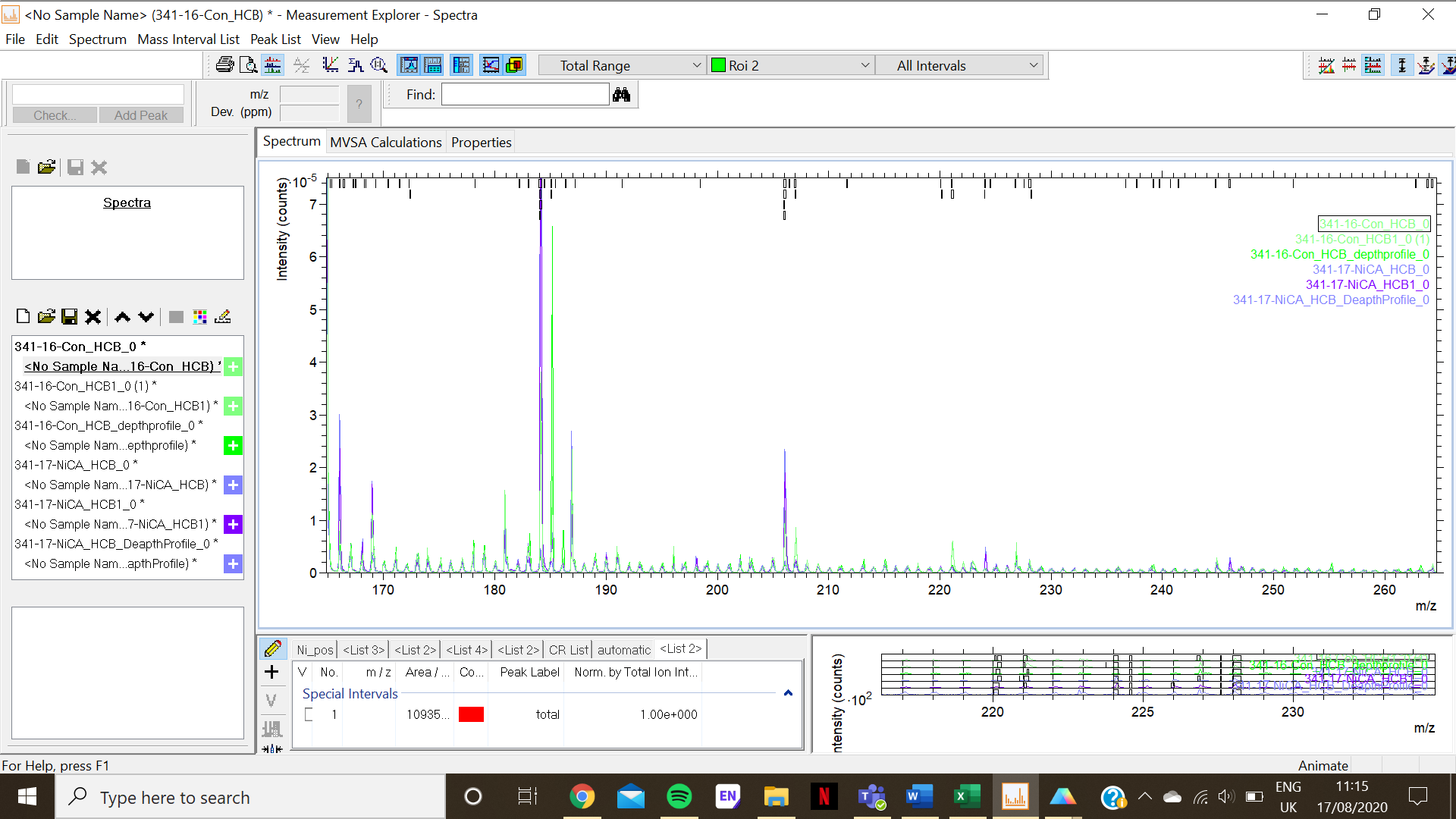

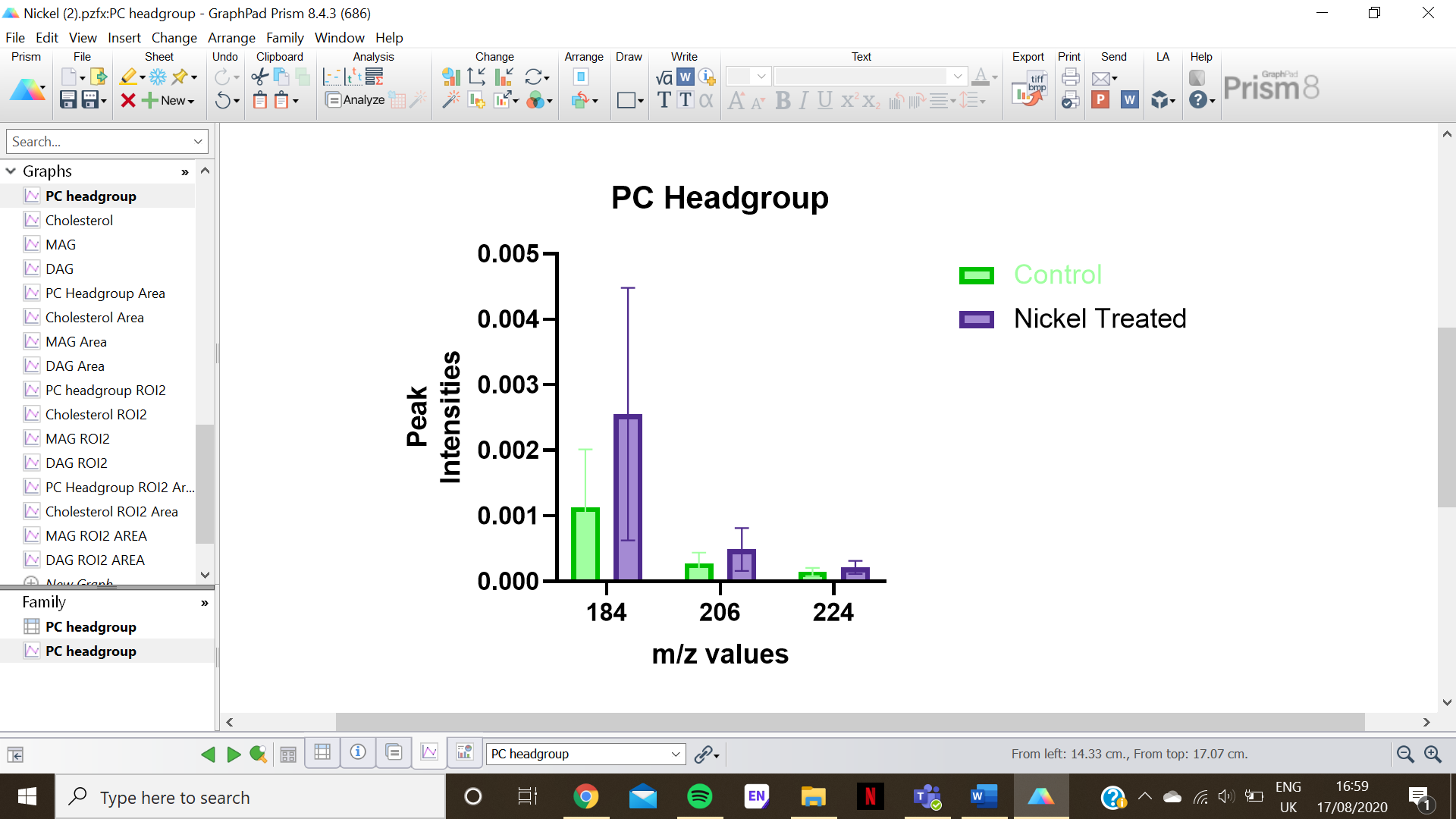
Figure S5: Ion Profile: Nickel Treated, Viable Epidermis: PC Headgroup

206 m/z C_5_H_14_NPO_4_Na

224 m/z C_8_H_19_NPO_4_Na

184 m/z C_5_H_15_NPO_4_

#
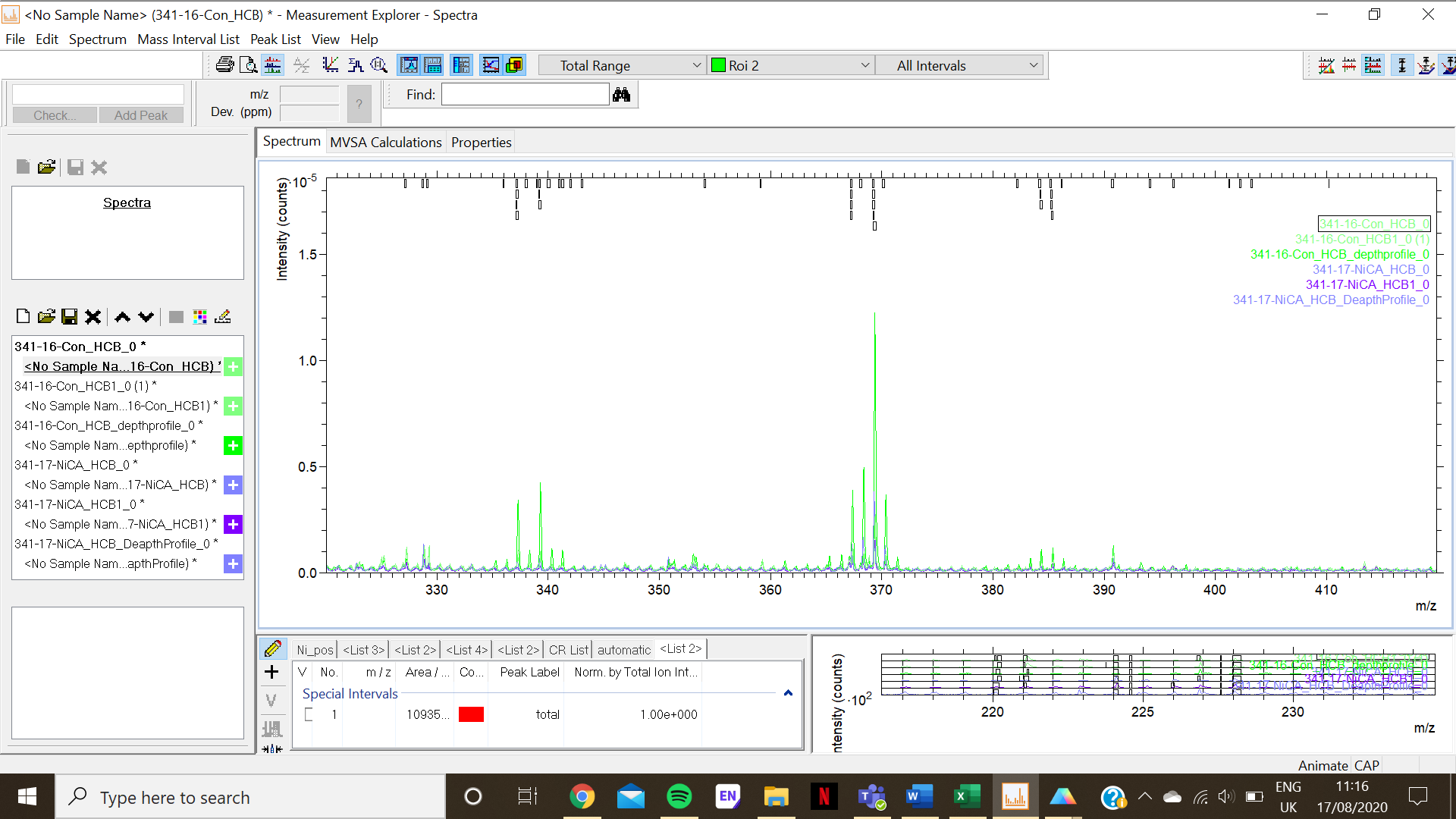

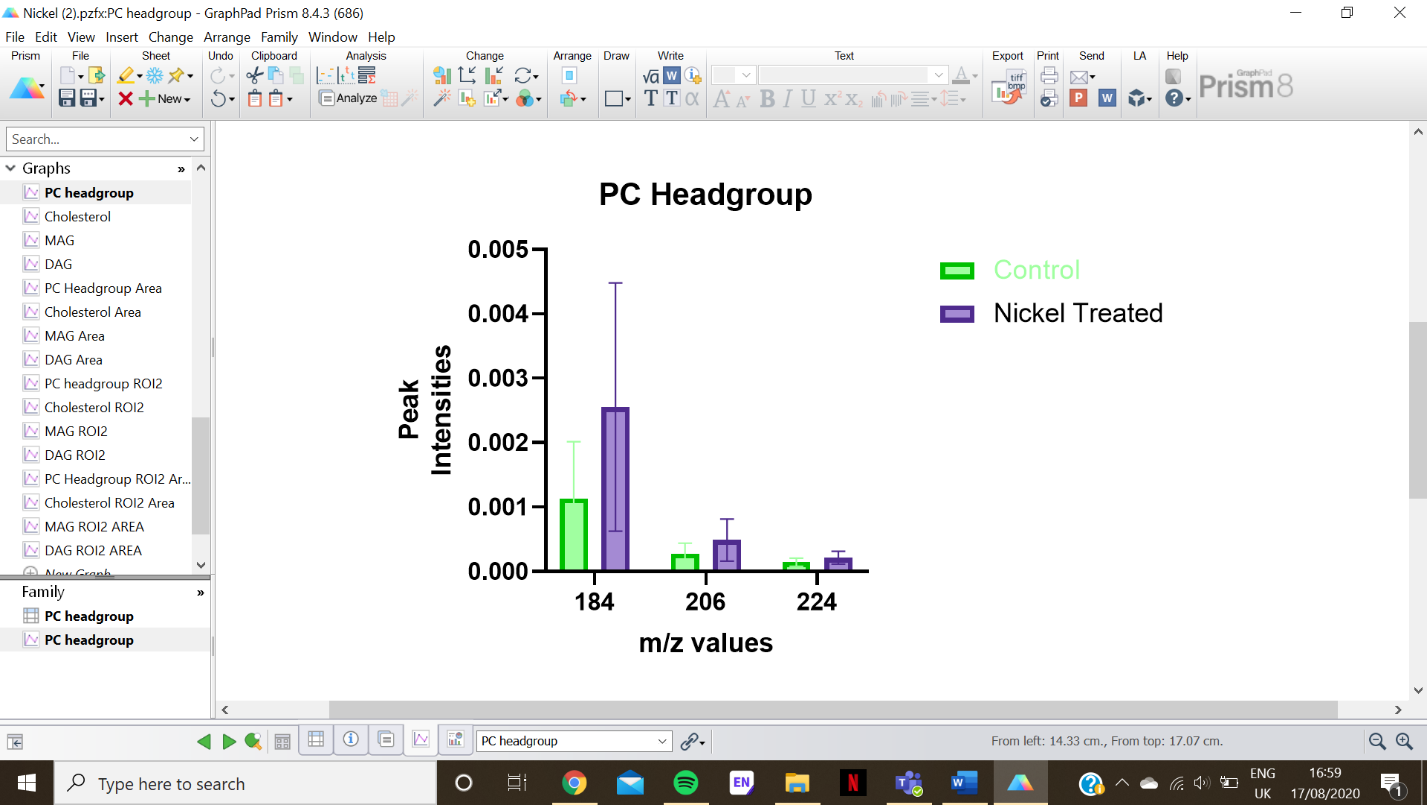
Figure S6: Ion Profile: Nickel Treated, Viable Epidermis: Cholesterol

367 m/z C_27_H_43_

385 m/z C_27_H_45_O

384 m/z C_27_H_44_O

369 m/z C_27_H_45_

#
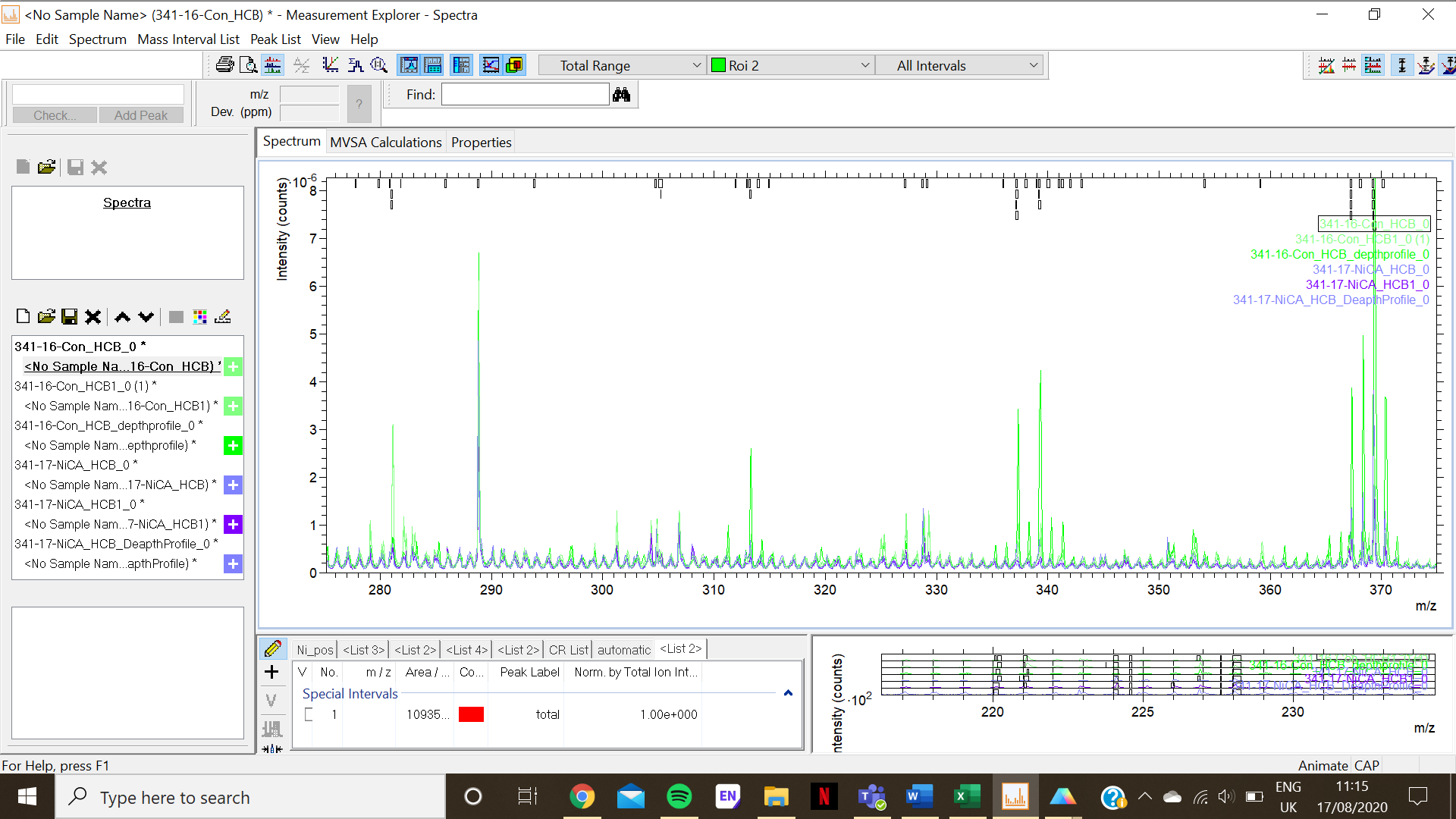

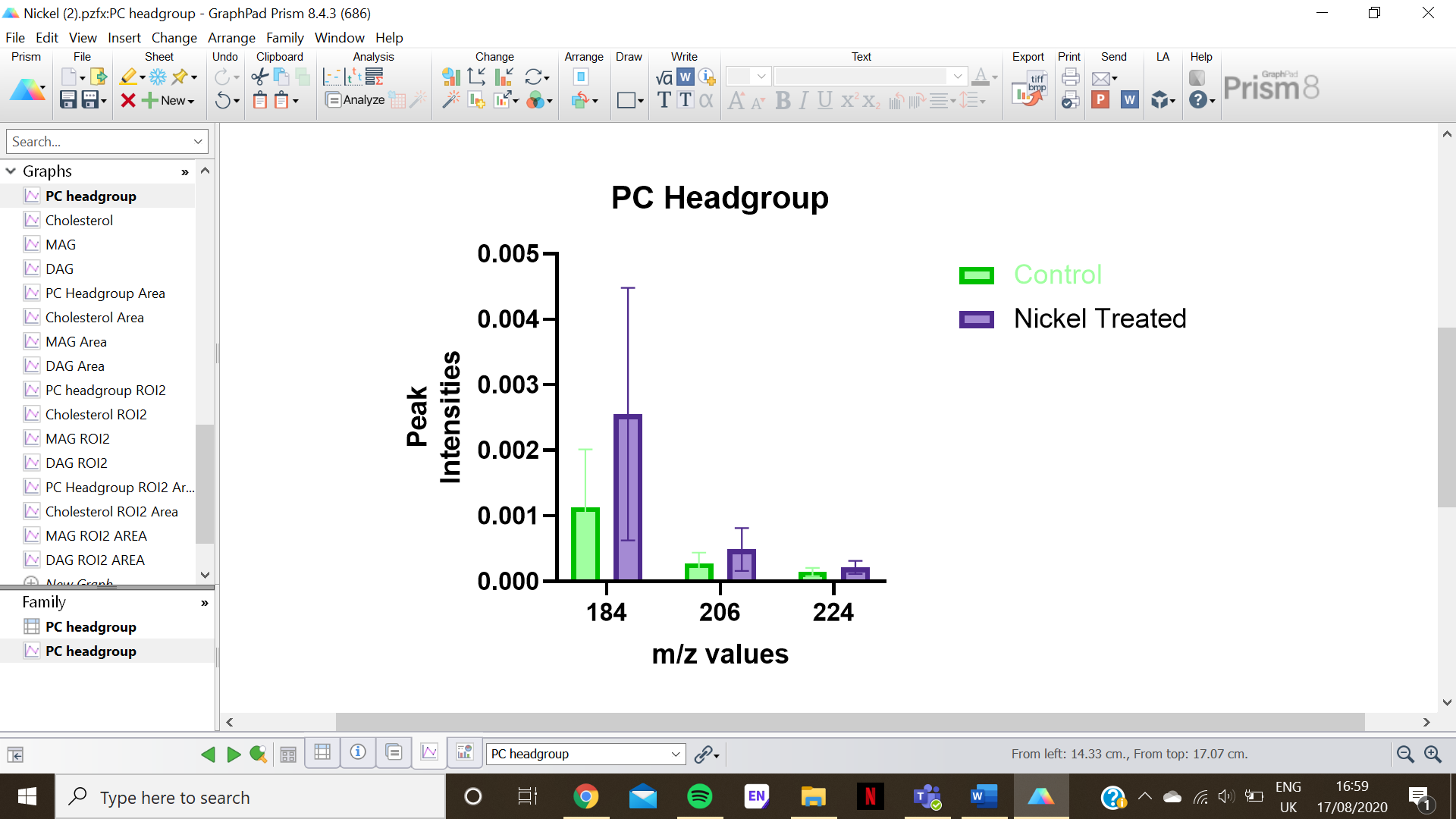
Figure S7: Ion Profile: Nickel Treated, Viable Epidermis: MAGs

313 m/z C_19_H_37_O_3_

341 m/z C_21_H_41_O_3_

339 m/z C_21_H_39_O_3_

337 m/z C_21_H_37_O_3_

]

# Figure S8: Ion Profile: Nickel Treated, Viable Epidermis: DAGs


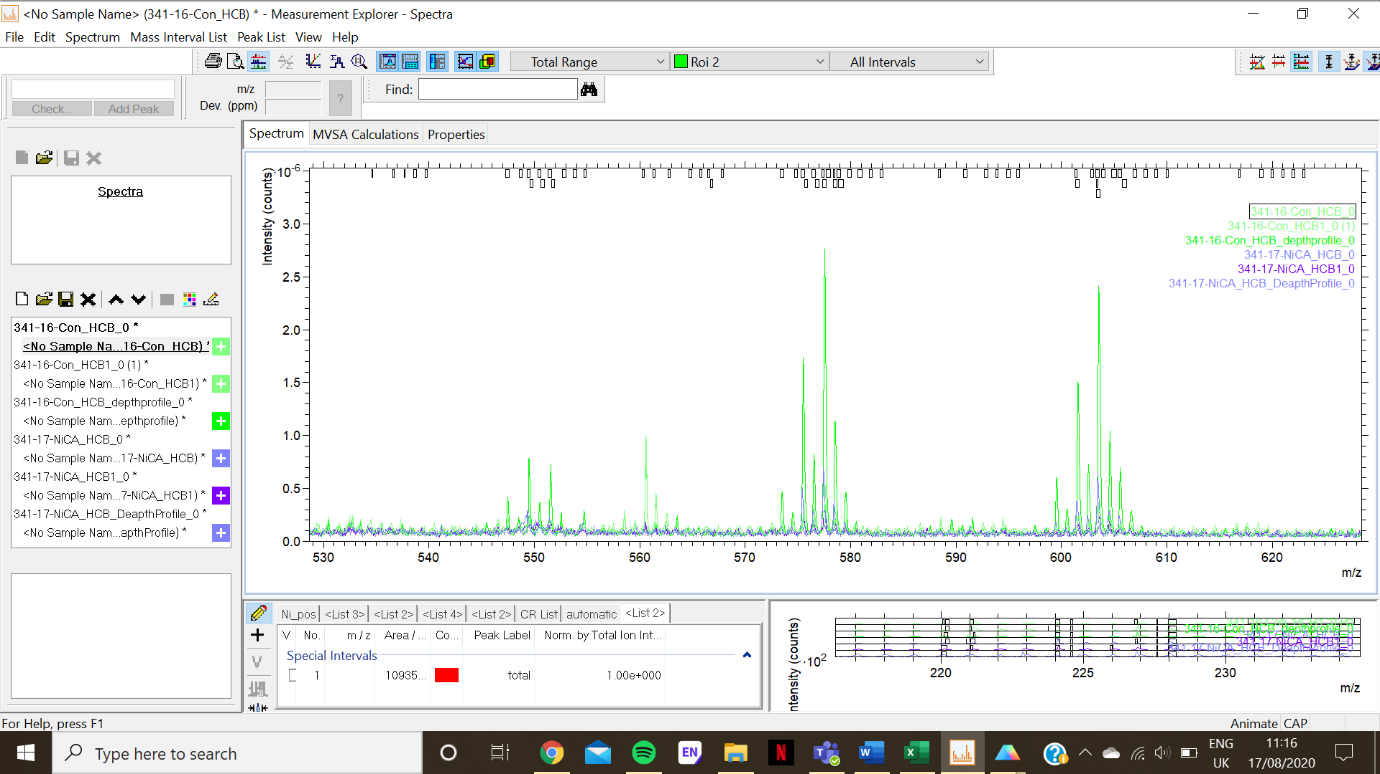


# Table S4. *m/z* peak area values for chromium-treated skin in stratum corneum, normalised by corrected peak area (positive ion mode)

|  | ***Control Samples*** | | | ***Chromium-Treated Samples*** | | |
| --- | --- | --- | --- | --- | --- | --- |
| ***m/z*** | **Control 1** | **Control 2** | **Control 3** | **Cr 1** | **Cr 2** | **Cr 3** |
| **PC Headgroup** |  |  |  |  |  |  |
| **184** | 71119.7 | 683769 | 31516.5 | 8562.28 | 14108.5 | 7552.99 |
| **206** | 4969.37 | 13945 | 1977.56 | 521.41 | 1365.98 | 1429.8 |
| **224** | 4149.65 | 33607 | 1918.52 | 608.46 | 1002.58 | 799.34 |
|  |  |  |  |  |  |  |
| **Cholesterol** |  |  |  |  |  |  |
| **367** | 452.18 | 1339.64 | 170.08 | 212.07 | 1377.91 | 1700.71 |
| **369** | 397.16 | 817.31 | 163.07 | 394.22 | 1573.2 | 2894.81 |
| **384** | 236.08 | 335.11 | 58.02 | 176.06 | 265.08 | 269.05 |
| **385** | 278.12 | 254.06 | 75.03 | 199.06 | 253.07 | 278.06 |
|  |  |  |  |  |  |  |
| **Vitamin E** |  |  |  |  |  |  |
| **430** | 208.05 | 188.03 | 46.01 | 191.06 | 294.08 | 215.03 |
|  |  |  |  |  |  |  |
| **MAG** |  |  |  |  |  |  |
| **313** | 571.31 | 2011.19 | 94.03 | 492.24 | 2345.18 | 2745.76 |
| **337** | 343.14 | 537.15 | 83.03 | 298.12 | 602.26 | 928.29 |
| **339** | 384.16 | 976.33 | 83.03 | 403.23 | 1665.27 | 2785.65 |
| **341** | 451.24 | 855.33 | 92.04 | 555.57 | 1023.57 | 1085.42 |
|  |  |  |  |  |  |  |
| **DAG** |  |  |  |  |  |  |
| **547** | 145.02 | 171.02 | 39.01 | 211.06 | 339.08 | 510.08 |
| **549** | 194.04 | 386.07 | 51.01 | 411.25 | 1074.44 | 1434.46 |
| **551** | 299.08 | 868.36 | 59.01 | 811.14 | 2961.91 | 2510.35 |
| **573** | 126.02 | 126.01 | 31.01 | 206.04 | 271.05 | 460.06 |
| **575** | 142.02 | 283.04 | 33.01 | 577.38 | 495.17 | 1633.54 |
| **577** | 169.03 | 691.15 | 33.01 | 1668.39 | 2312.79 | 3621.52 |
| **579** | 168.03 | 232.07 | 35.01 | 5325.93 | 216.14 | 312.18 |
| **601** | 73.01 | 193.02 | 33.01 | 485.26 | 601.17 | 1166.29 |
| **603** | 115.02 | 316.04 | 21 | 1234.17 | 1315.67 | 2773.49 |
| **605** | 105.01 | 235.03 | 33.01 | 2566.63 | 788.34 | 1088.41 |

# Figure S9: Ion Profile: Chromium Treated, Stratum Corneum: PC Headgroup


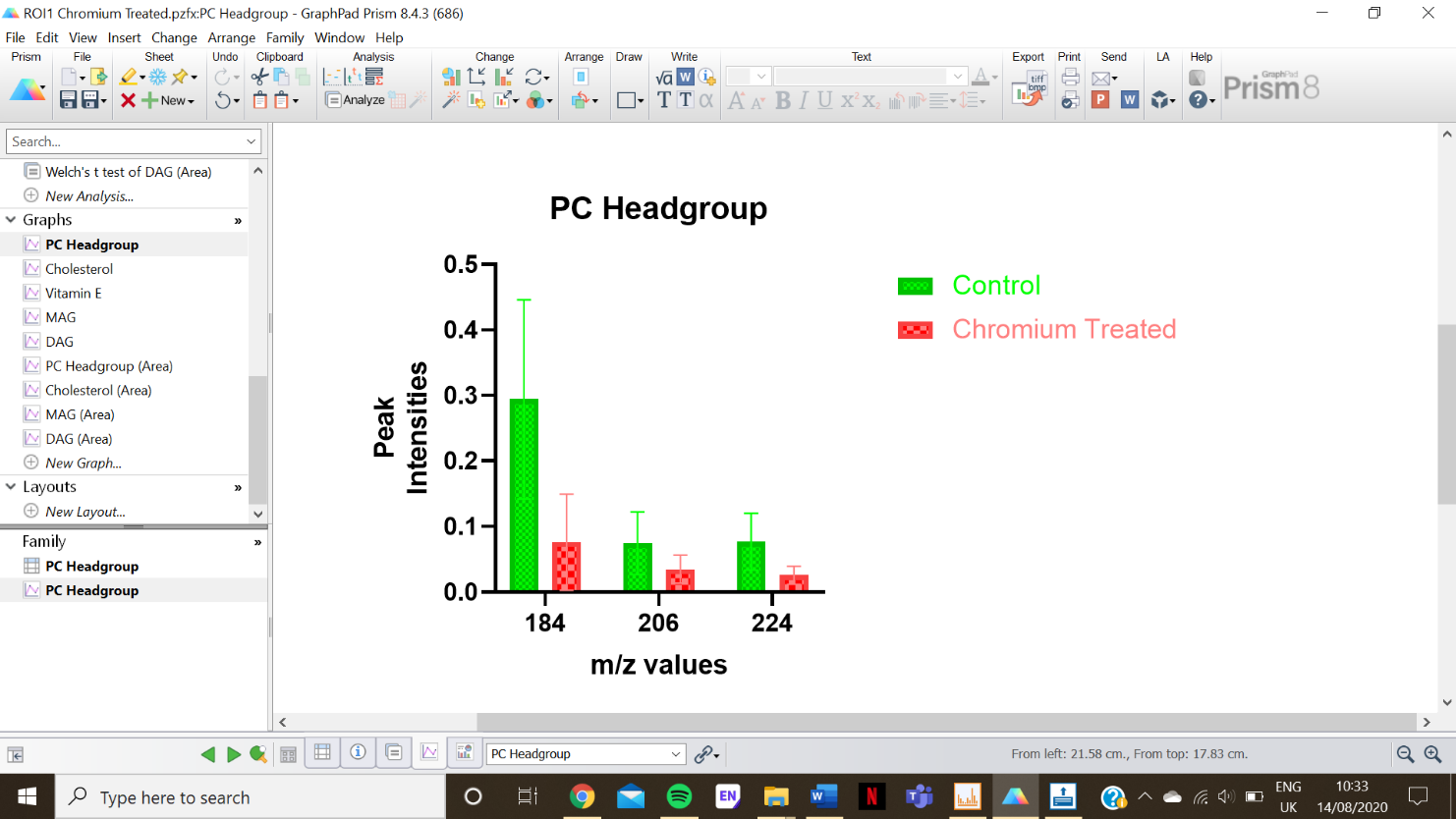


224 m/z C_8_H_19_NPO_4_Na

206 m/z C_5_H_14_NPO_4_Na

184 m/z C_5_H_15_NPO_4_


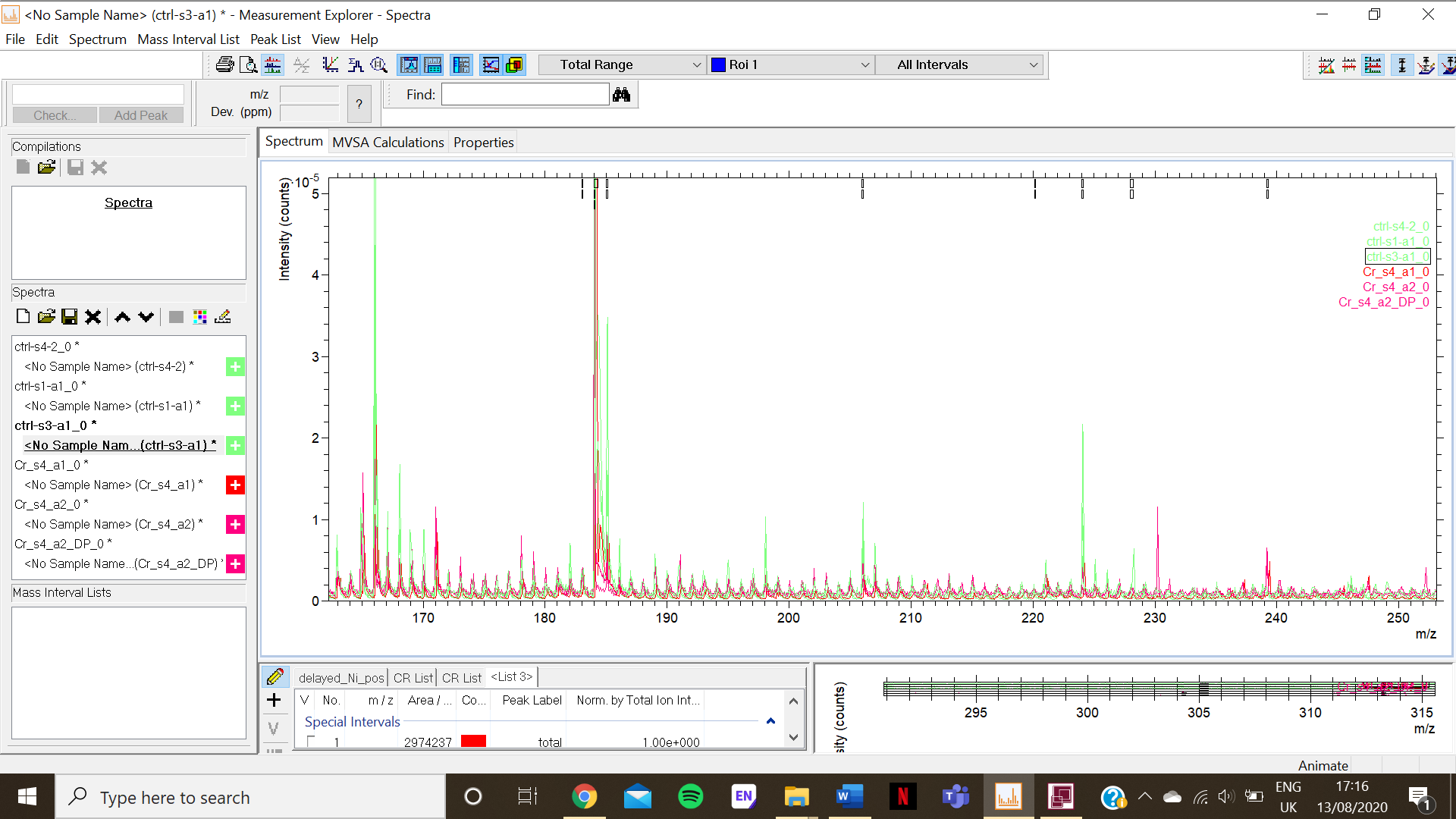


# Figure S10: Ion Profile: Chromium Treated, Stratum Corneum: Cholesterol


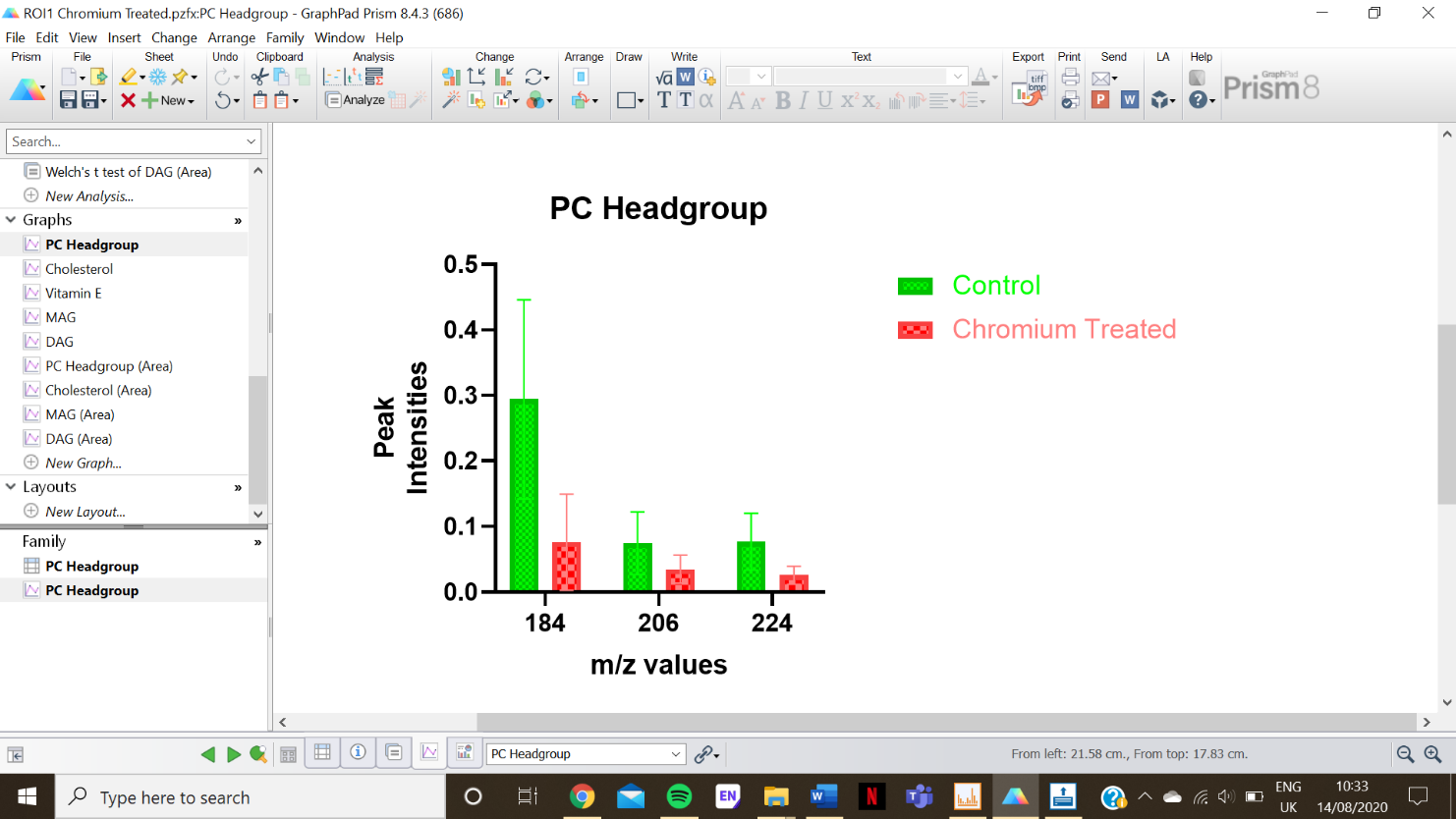

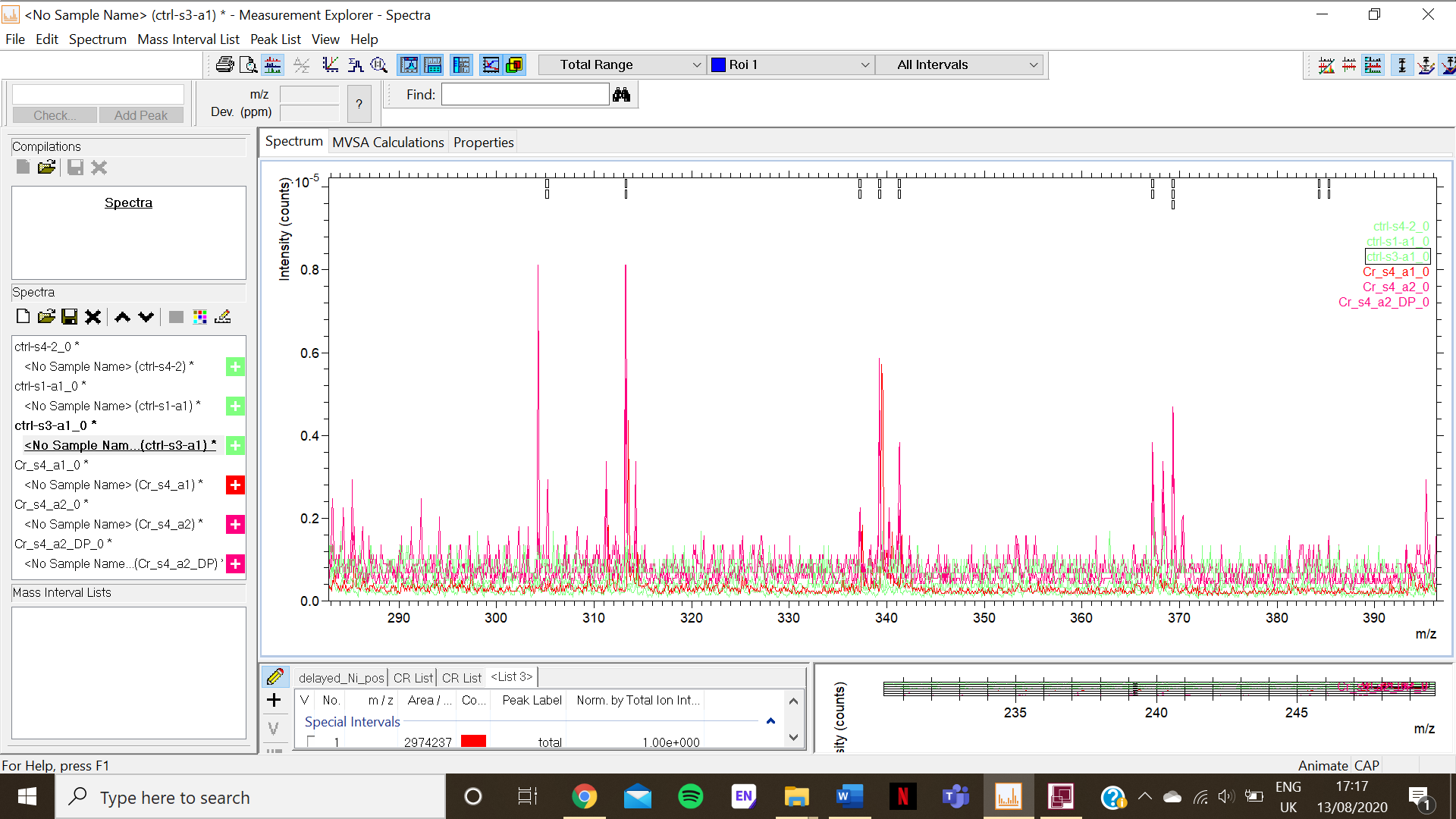


384 m/z C_27_H_44_O

385 m/z C_27_H_45_O

369 m/z C_27_H_45_

367 m/z C_27_H_43_

#
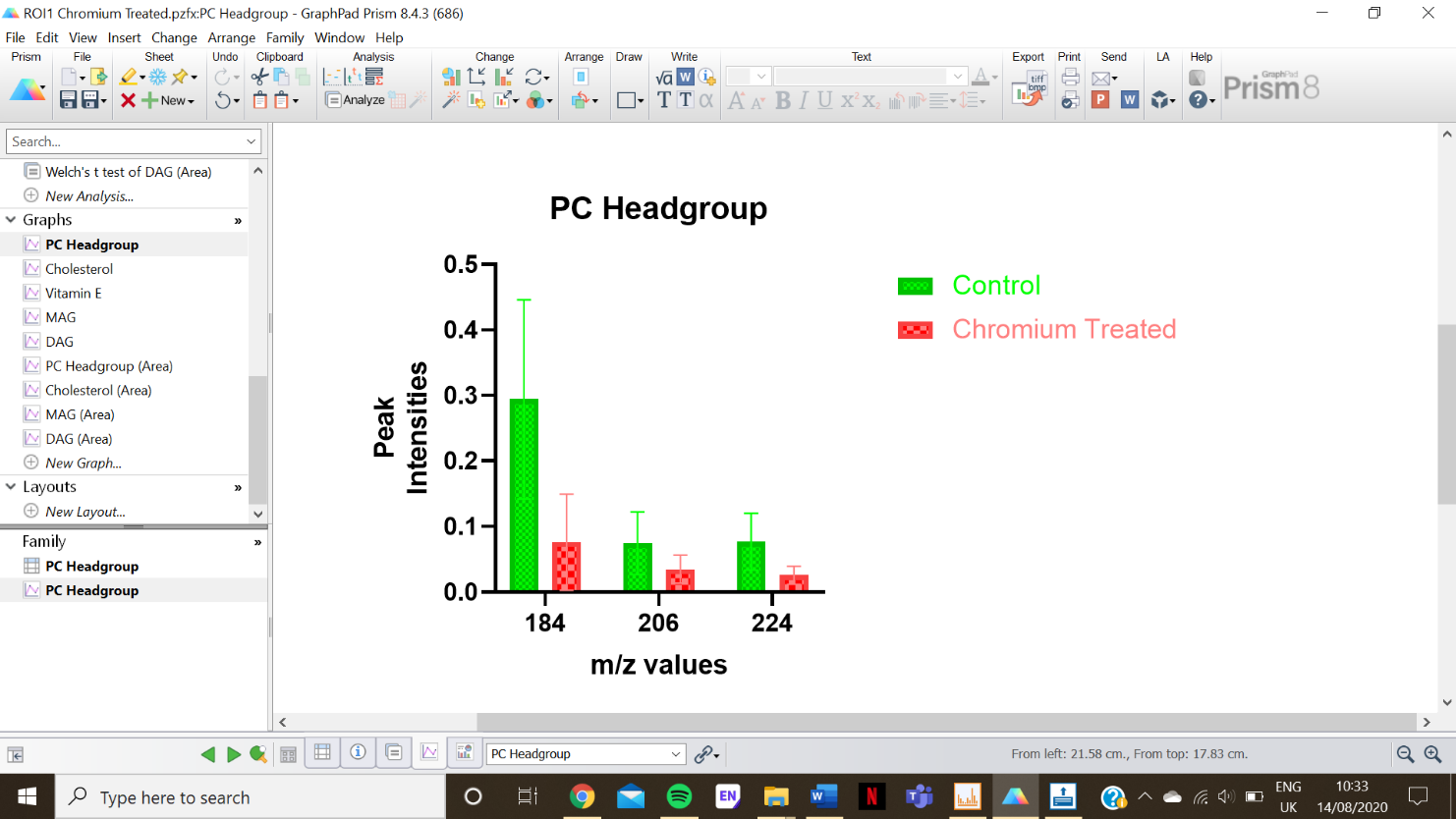

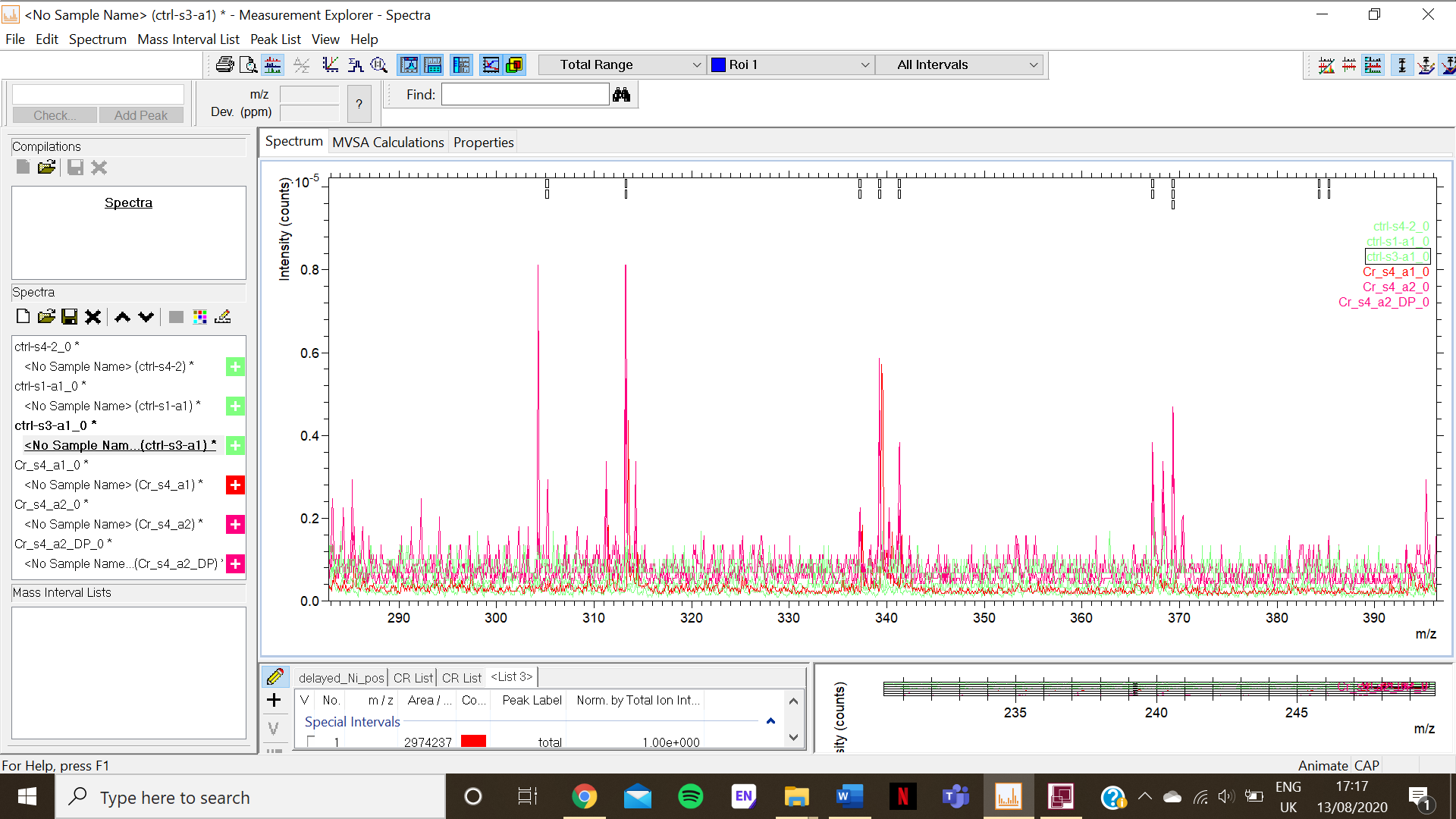
Figure S11: Ion Profile: Chromium Treated, Stratum Corneum: MAG

313 m/z C_19_H_37_O_3_

337 m/z C_21_H_37_O_3_

339 m/z C_21_H_39_O_3_

341 m/z C_21_H_41_O_3_

# Figure S12: Ion Profile: Chromium Treated, Stratum Corneum: DAG


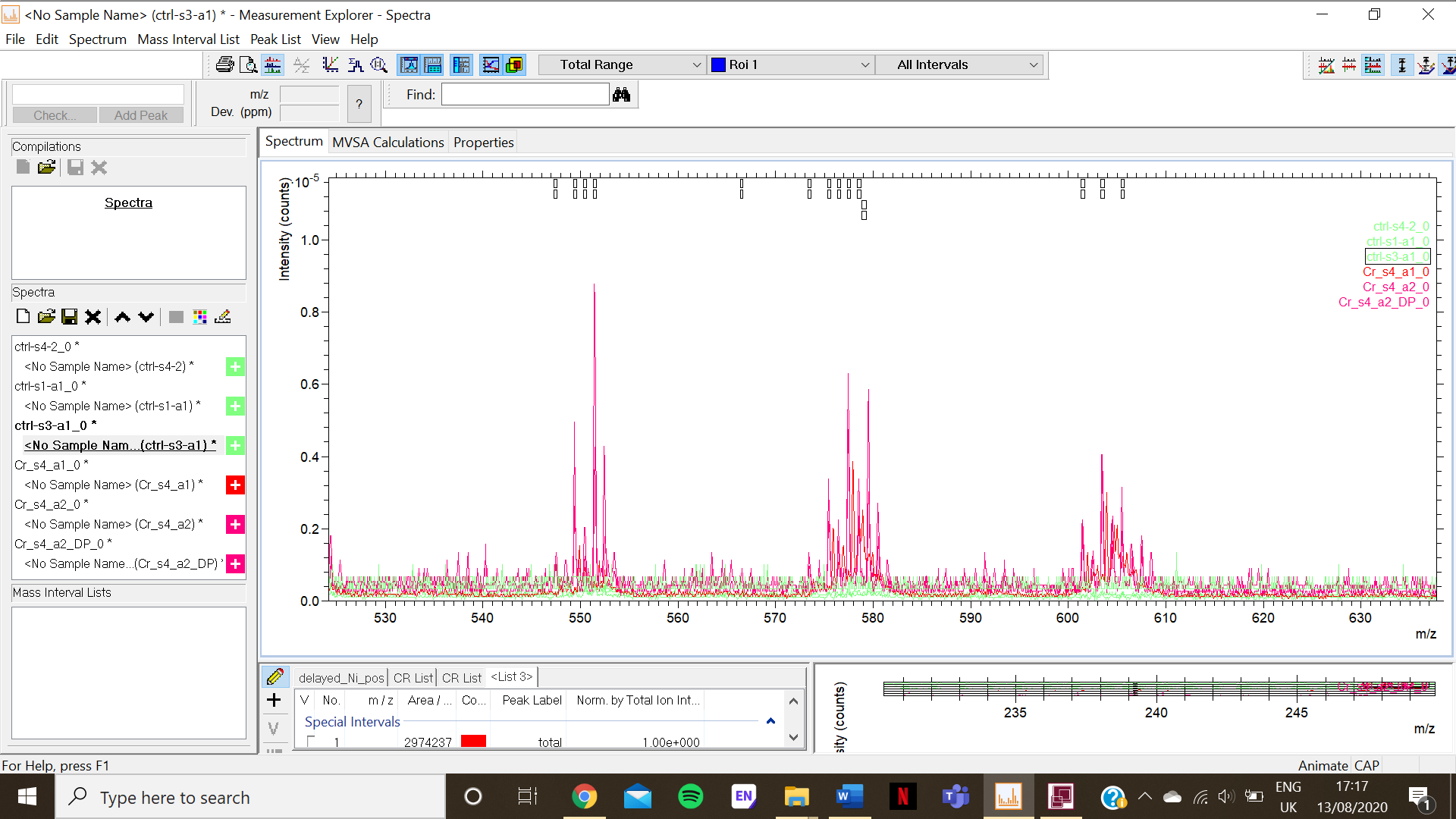


# Table S5. *m/z* peak area values for chromium-treated skin in viable epidermis, normalised by corrected peak area (positive ion mode)

|  | ***Control Samples*** | | | ***Chromium-Treated Samples*** | | |
| --- | --- | --- | --- | --- | --- | --- |
| ***m/z*** | **Control 1** | **Control 2** | **Control 3** | **Cr 1** | **Cr 2** | **Cr 3** |
| **PC Headgroup** |  |  |  |  |  |  |
| **184** | 998055.4 | 801932.7 | 1524940 | 53511.6 | 103846.1 | 100702.7 |
| **206** | 22031.63 | 13916.41 | 36715.88 | 1231.83 | 3682.73 | 6089.79 |
| **224** | 53478.57 | 37117.42 | 74926.26 | 1825.23 | 3782.82 | 4692.39 |
|  |  |  |  |  |  |  |
| **Cholesterol** |  |  |  |  |  |  |
| **367** | 2096.73 | 915.24 | 1023.24 | 718.23 | 831.21 | 1646.32 |
| **369** | 2007.63 | 876.21 | 1009.22 | 1974.18 | 1093.32 | 2263.53 |
| **384** | 685.13 | 272.04 | 436.07 | 595.19 | 316.05 | 574.07 |
| **385** | 841.18 | 428.07 | 504.1 | 601.16 | 310.05 | 632.09 |
|  |  |  |  |  |  |  |
| **Vitamin E** |  |  |  |  |  |  |
| **430** | 657.11 | 299.04 | 420.05 | 675.2 | 234.03 | 477.05 |
|  |  |  |  |  |  |  |
| **MAG** |  |  |  |  |  |  |
| **313** | 1525.51 | 827.22 | 864.2 | 2558.3 | 439.09 | 1212.27 |
| **337** | 1145.31 | 513.11 | 721.14 | 1150.47 | 262.05 | 876.15 |
| **339** | 1146.3 | 533.11 | 565.1 | 1954.32 | 450.09 | 1486.3 |
| **341** | 1282.37 | 623.15 | 978.26 | 2355.95 | 279.06 | 765.14 |
|  |  |  |  |  |  |  |
| **DAG** |  |  |  |  |  |  |
| **547** | 386.04 | 187.01 | 270.02 | 893.3 | 184.02 | 379.03 |
| **549** | 441.05 | 183.02 | 286.03 | 1800.67 | 236.03 | 594.05 |
| **551** | 760.16 | 389.07 | 524.1 | 3959.09 | 399.07 | 548.05 |
| **573** | 355.03 | 177.01 | 273.02 | 899.26 | 118.01 | 263.01 |
| **575** | 304.02 | 150.01 | 206.01 | 2474.79 | 190.01 | 693.05 |
| **577** | 333.03 | 194.01 | 216.02 | 8384.76 | 372.03 | 1238.14 |
| **579** | 486.07 | 216.03 | 356.05 | 36676.97 | 111.01 | 287.03 |
| **601** | 268.02 | 120.01 | 187.01 | 2285.93 | 145.01 | 534.03 |
| **603** | 237.01 | 141.01 | 163.01 | 6328.65 | 248.02 | 1180.11 |
| **605** | 241.02 | 123.01 | 146.01 | 13904.07 | 174.01 | 491.04 |

# Figure S13: Ion Profile: Chromium Treated, Viable Epidermis: PC Headgroup


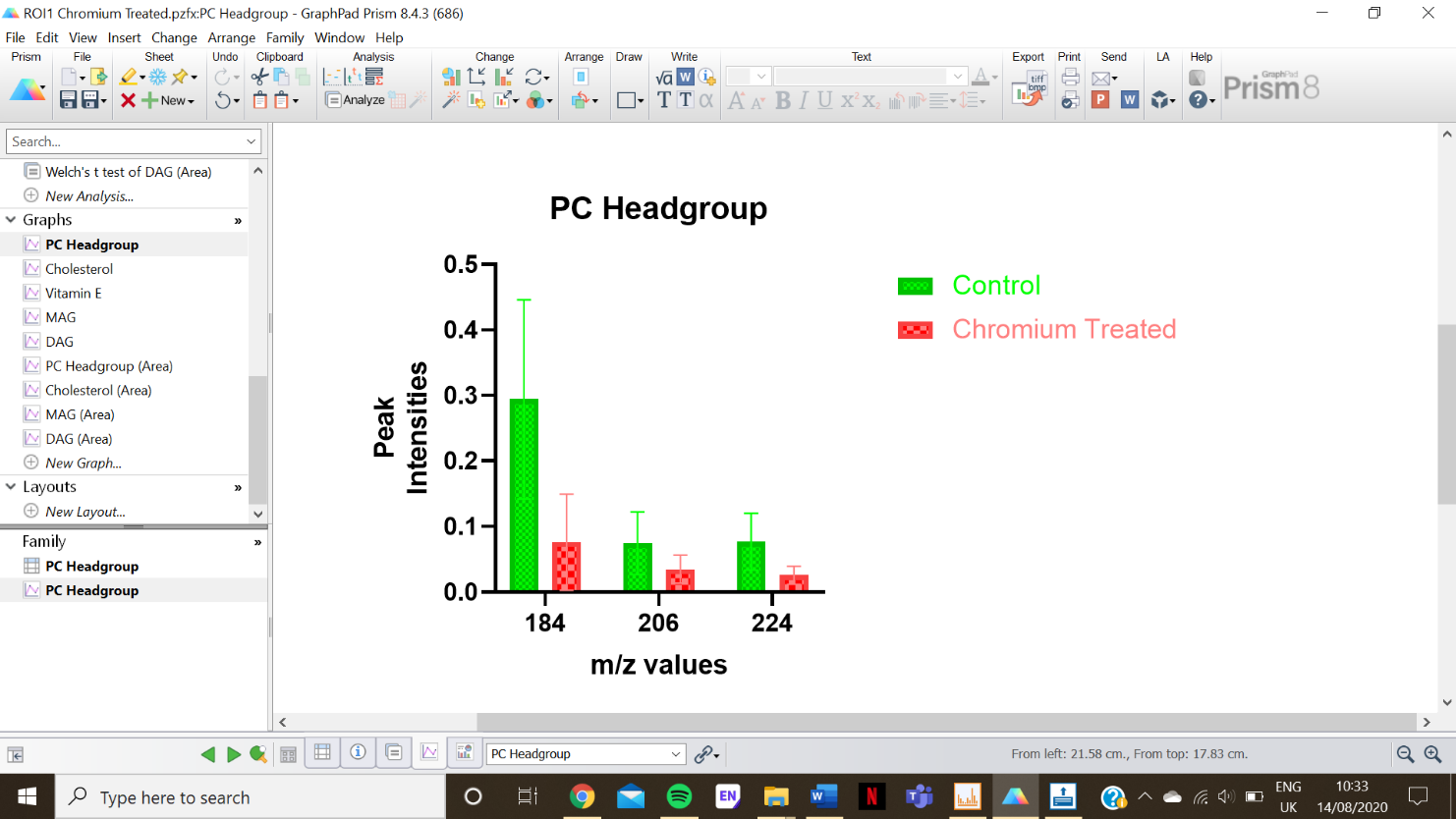

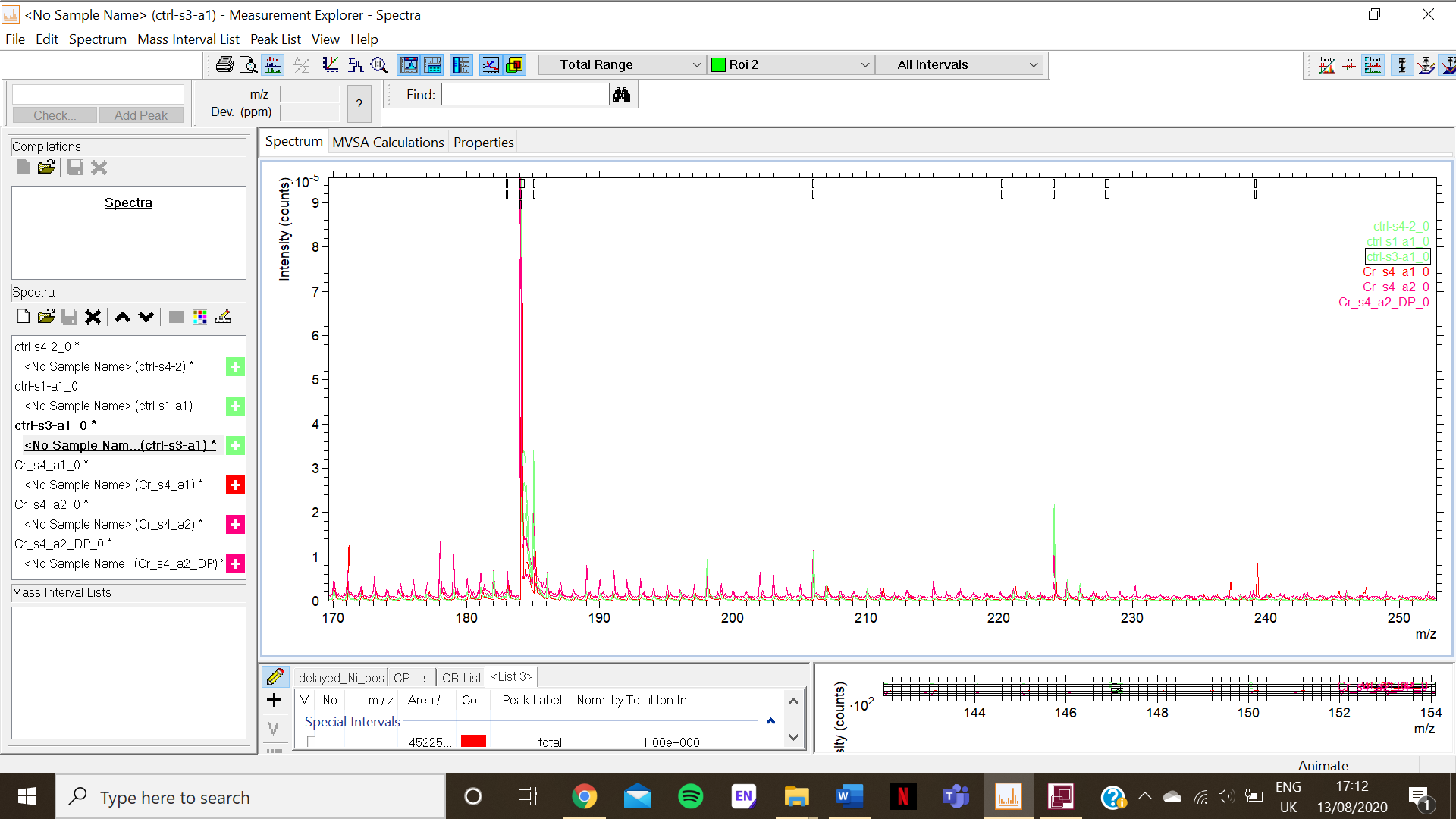


184 m/z C_5_H_15_NPO_4_

206 m/z C_5_H_14_NPO_4_Na

224 m/z C_8_H_19_NPO_4_Na

#
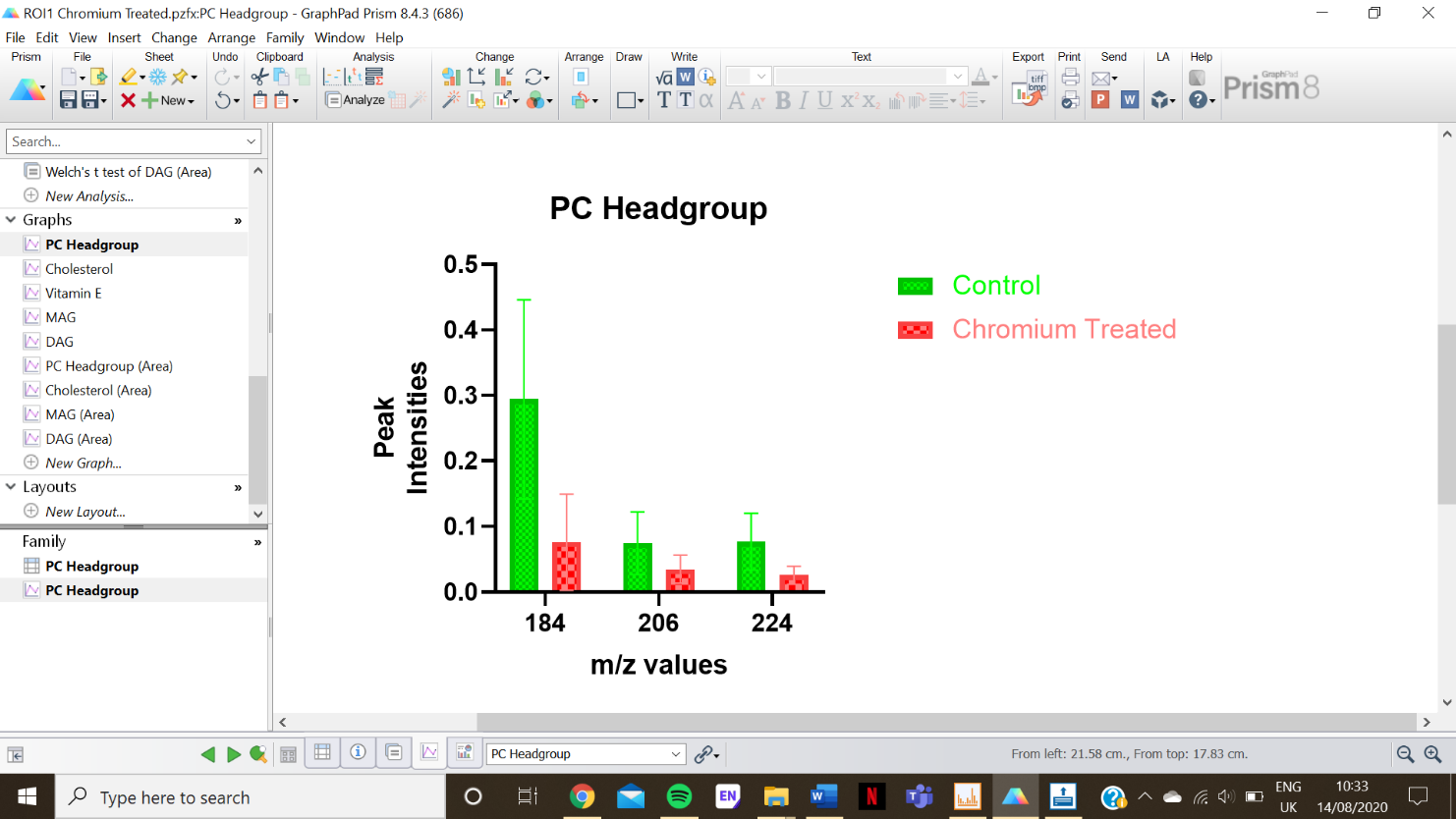

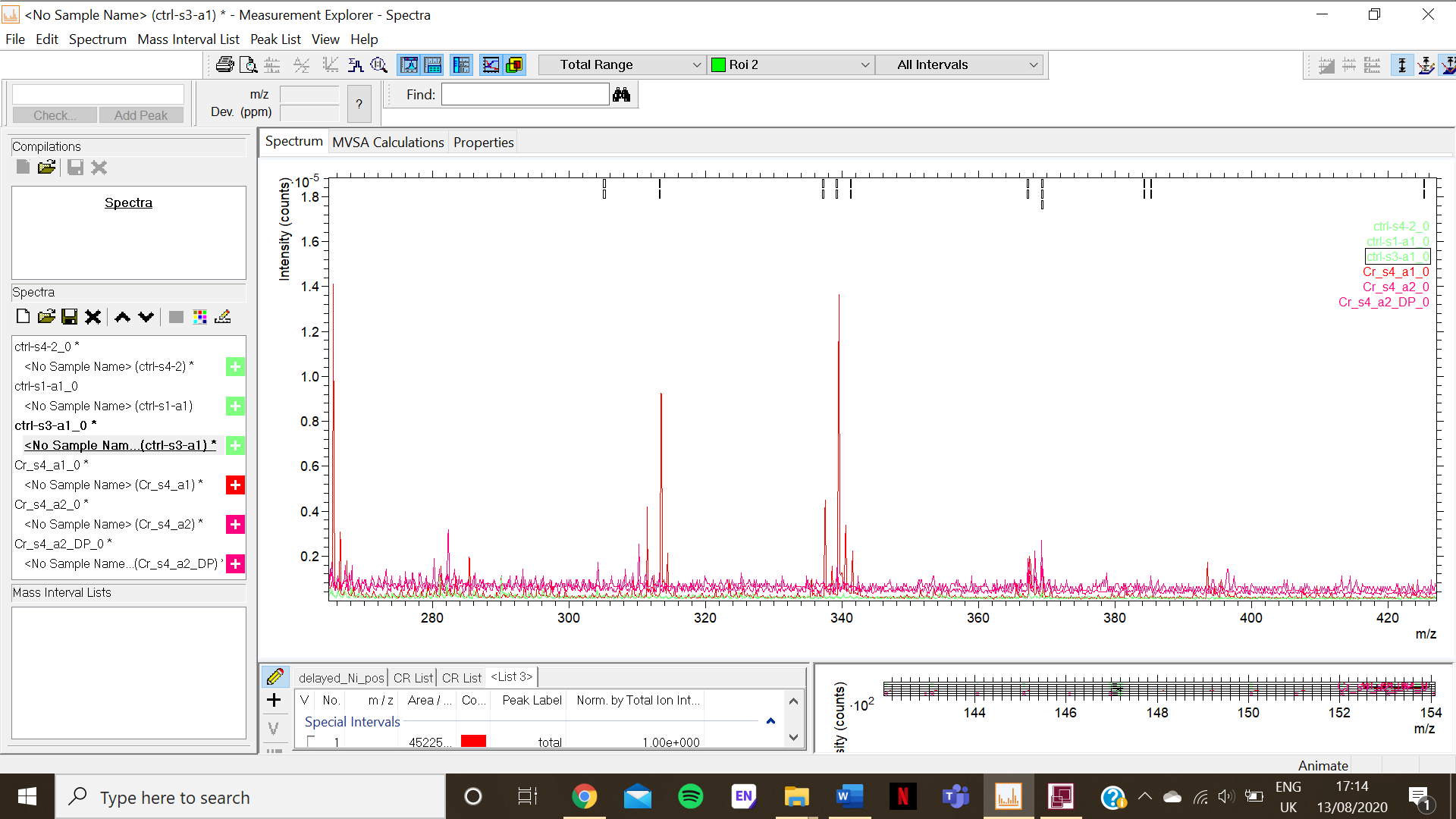
Figure S14: Ion Profile: Chromium Treated, Viable Epidermis: Cholesterol

385 m/z C_27_H_45_O

384 m/z C_27_H_44_O

369 m/z C_27_H_45_

367 m/z C_27_H_43_

#
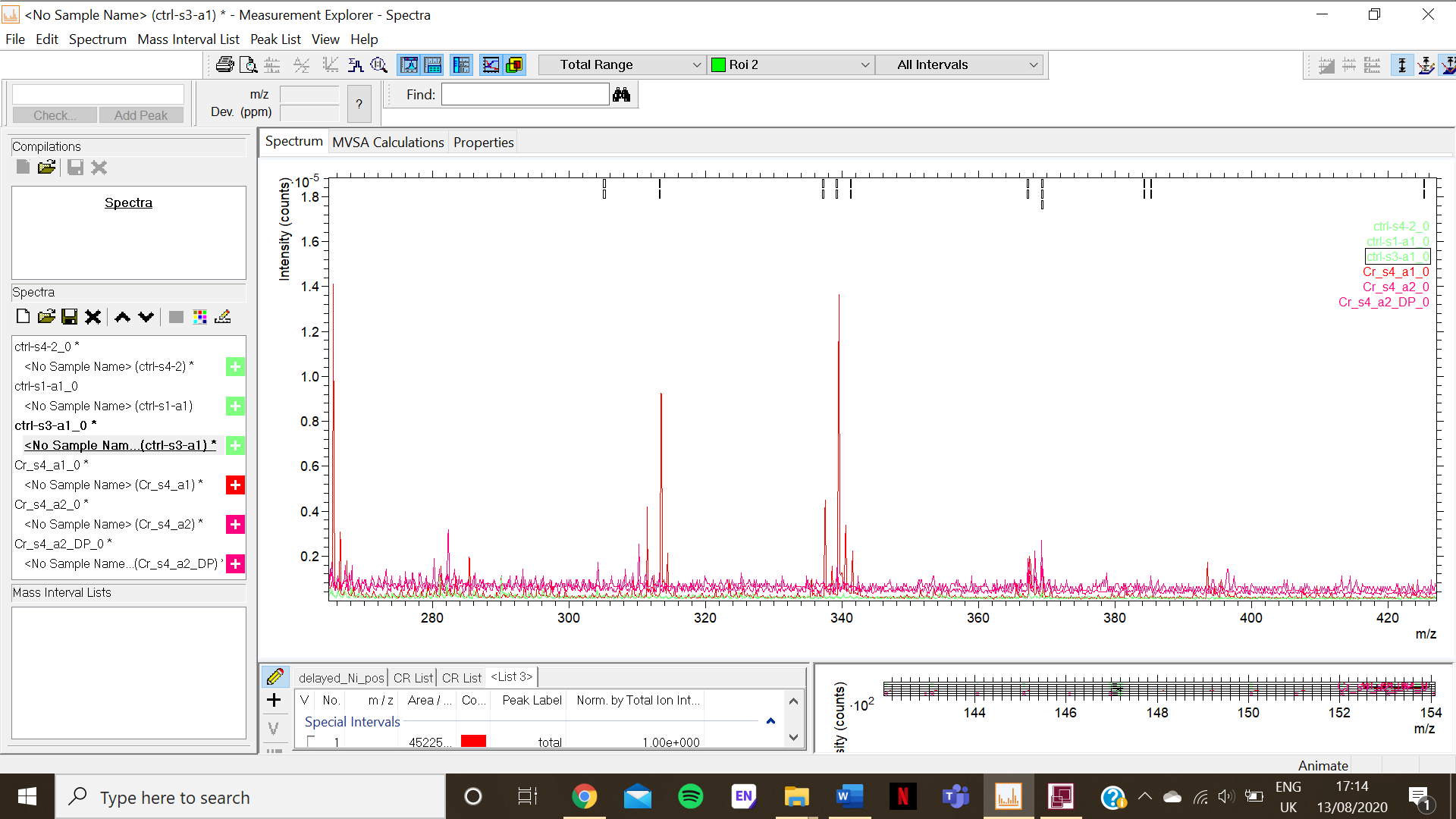

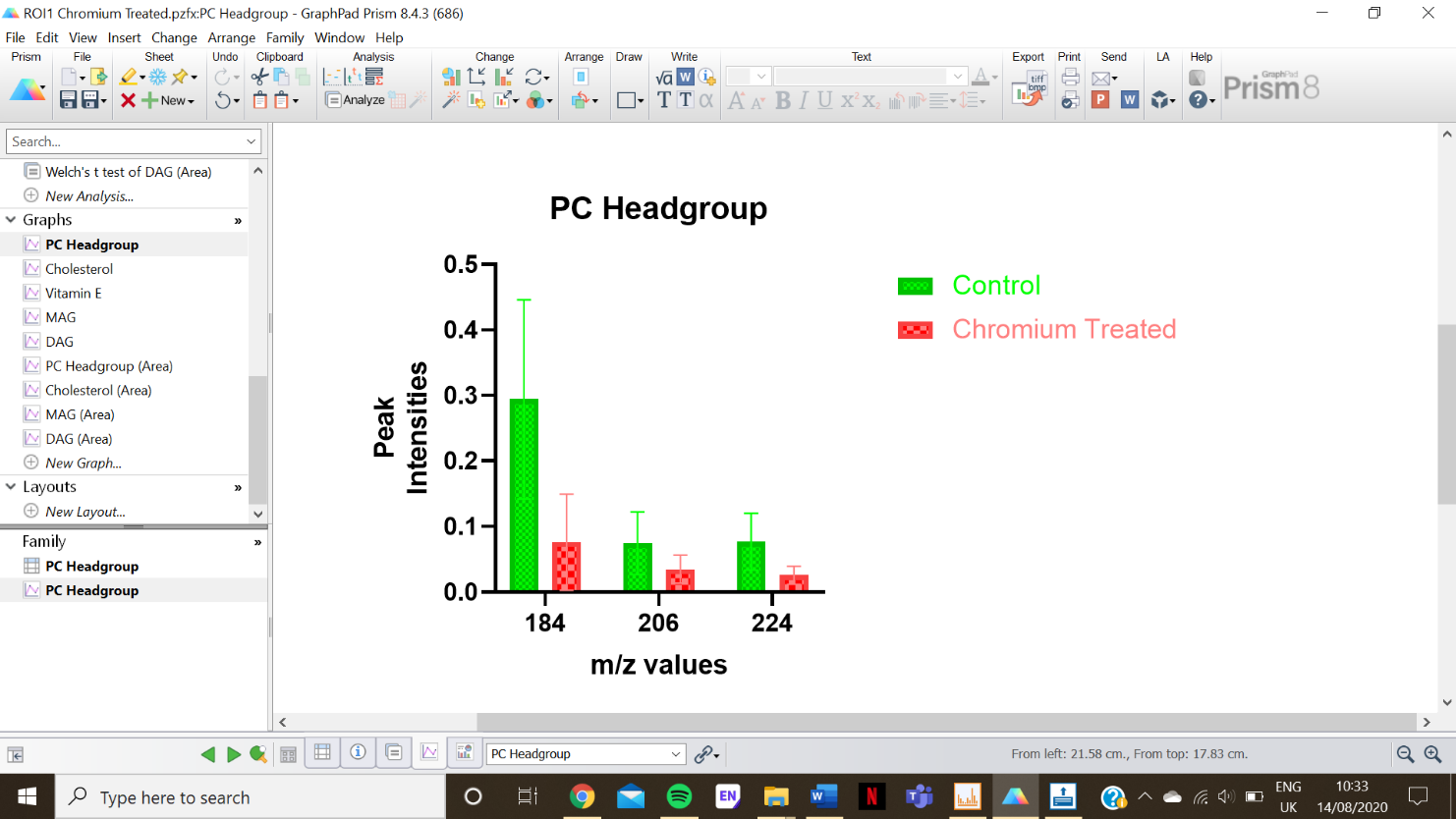
Figure S15: Ion Profile: Chromium Treated, Viable Epidermis: MAG

339 m/z C_21_H_39_O_3_

341 m/z C_21_H_41_O_3_

337 m/z C_21_H_37_O_3_

313 m/z C_19_H_37_O_3_

#
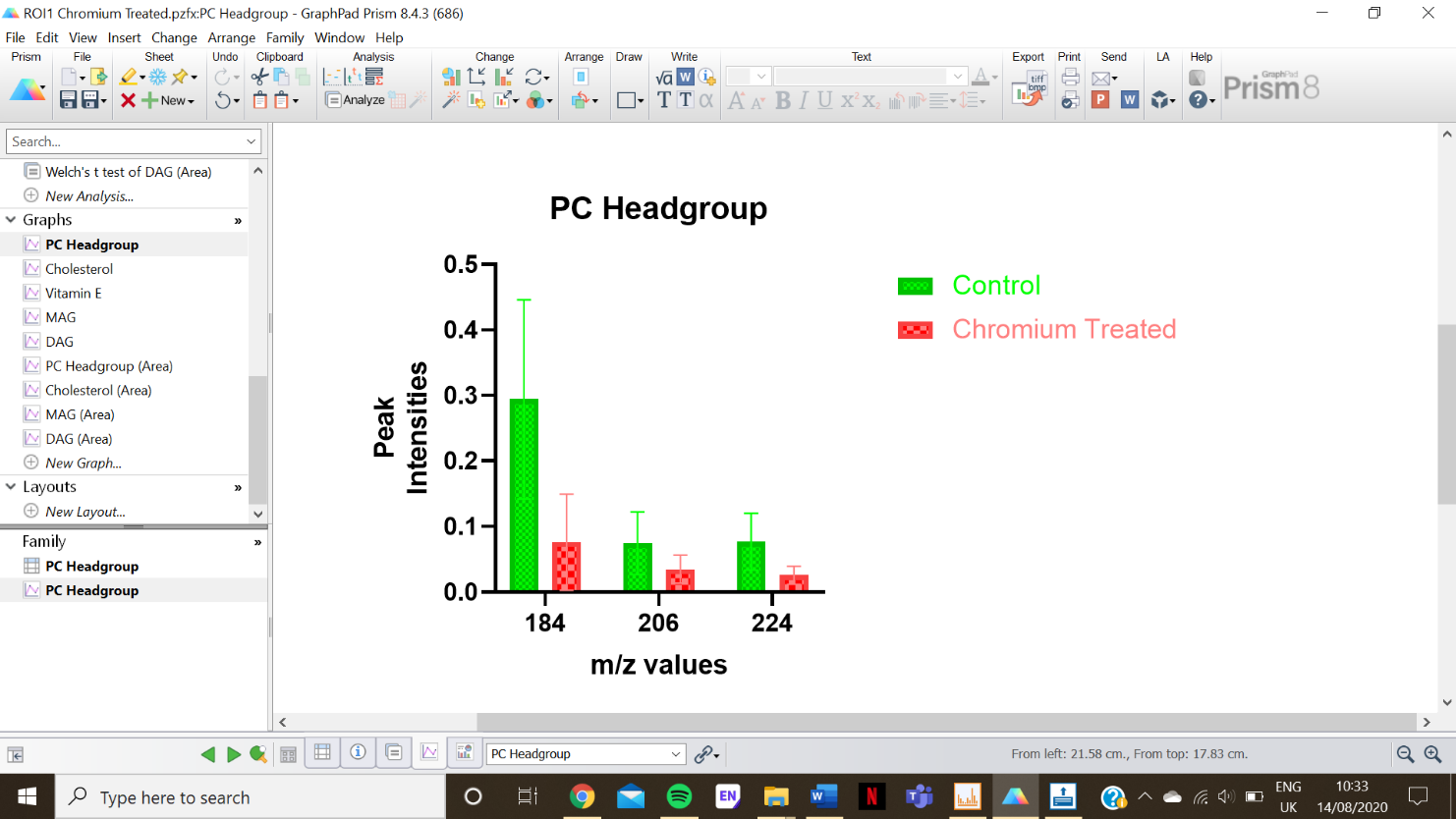

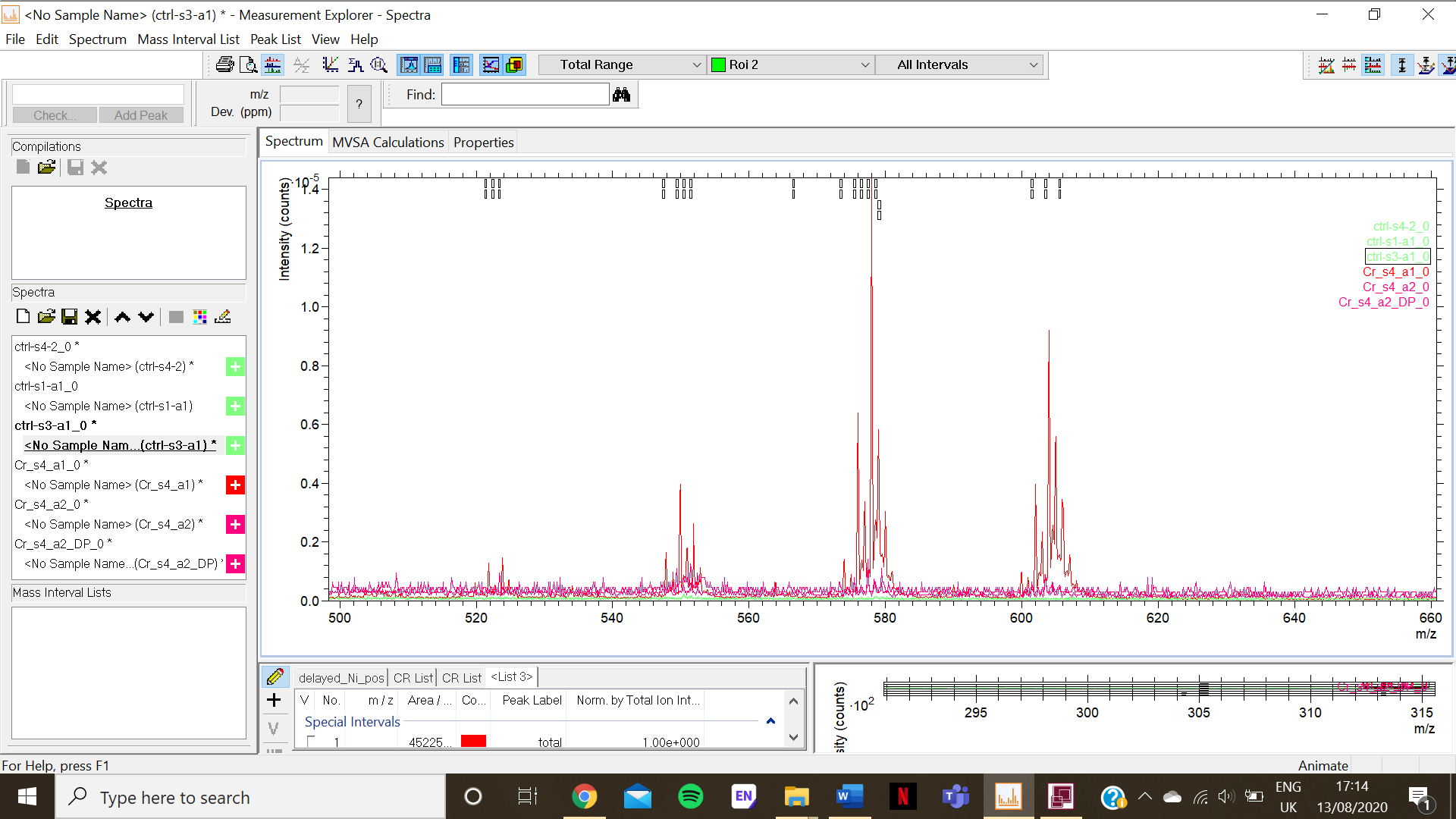
Figure S16: Ion Profile: Chromium Treated, Viable Epidermis: DAG

# Table S6. *m/z* peak area values for cobalt-treated skin in stratum corneum, normalised by corrected peak area (positive ion mode)

|  | ***Control Samples*** | | | ***Cobalt-Treated Samples*** | | |
| --- | --- | --- | --- | --- | --- | --- |
| ***m/z*** | **Control 1** | **Control 2** | **Control 3** | **Cobalt 1** | **Cobalt2** | **Cobalt 3** |
| **PC Headgroup** |  |  |  |  |  |  |
| **184** | 61447.84 | 688531.7 | 31587.72 | 75777.04 | 68090.47 | 369.64 |
| **206** | 6301.85 | 14132.26 | 1976.57 | 4394.83 | 1724.5 | 302.54 |
| **224** | 4738.08 | 33708.96 | 1915.53 | 2272.33 | 2530.42 | 341.67 |
|  |  |  |  |  |  |  |
| **Cholesterol** |  |  |  |  |  |  |
| **367** | 692.17 | 1372.68 | 177.08 | 1735.02 | 248.11 | 348.25 |
| **369** | 683.18 | 780.3 | 161.07 | 701.56 | 247.12 | 725.91 |
| **384** | 423.1 | 306.1 | 58.02 | 778.75 | 57.02 | 1273.77 |
| **385** | 473.13 | 241.05 | 76.03 | 544.41 | 72.03 | 705.86 |
|  |  |  |  |  |  |  |
| **Vitamin E** |  |  |  |  |  |  |
| **430** | 442.08 | 185.03 | 46.01 | 200.07 | 64.01 | 443.22 |
|  |  |  |  |  |  |  |
| **MAG** |  |  |  |  |  |  |
| **313** | 870.3 | 1996.21 | 88.03 | 4389.54 | 492.39 | 346.39 |
| **337** | 686.21 | 518.15 | 81.03 | 927.99 | 134.06 | 293.24 |
| **339** | 666.2 | 976.35 | 83.03 | 2274.36 | 252.14 | 346.4 |
| **341** | 721.24 | 837.33 | 93.04 | 1741.34 | 263.16 | 506.9 |
|  |  |  |  |  |  |  |
| **DAG** |  |  |  |  |  |  |
| **547** | 264.03 | 547.4359 | 35.01 | 740.39 | 47.01 | 209.1 |
| **549** | 303.03 | 549.4437 | 50.01 | 2494.29 | 126.04 | 306.35 |
| **551** | 317.04 | 551.4687 | 56.01 | 7341.06 | 420.45 | 461.23 |
| **573** | 203.02 | 573.4917 | 31.01 | 429.15 | 50.01 | 176.06 |
| **575** | 211.02 | 575.4502 | 32.01 | 1969.15 | 60.01 | 217.19 |
| **577** | 249.02 | 577.4645 | 33.01 | 5344.45 | 169.08 | 341.2 |
| **579** | 266.02 | 578.5335 | 34.01 | 881.47 | 541.25 | 8864.24 |
| **601** | 186.01 | 579.0997 | 33.01 | 663.32 | 37.01 | 154.07 |
| **603** | 176.02 | 601.4571 | 22 | 1888.99 | 57.01 | 233.38 |
| **605** | 187.02 | 603.4677 | 33.01 | 1577.88 | 58.01 | 389.67 |

# Figure S17: Ion Profile: Cobalt Treated, Stratum Corneum: PC Headgroup


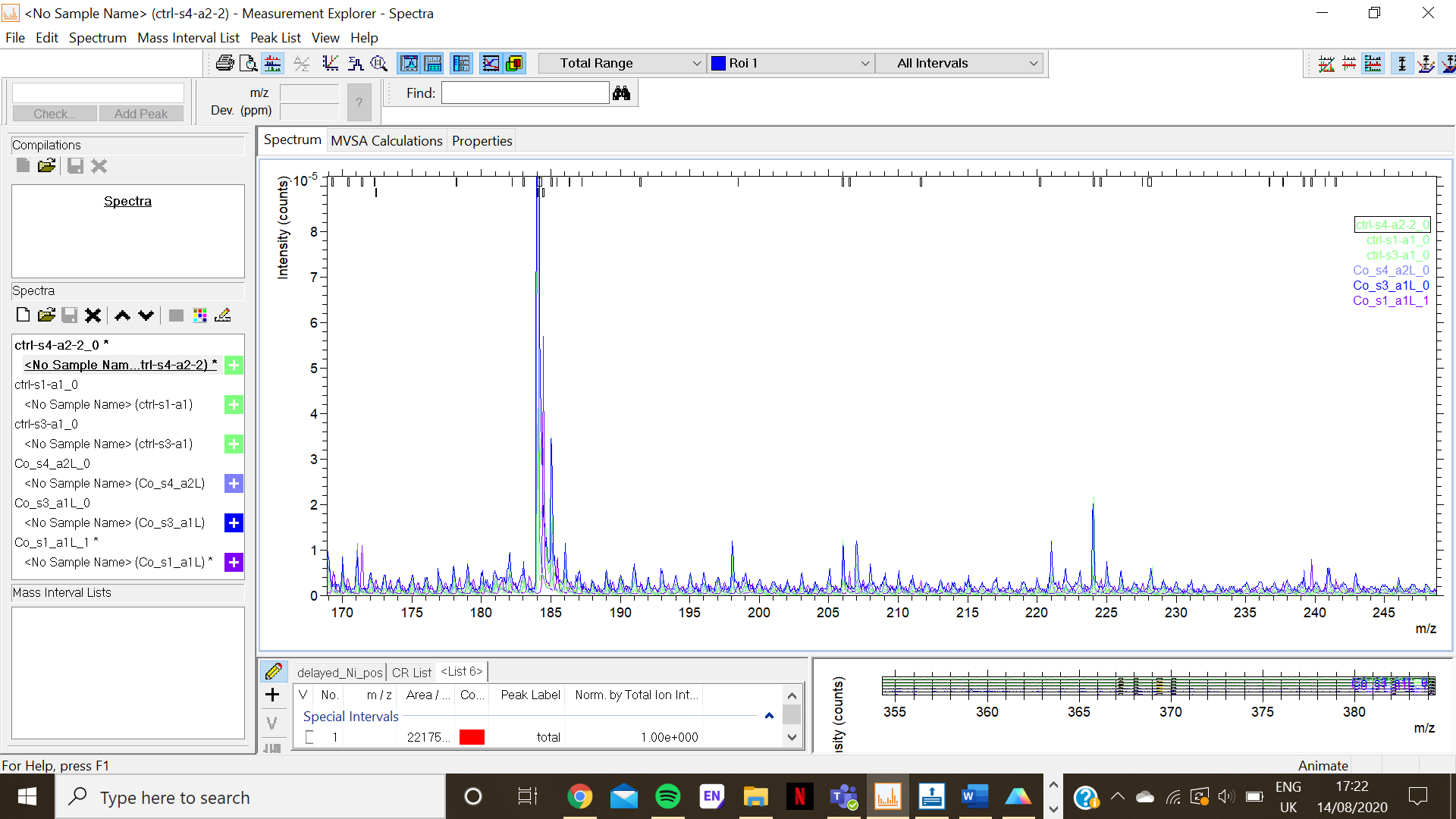

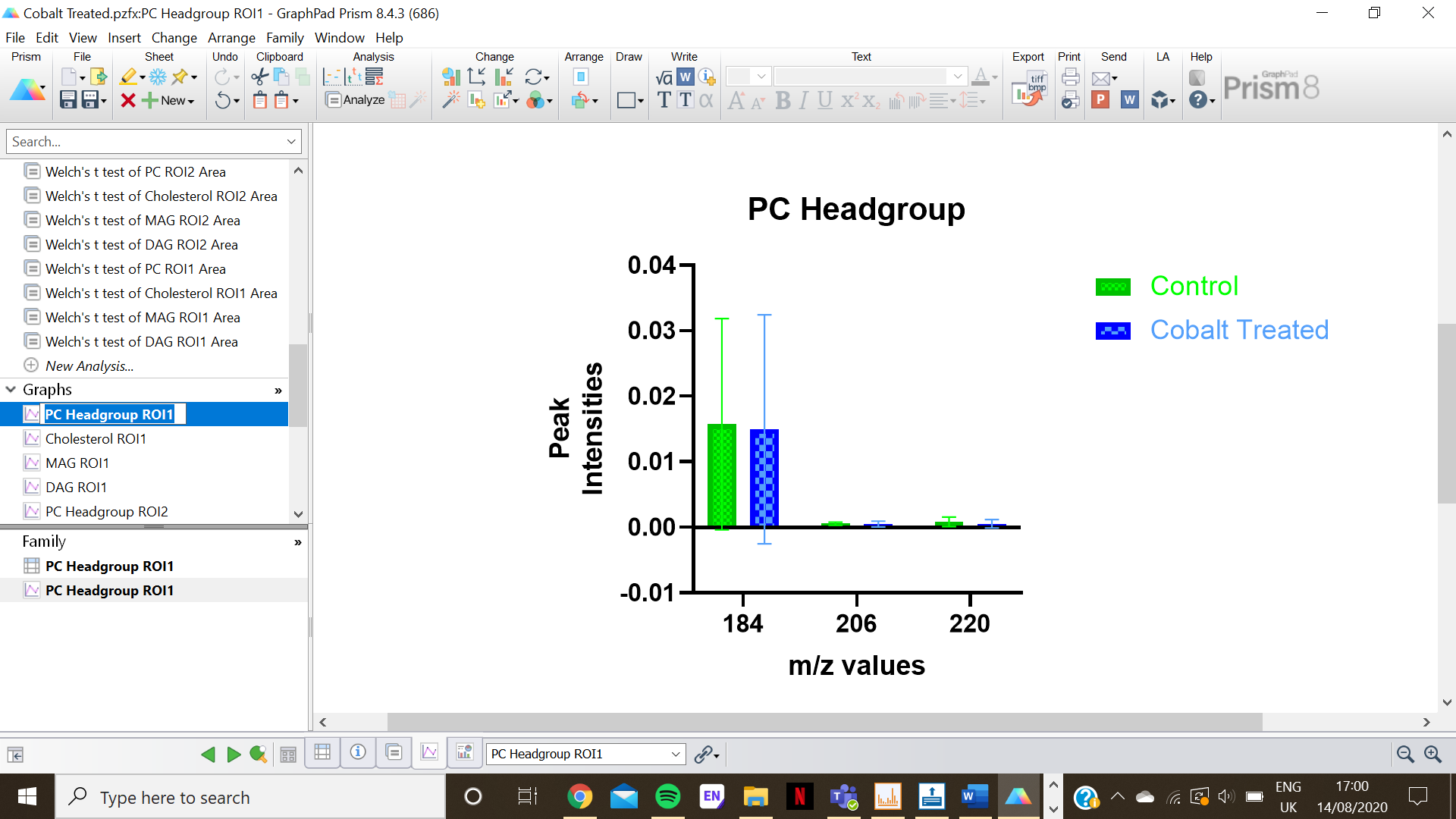


206 m/z C_5_H_14_NPO_4_Na

224 m/z C_8_H_19_NPO_4_Na

184 m/z C_5_H_15_NPO_4_

# Figure S18: Ion Profile: Cobalt Treated, Stratum Corneum: Cholesterol


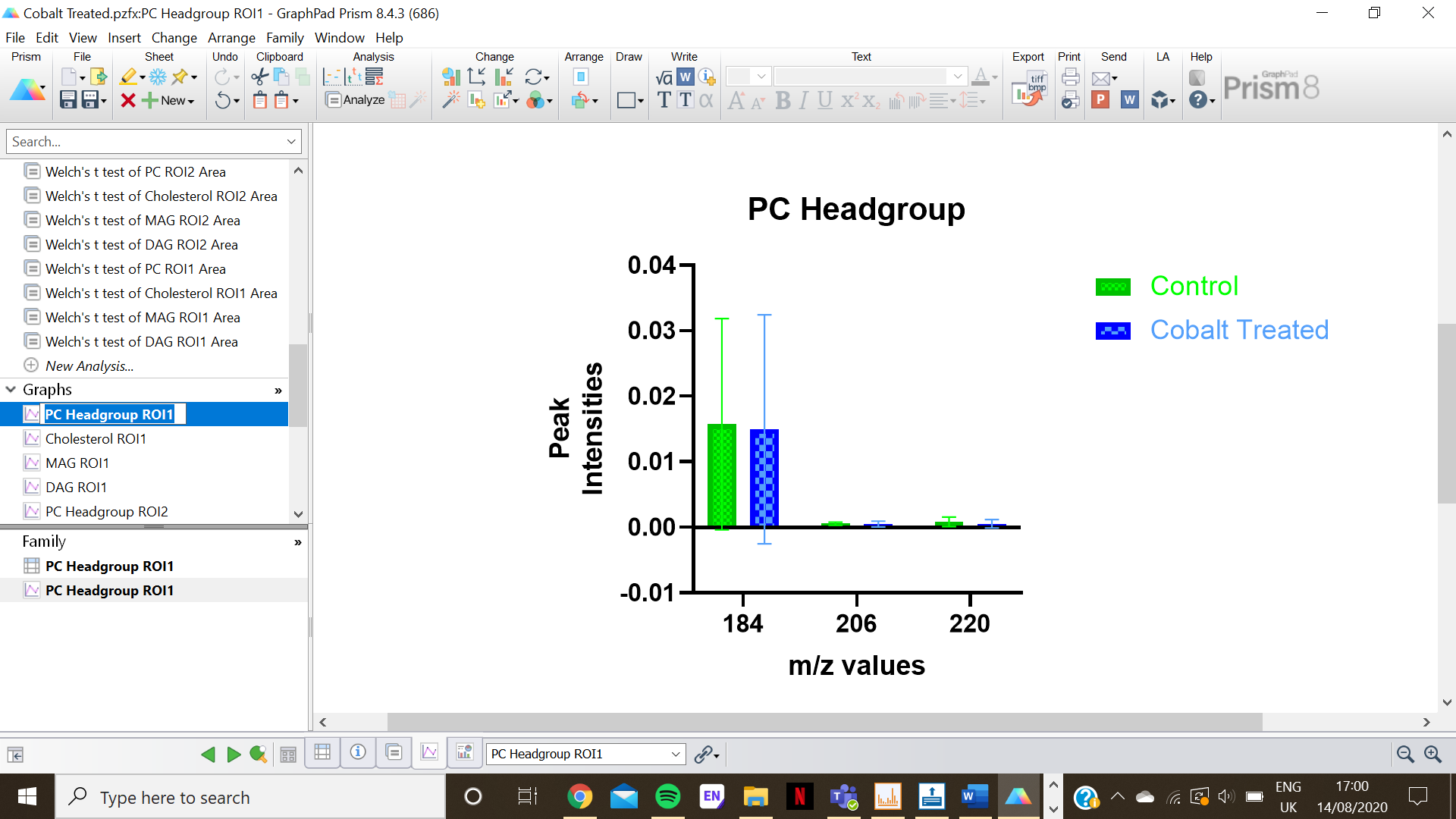

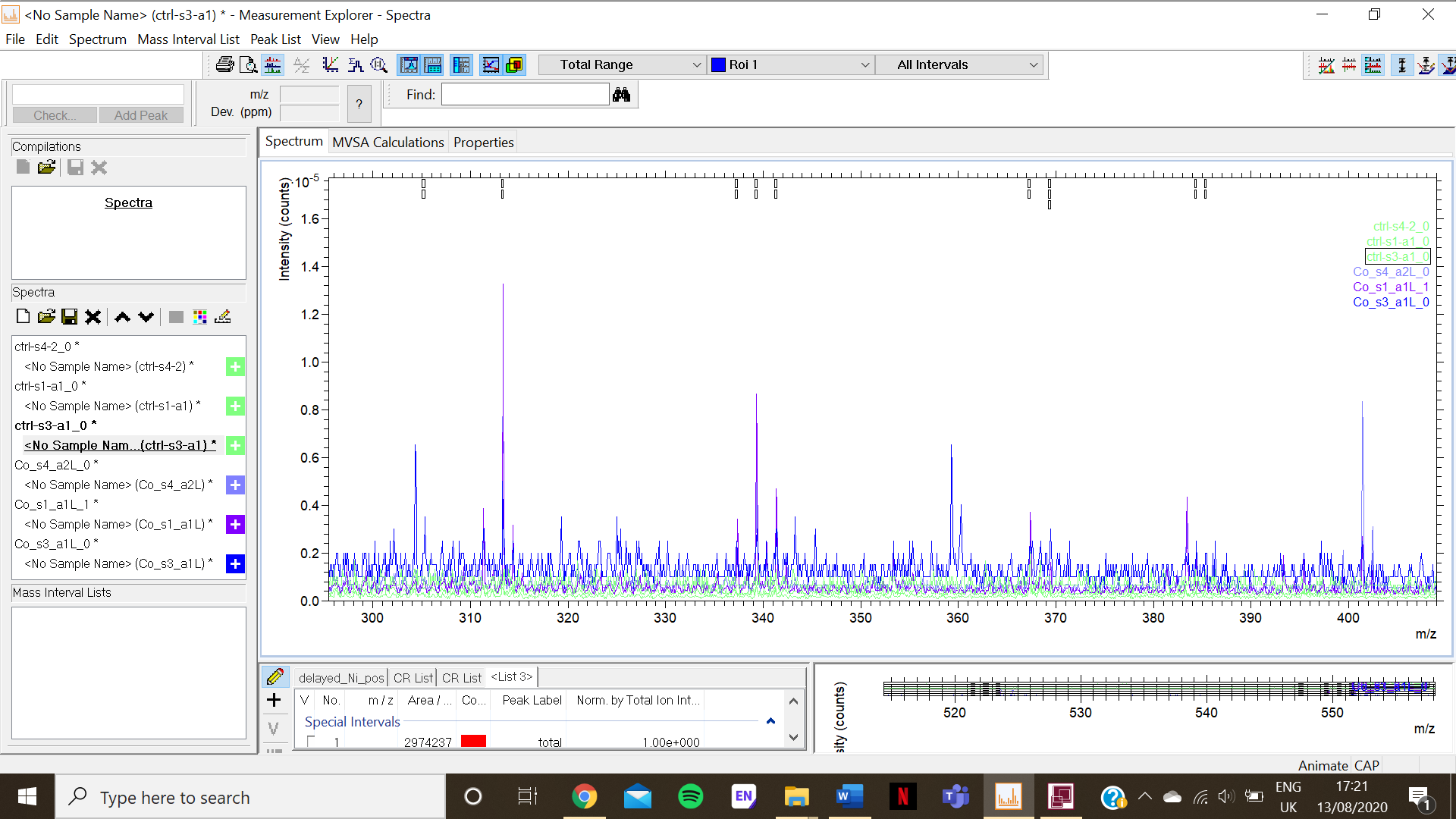


385 m/z C_27_H_45_O

384 m/z C_27_H_44_O

369 m/z C_27_H_45_

367 m/z C_27_H_43_

# Figure S19: Ion Profile: Cobalt Treated, Stratum Corneum: MAG

#
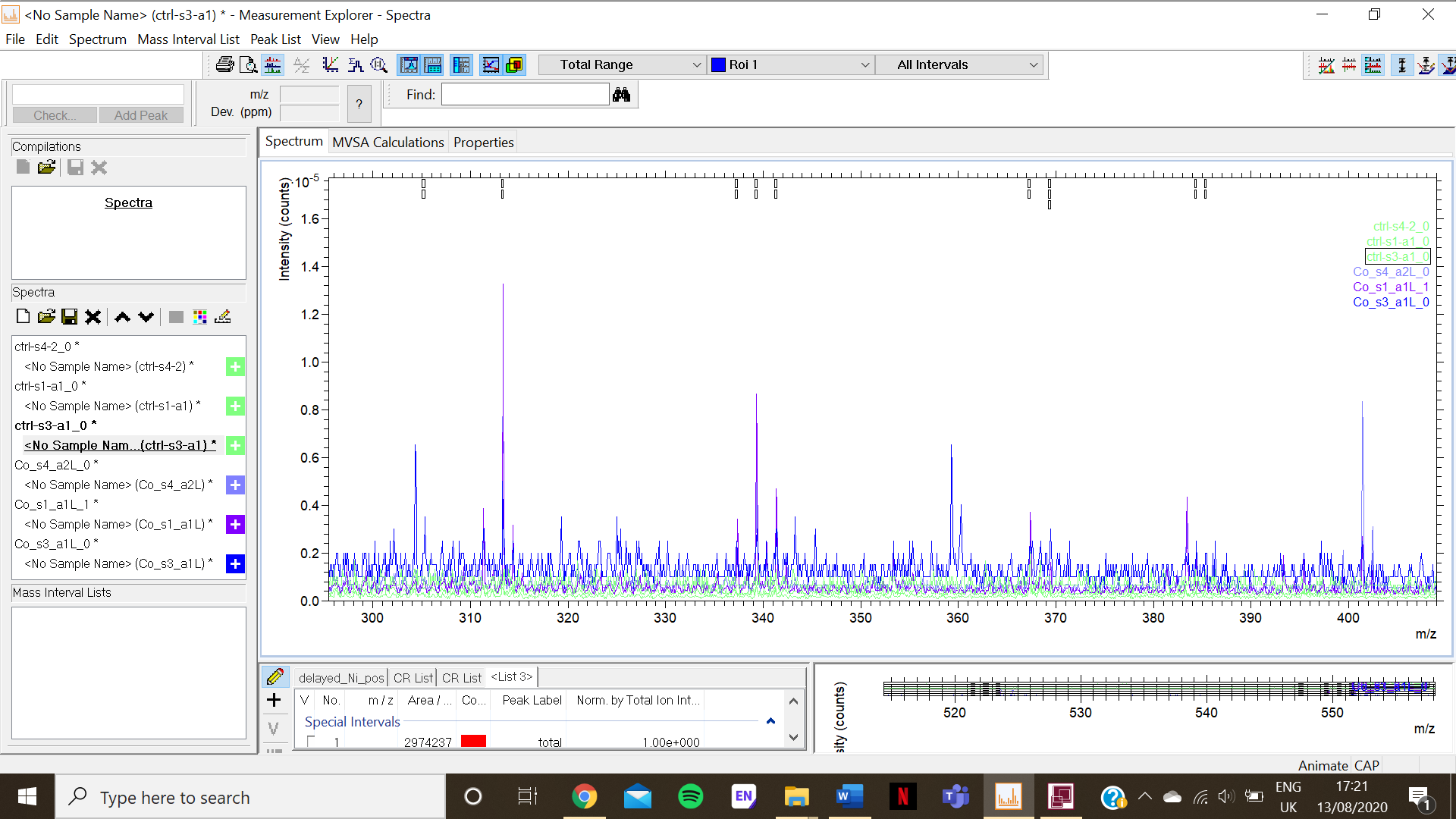

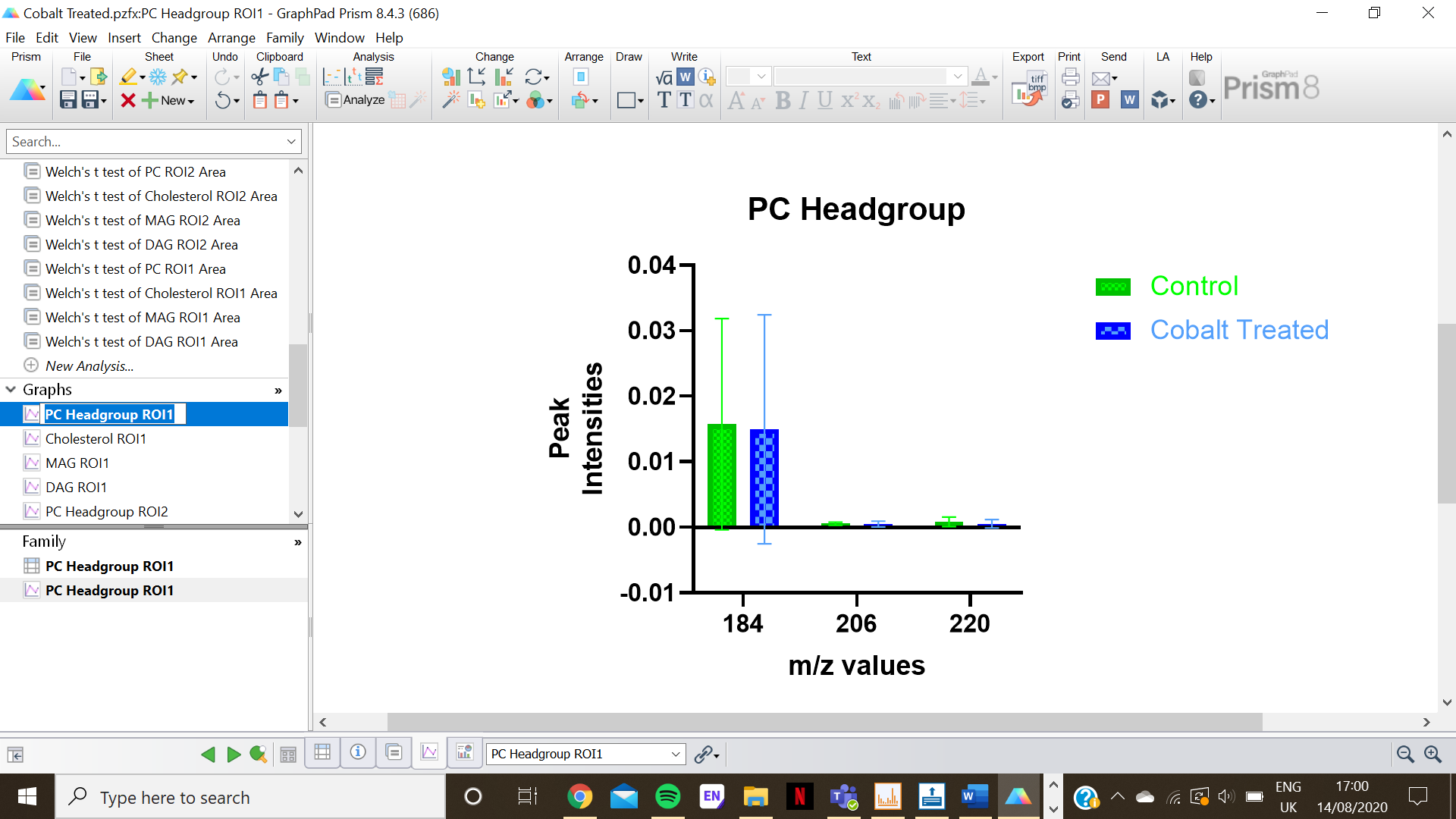


339 m/z C_21_H_39_O_3_

341 m/z C_21_H_41_O_3_

337 m/z C_21_H_37_O_3_

313 m/z C_19_H_37_O_3_

# Figure S20: Ion Profile: Cobalt Treated, Stratum Corneum: DAG
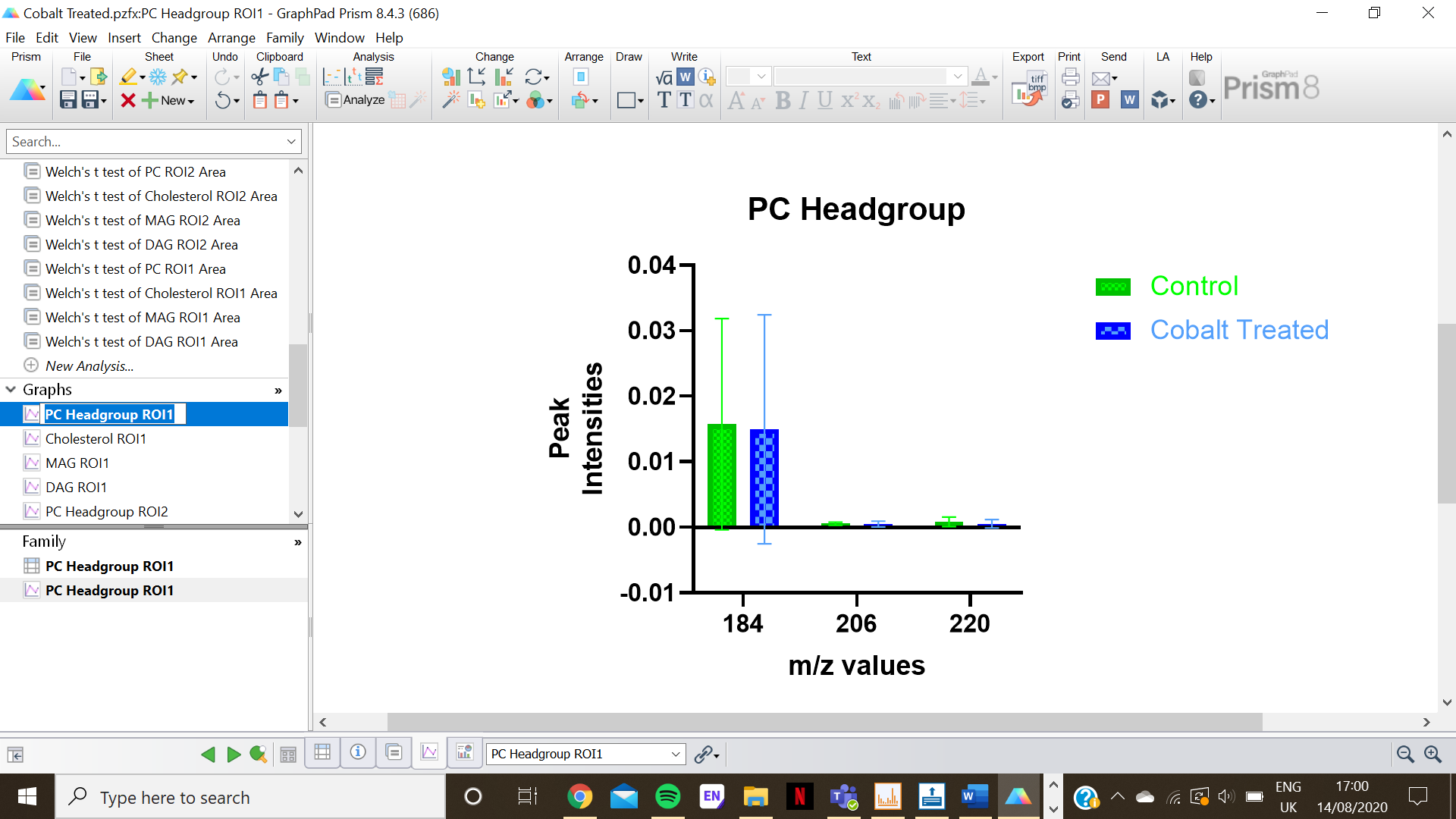

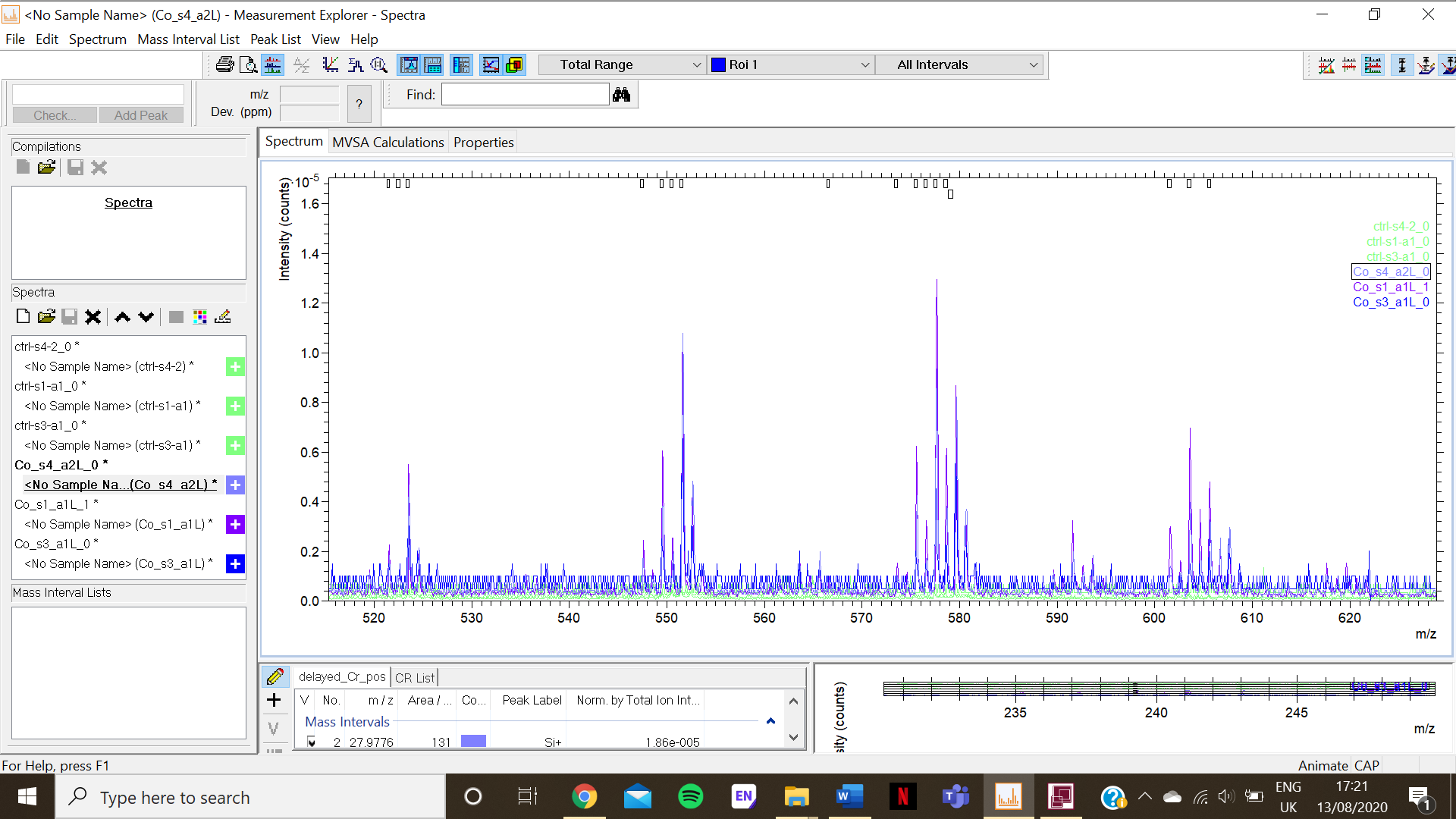


# Table S7. *m/z* peak area values for cobalt-treated skin in viable epidermis, normalised by corrected peak area (positive ion mode)

|  | ***Control Samples*** | | | ***Cobalt-Treated Samples*** | | |
| --- | --- | --- | --- | --- | --- | --- |
| ***m/z*** | **Control 1** | **Control 2** | **Control 3** | **Cobalt 1** | **Cobalt 2** | **Cobalt 3** |
| **PC Headgroup** |  |  |  |  |  |  |
| **184** | 71119.65 | 801932.7 | 1524940 | 190787.7 | 665336.9 | 1183.43 |
| **206** | 4969.37 | 13916.41 | 36715.88 | 6016.47 | 10439.49 | 1059.92 |
| **224** | 4149.65 | 37117.42 | 74926.26 | 5647.76 | 27389.37 | 1122.44 |
|  |  |  |  |  |  |  |
| **Cholesterol** |  |  |  |  |  |  |
| **367** | 452.18 | 915.24 | 1023.24 | 2248.37 | 975.24 | 1316.99 |
| **369** | 397.16 | 876.21 | 1009.22 | 1279.65 | 1032.32 | 2797.7 |
| **384** | 236.08 | 272.04 | 436.07 | 1825.23 | 325.05 | 6375.91 |
| **385** | 278.12 | 428.07 | 504.1 | 1463.87 | 395.09 | 3332.95 |
|  |  |  |  |  |  |  |
| **Vitamin E** |  |  |  |  |  |  |
| **430** | 208.05 | 299.04 | 420.05 | 371.09 | 244.03 | 1864.92 |
|  |  |  |  |  |  |  |
| **MAG** |  |  |  |  |  |  |
| **313** | 571.31 | 827.22 | 864.2 | 3800.43 | 818.22 | 1057.36 |
| **337** | 343.14 | 513.11 | 721.14 | 1466.86 | 533.13 | 1000.77 |
| **339** | 384.16 | 533.11 | 565.1 | 2937.23 | 651.17 | 1321.77 |
| **341** | 451.24 | 623.15 | 978.26 | 1585.89 | 594.15 | 1972.76 |
|  |  |  |  |  |  |  |
| **DAG** |  |  |  |  |  |  |
| **547** | 145.02 | 187.01 | 270.02 | 722.16 | 146.01 | 995.62 |
| **549** | 194.04 | 183.02 | 286.03 | 2025.79 | 189.02 | 1617.63 |
| **551** | 299.08 | 389.07 | 524.1 | 3130.8 | 346.05 | 3101.05 |
| **573** | 126.02 | 177.01 | 273.02 | 525.09 | 165.01 | 755.36 |
| **575** | 142.02 | 150.01 | 206.01 | 2135.87 | 158.01 | 1003.45 |
| **577** | 169.03 | 194.01 | 216.02 | 5327.64 | 242.02 | 1944.98 |
| **579** | 154.03 | 216.03 | 356.05 | 424.26 | 268.02 | 37767.29 |
| **601** | 73.01 | 120.01 | 187.01 | 982.22 | 133.01 | 837.63 |
| **603** | 115.02 | 141.01 | 163.01 | 2534.16 | 156.01 | 1171.64 |
| **605** | 105.01 | 123.01 | 146.01 | 1420.55 | 145.01 | 1969.7 |

# Figure S21: Ion Profile: Cobalt Treated, Viable Epidermis: PC Headgroup


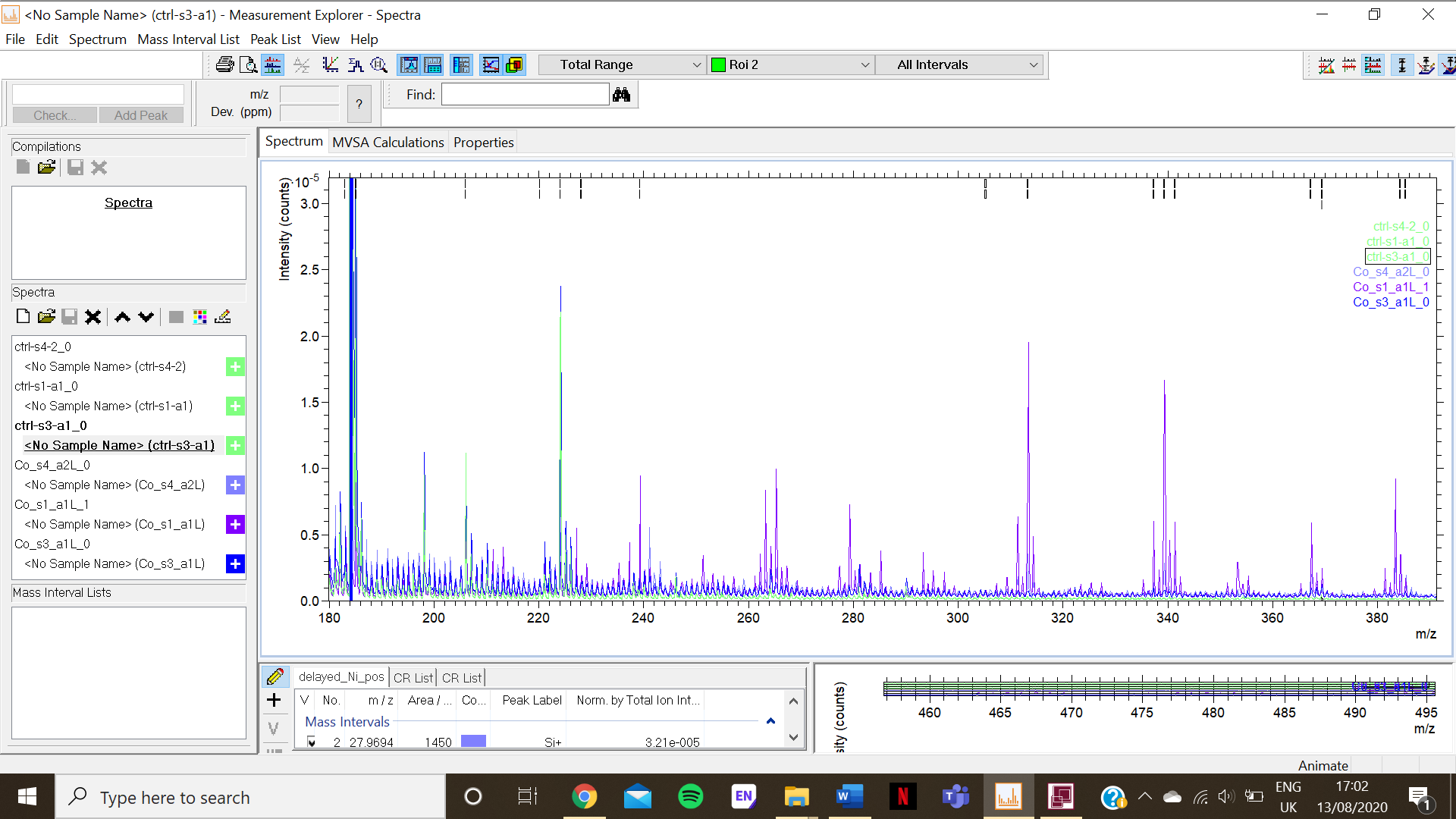

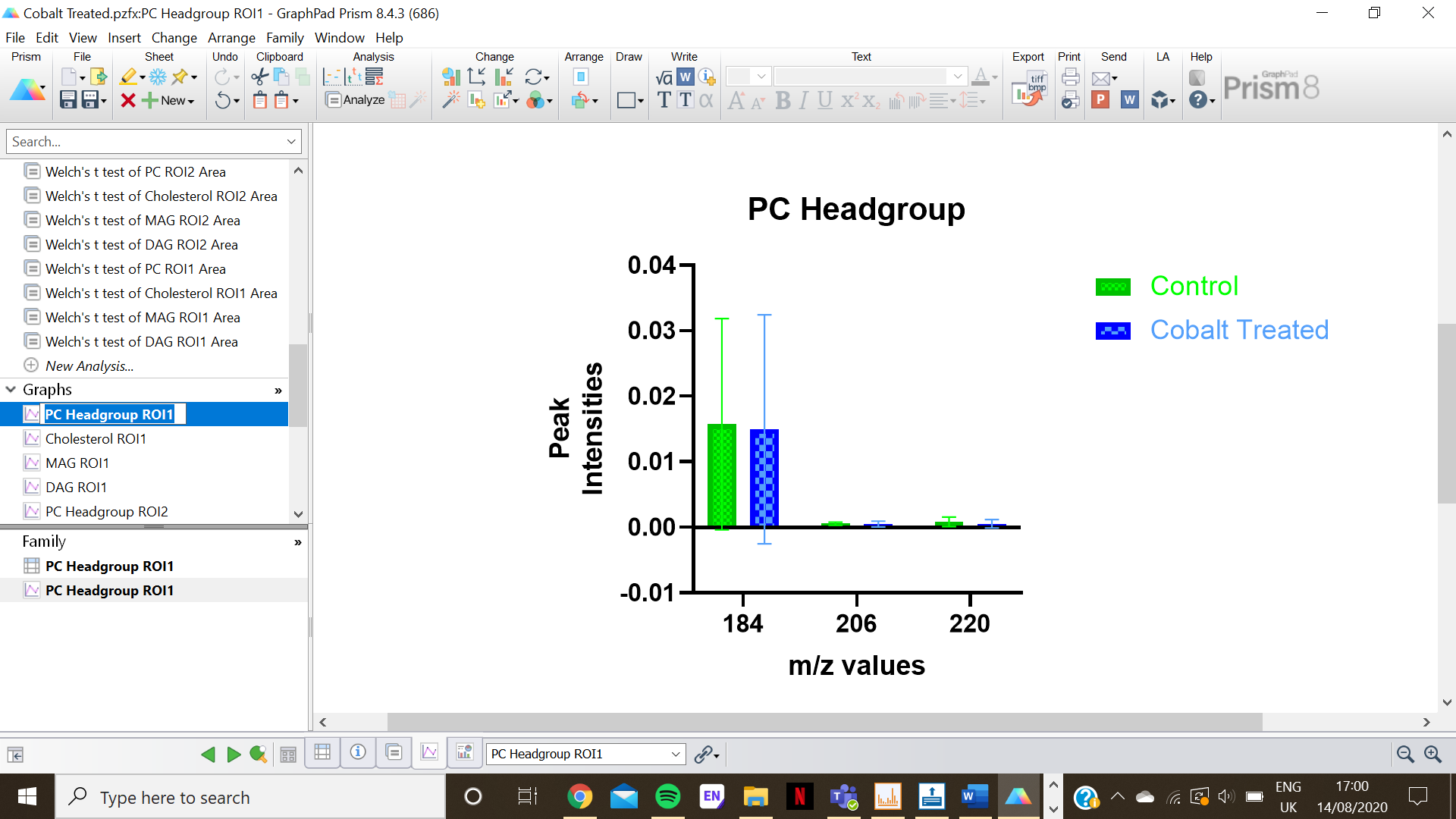


206 m/z C_5_H_14_NPO_4_Na

184 m/z C_5_H_15_NPO_4_

224 m/z C_8_H_19_NPO_4_Na

# Figure S22: Ion Profile: Cobalt Treated, Viable Epidermis: Cholesterol


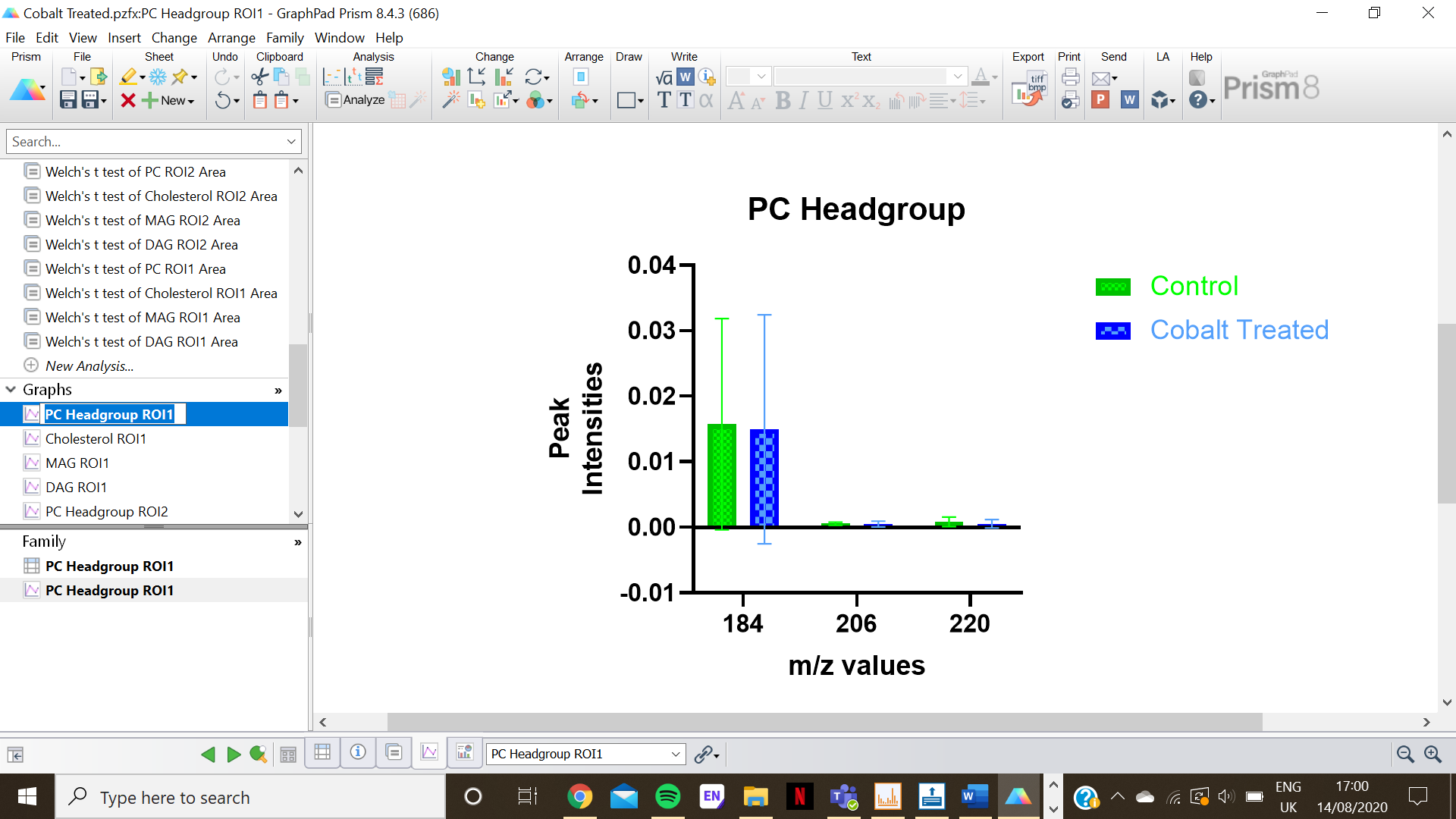

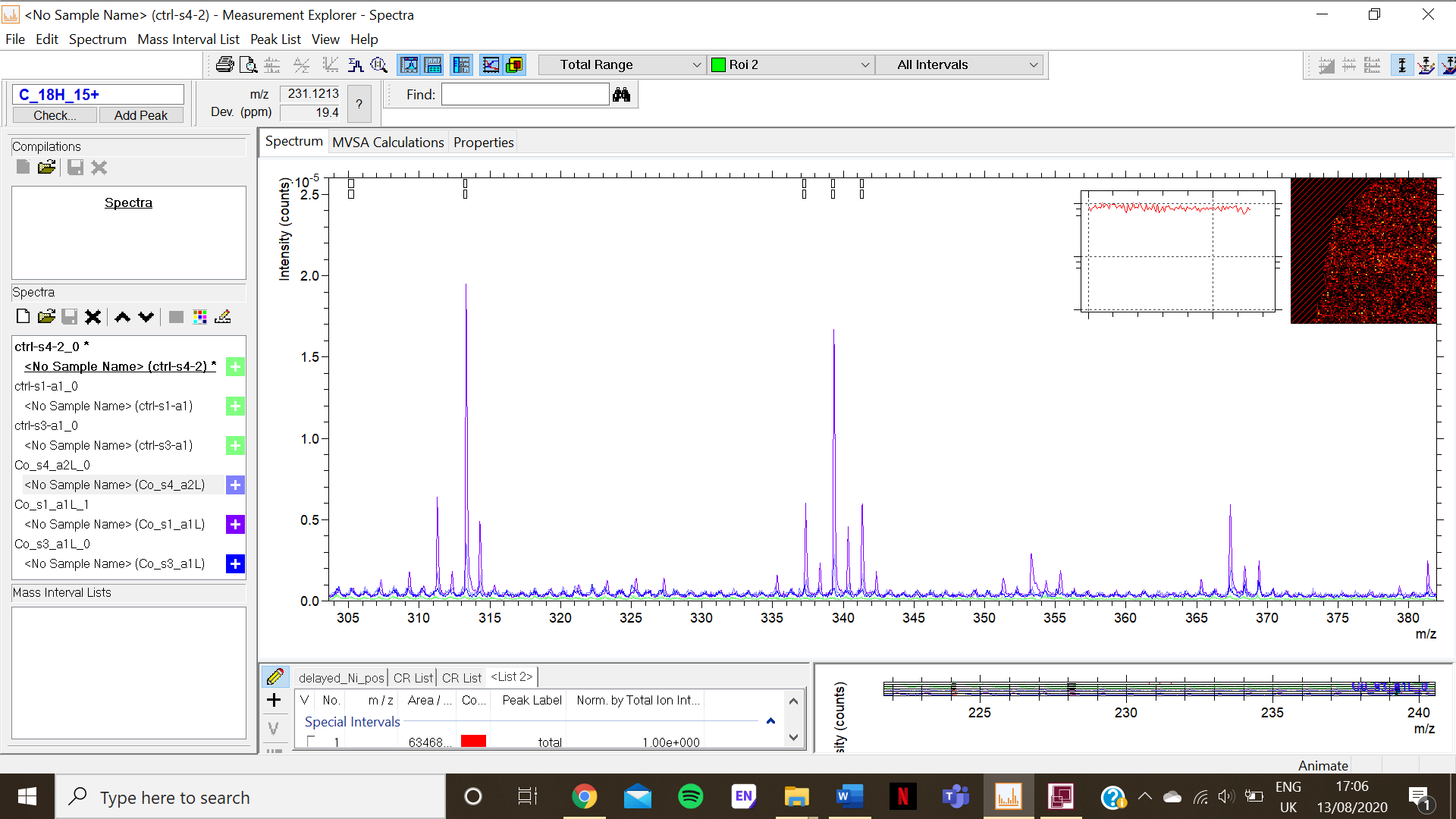


367 m/z C_27_H_43_

369 m/z C_27_H_45_

# Figure S23: Ion Profile: Cobalt Treated, Viable Epidermis: MAG


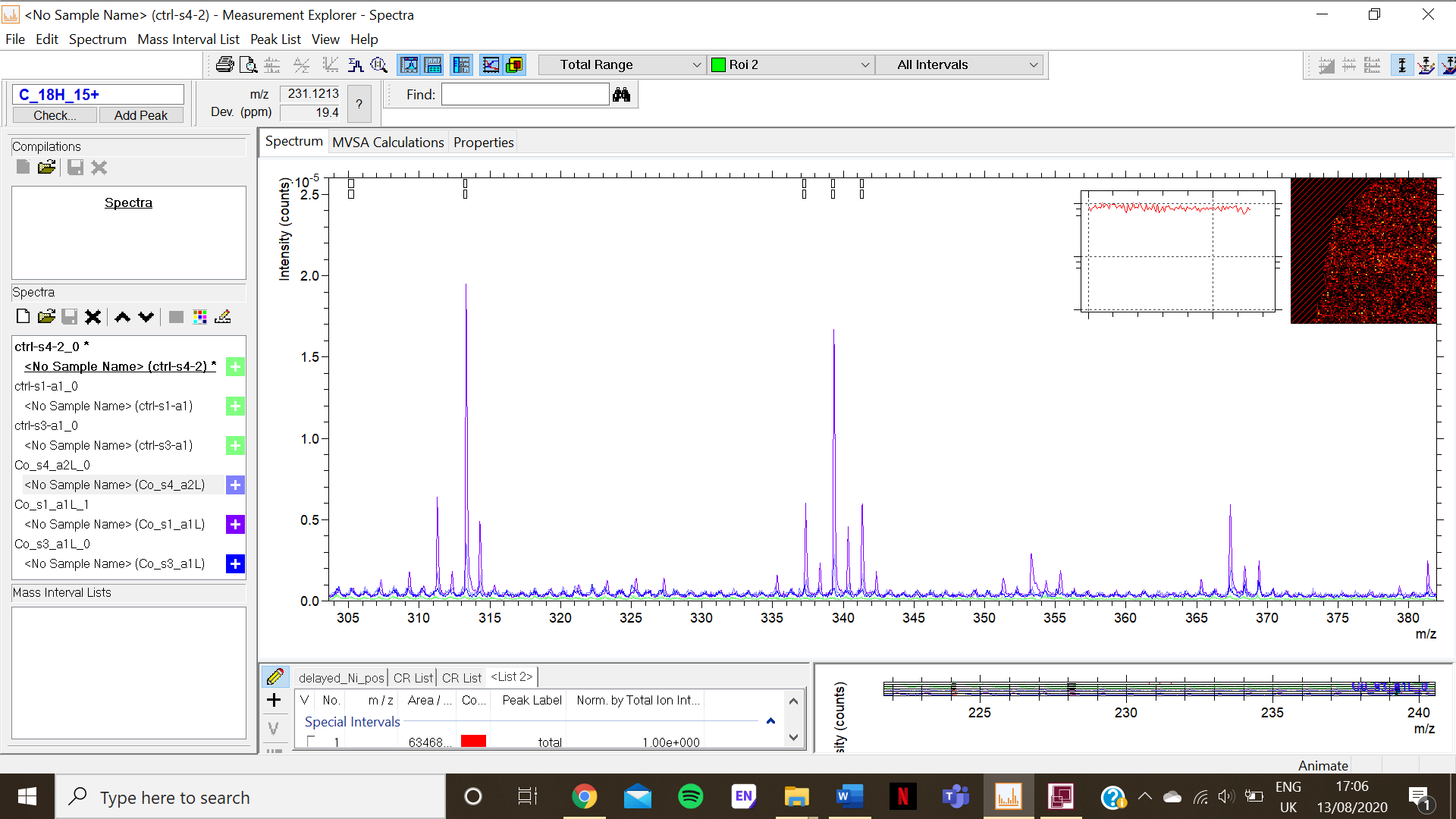

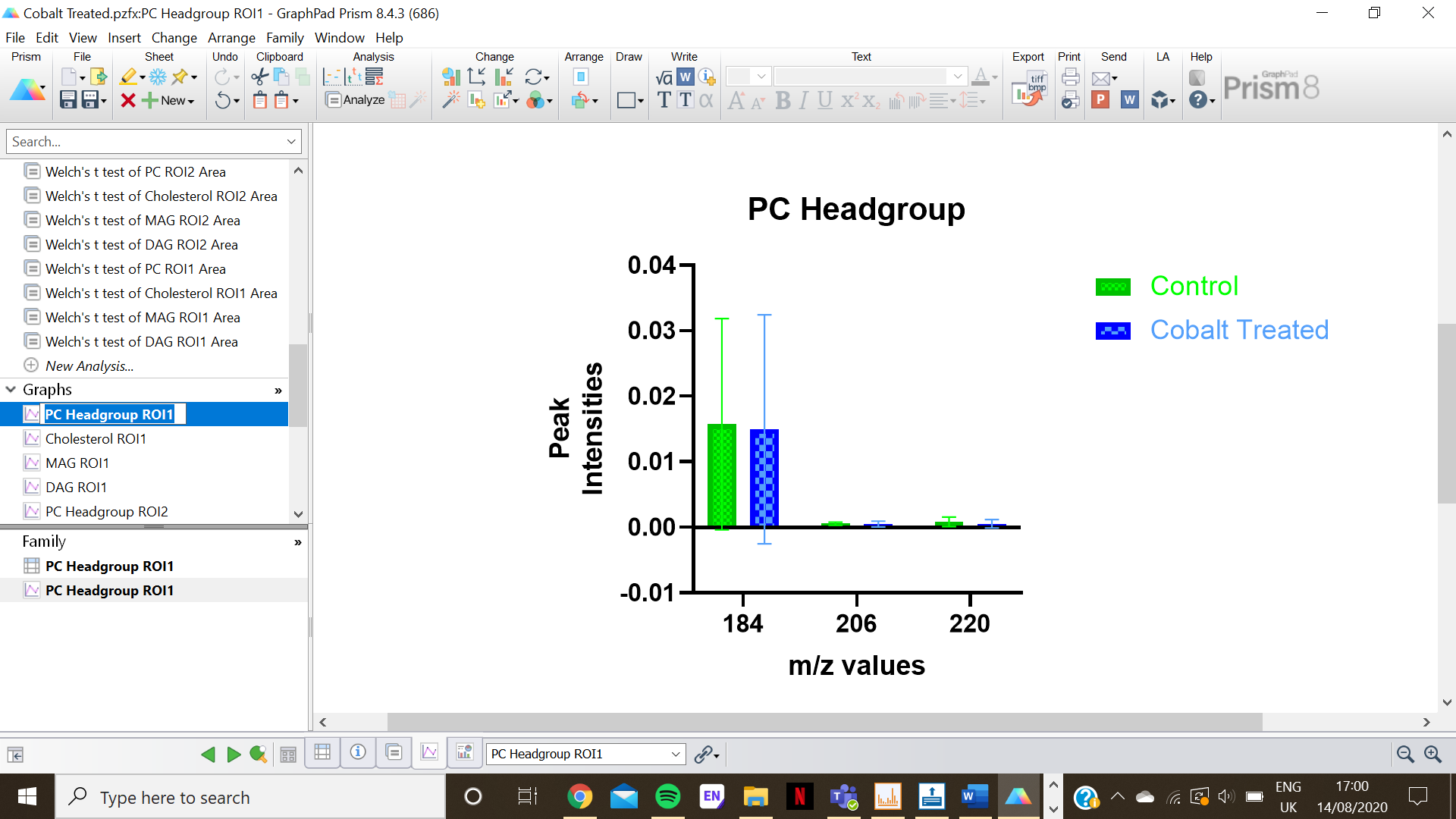


337 m/z C_21_H_37_O_3_

339 m/z C_21_H_39_O_3_

341 m/z C_21_H_41_O_3_

313 m/z C_19_H_37_O_3_

# Figure S24: Ion Profile: Cobalt Treated, Viable Epidermis: DAG


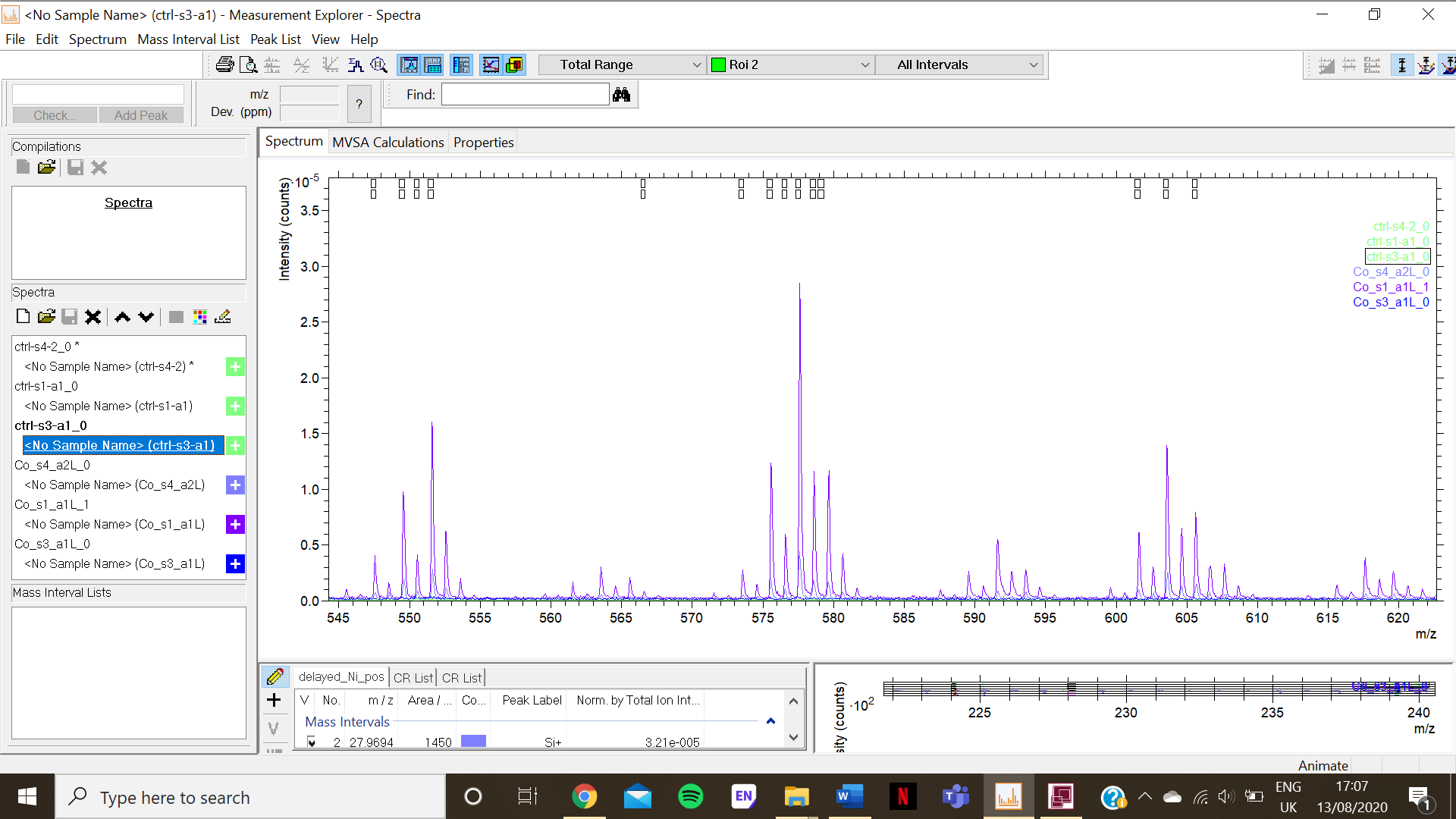

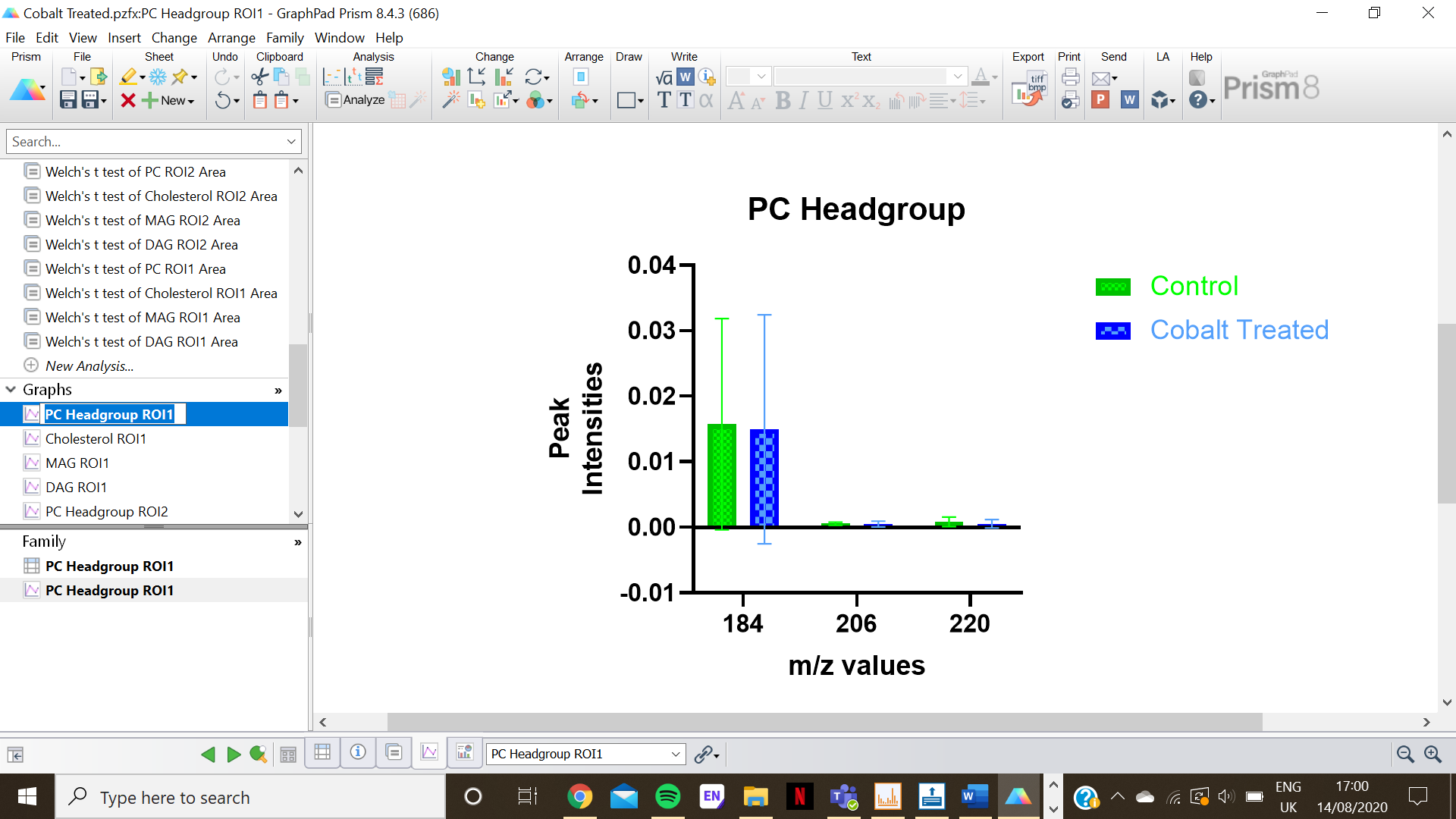


# Figure S25. A. 2D Score plot and B. loadings plot for partial least squares discriminant analysis (PLS-DA) of ToF-SIMS data of metal-treated *ex vivo* human skin. Data was extracted and analysed as normalised intensity (to total ion count).

**A.**


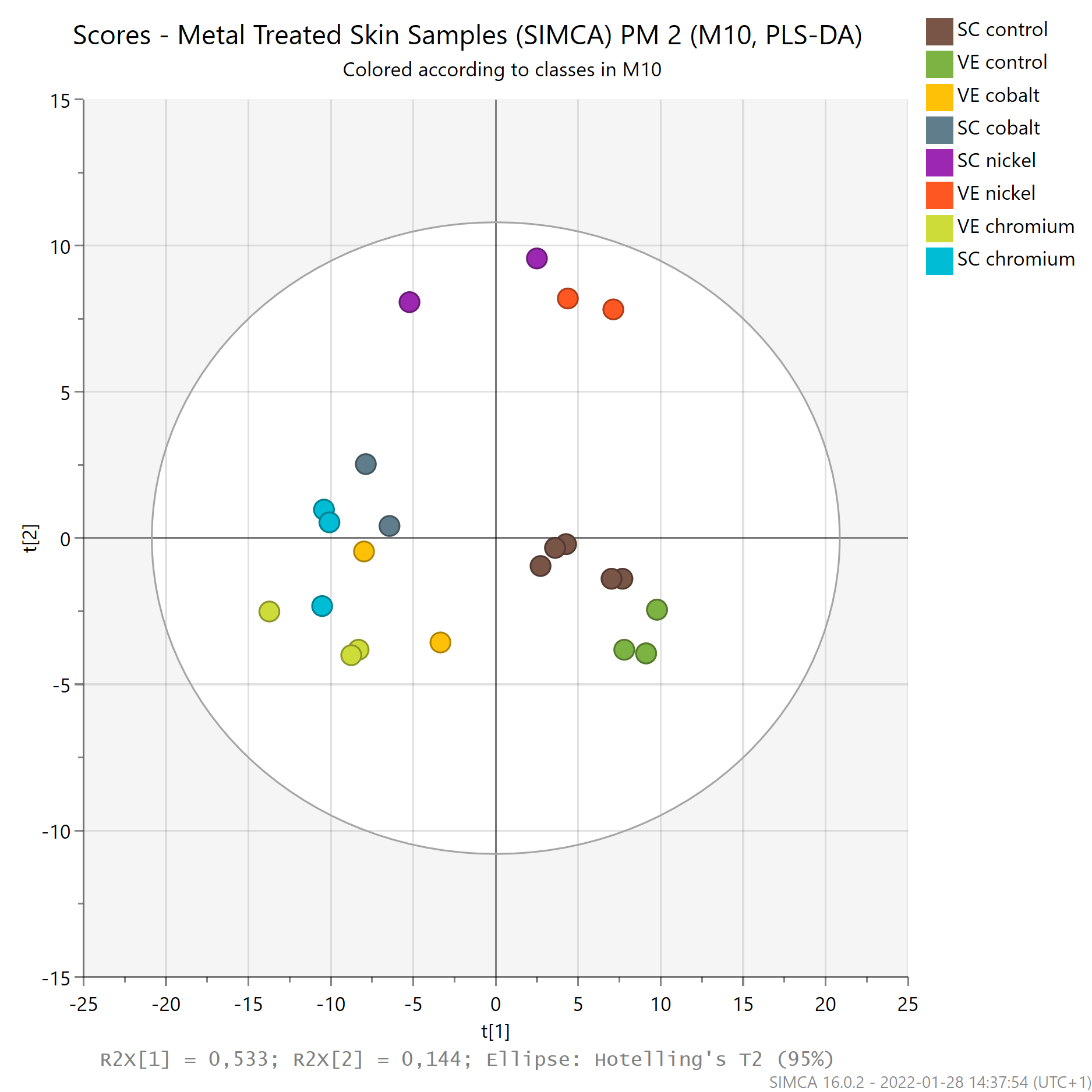


**B.**


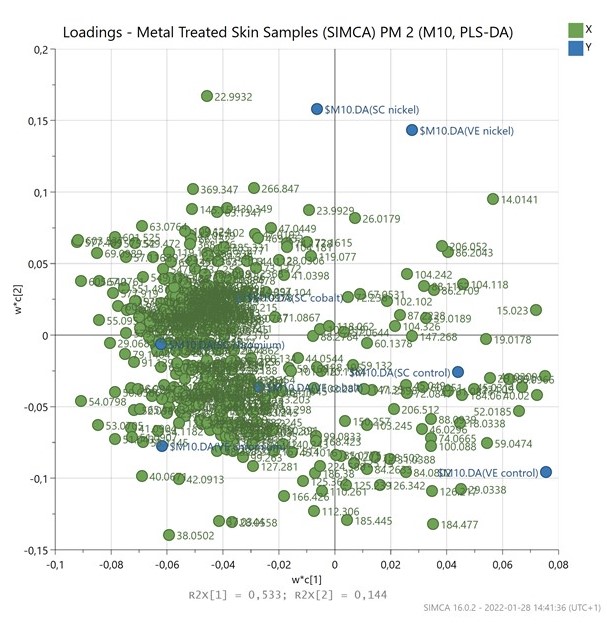

Supplement: Supplementary file 1 [file DataSheet1.docx]
